# Straightforward Access to Enantioenriched *cis*-3-Fluoro-dihydroquinolin-4-ols Derivatives via Ru(II)-Catalyzed-Asymmetric Transfer Hydrogenation/Dynamic Kinetic Resolution

**DOI:** 10.3390/molecules27030995

**Published:** 2022-02-01

**Authors:** Ricardo Molina Betancourt, Phannarath Phansavath, Virginie Ratovelomanana-Vidal

**Affiliations:** CSB2D Team, CNRS, UMR 8060, Chimie ParisTech, Institute of Chemistry for Life and Health Sciences, PSL University, 75005 Paris, France; r.molina-betancourt@chimieparistech.psl.eu

**Keywords:** asymmetric catalysis, reduction, fluorine, hydrogen transfer, ruthenium

## Abstract

Herein we report a practical method for the asymmetric transfer hydrogenation/dynamic kinetic resolution of *N*-Boc 3-fluoro-dihydrotetrahydroquinolin-4-ones into the corresponding *cis*-fluoro alcohols in 70–96% yields, up to 99:1 diastereomeric ratio (dr) and up to >99% ee (enantiomeric excess) by using the ruthenium complex Ts-DENEB and a formic acid/triethylamine (1:1) mixture as the hydrogen donor under mild conditions.

## 1. Introduction

The number of bioactive molecules containing fluorine approved by the Food and Drug Administration (FDA) has greatly increased over time. The first decade of this century saw the introduction of 40 new compounds having a fluorine atom. This number represented at the time an important increase of 20% of the commercial drugs bearing at least one fluorine atom in their structure [1]. Compared to this, in 2018 alone, 17 fluorine-containing pharmaceuticals were approved by the FDA [2]. In a single year, the number of new fluorinated drugs covered almost half of the increase of a whole decade.

This fluorine rush is easily explained by fluorine’s ability to affect important parameters for a drug candidate, such as permeability or pK_a_, and to modify its pharmacokinetics and pharmacodynamics. Fluorine is thus an element that gives control to medicinal chemists over tailoring the properties of a molecule. Organic chemists must then work in consideration of this increasing demand and develop new methodologies to introduce fluorine into complex molecules and into versatile building blocks [3].

**Scheme 1:**
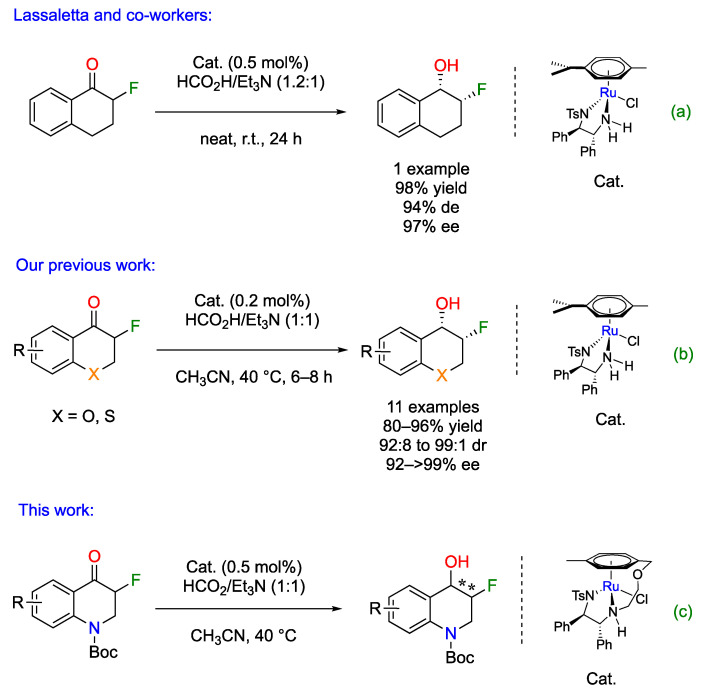
(**a**) ATH/DKR to access enantiomerically enriched fluoro-tetralol; (**b**) ATH/DKR of 3-fluoro-chromanone derivatives; (**c**) ATH/DKR of *tert*-butyl 3-fluoro-4-oxo-3,4-dihydroquinoline-1(2*H*)-carboxylates.

The tetrahydroquinoline core is a very common scaffold found in several biologically active molecules and therapeutic agents [4]. Introducing a fluorine atom in its structure would bring a novelty into the medicinal chemist toolbox and, in theory, open up its use in the screening and development of new biologically active molecules. Asymmetric transfer hydrogenation (ATH) has been reported by Lassaletta and co-workers [5] as a method to access enantiomerically enriched fluoro-tetralol via a dynamic kinetic resolution (DKR) process (Scheme 1a). Following the premise of the fluorine need and the previous works in the laboratory including the ATH/DKR of 3-fluoro-chromanone derivatives [6,7,8,9,10,11,12,13,14] (Scheme 1b), we herein report an asymmetric transfer hydrogenation/dynamic kinetic resolution [15,16,17,18,19,20,21,22,23,24] (ATH/DKR) of *tert*-butyl 3-fluoro-4-oxo-3,4-dihydroquinoline-1(2*H*)-carboxylates into the corresponding enantioenriched *cis*-fluorohydrins (Scheme 1c).

## 2. Results and Discussion

**Scheme 2:**
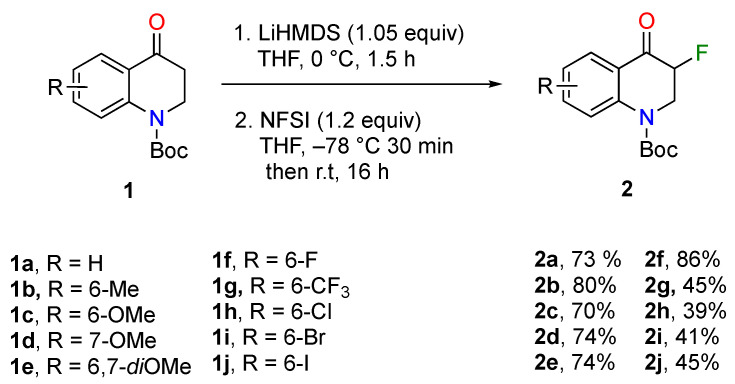
Synthesis of fluorinated dihydroquinolin-4-ones **2a**–**j**.

The study began with the synthesis of fluorinated dihydroquinolin-4-ones **2a**–**j** via an electrophilic fluorination of the *N*-Boc-protected heterocyclic ketones **1a**–**j** by using lithium bis(trimethylsilyl)amide as a base and NFSI as the fluorine source (Scheme 2). 

3-Fluoro-dihydroquinolin-4-one carboxylate **2a** was chosen as the standard substrate for the optimization of asymmetric transfer hydrogenation. Based on previous studies, the ATH was set at 40 °C in acetonitrile with a (1:1) molar mixture of formic acid and triethylamine as the hydrogen source. Under these conditions, a set of commercially available Ru(II) catalysts and a Rh(III) complex were screened (Table 1). After 3 h with a catalyst loading of 0.5 mol%, the Ru and Rh catalysts allowed a full conversion of the α-fluoro ketone into the corresponding fluoro alcohol **3a**. All the complexes delivered excellent isolated yields and enantiomeric excesses for the desired fluorohydrin **3a** (entries 1 to 4). The main difference appeared in the diastereoselectivity outcome. Whereas (*R,R*)-Rh-*teth*-TsDPEN ((*R,R*)-**A**; entry 1) led to a moderate 79:21 dr, (*R,R*)-Ru(*p*-cymene)TsDPEN ((*R,R*)-**B**; entry 2), (*R,R*)-Ru(mesitylene)TsDPEN ((*R,R*)-**C**; entry 3) and (*R*,*R*)-TsDENEB ((*R,R*)-**D**; entry 4), all gave an excellent diastereoisomeric ratio dr of 97:3 or 98:2, indicating that the reaction was neither affected by the nature of the arene ligand nor the tethered or untethered characteristic of the complex. From this series of results, the (*R*,*R*)-TsDENEB ((*R,R*)-**D**) catalyst was chosen for the optimization conditions.

The solvent of the asymmetric transfer hydrogenation was then investigated. By using a catalyst loading of 0.5 mol% of (*R*,*R*)-**D**, formic acid/triethylamine (1:1) mixture and a reaction time of 3 h, we tested a series of different solvents (Table 2). Several polar aprotic (CH_3_CN, CH_2_Cl_2_, THF, Me-THF and EtOAc) and polar protic (MeOH, *i*PrOH and HFIP) as well as aromatic solvents were screened (toluene, chlorobenzene and trifluoromethylbenzene). The diastereo- and enantioselectivities remained mostly unchanged in all the tests. The conversion rate and the yield showed to be highly dependent on the solvent used. Isopropanol and acetonitrile led to 95% and 100% conversions, respectively, with excellent stereoselectivities in both solvents, acetonitrile slightly surpassing isopropanol in all of them. This prompted us to set acetonitrile as the solvent for the asymmetric transfer hydrogenation.

We continued the optimization by varying the hydrogen source of the reaction (Table 3). The acid-to-base ratio was examined (entries 1 to 4). Changing the formic acid to triethylamine ratio from 1:1 to 2:5 (entry 2) showed no impact on the reaction. However, switching to a HCO_2_H/Et_3_N ratio of 5:2 (entry 3) was detrimental to the diastereoselectivity which dropped to a 54:46 dr. Moreover, when this ratio was further increased to 12:1 (entry 4), no conversion was observed. This strong influence of the acid/base ratio of the hydrogen donor reflects the one that was observed in the ATH/DKR of 3-fluorochromanones [6]. Other organic bases associated to formic acid (DBU, entry 5, and DABCO, entry 6) as well as formate salts (ammonium formate and calcium formate, entries 7 and 8, respectively) were screened, all giving lower conversions and similar stereoselectivities than Et_3_N. Finally, the catalyst loading (0.2 mol%; entry 9) and the reaction temperature (room temperature; entry 10) were lowered. Both tests gave lower conversions in spite of longer reaction times, so we set the optimized conditions for the ATH/DKR as 0.5 mol% of (*R*,*R*)-**D** as the catalyst, HCO_2_H/Et_3_N (1:1) mixture as the hydrogen donor, acetonitrile as the solvent and 40 °C as the reaction temperature.

**Scheme 3:**
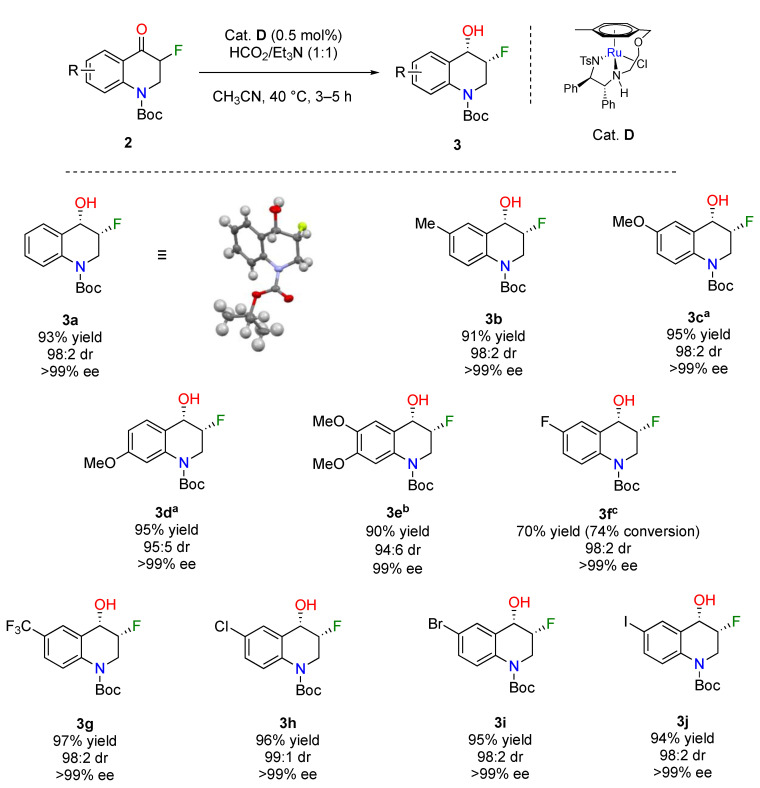
Scope of the reaction. Unless specified, the reaction time is 3 h. ^a^ Reaction time of 5 h. ^b^ 1.0 mol% of catalyst and reaction time of 24 h. ^c^ Reaction time of 24 h.

Once the aforementioned conditions were identified, we subjected the family of substituted *tert*-butyl 3-fluoro tetrahydroquinoline-4-ones carboxylates (**2a**–**j**) previously synthesized to the asymmetric transfer hydrogenation. The scope (Scheme 3) showed that the fluoro dihydrotetrahydroquinolin-4-ones bearing electron-donating or electron-withdrawing groups all led to good yields (90–96%), diastereo- (94:6 to 99:1 dr) and enantioselectivities (99–> 99% ee) to the corresponding fluorohydrins (**3a**–**j**). Notably, the asymmetric transfer hydrogenation tolerated substituents such as chlorine, bromine or iodine along with other groups such as methyl, methoxy and trifluoromethyl. Concerning the outcome of the reaction, the two substrates bearing a methoxy- group in position 7 of the aromatic ring (**2d** and **2e**) gave a slightly lower diastereoselectivity compared to the rest of the dihydroquinolin-4-ones. The ATH of compound **2e,** possessing two methoxy groups in positions 6 and 7, also required a higher catalyst loading (1.0 mol%) and a longer reaction time (24 h) to achieve a full conversion. 

The absolute configuration of compound **3a** was unambiguously assigned as (3*R*, 4*S*) by the X-ray crystallographic analysis. We conjectured that the other products of the developed ATH/DKR reaction led to 3-fluoro-dihydrotetrahydroquinolin-4-ols derivatives with the same absolute configuration (Scheme 3).

**Scheme 4:**
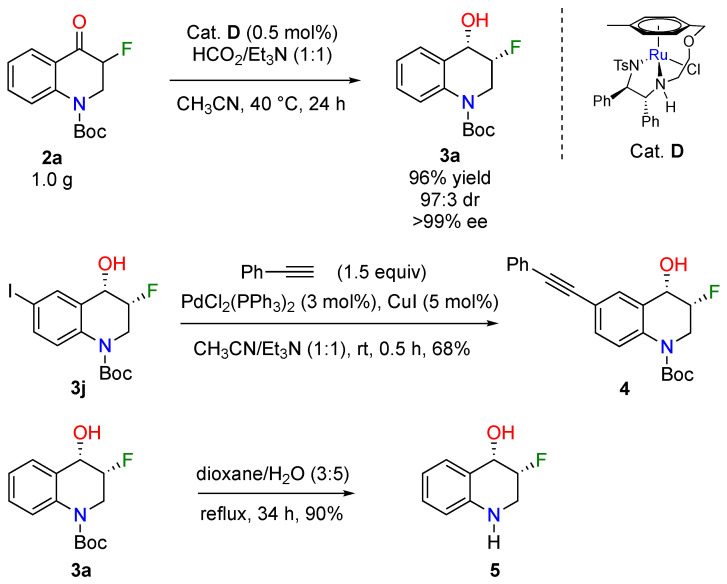
Scale-up, functionalization and deprotection experiments.

The usefulness of the studied asymmetric transfer hydrogenation was evaluated by performing the reaction on a gram-scale with substrate **2a** (Scheme 4). 1.0 g of *tert*-butyl 3-fluoro-dihydrotetrahydroquinolin-4-one carboxylate was subjected to the optimized reaction conditions which led to the *cis*-fluorohydrin **3a** in 96% yield, 97:3 dr and > 99% ee. In addition, the post-functionalization of compound **3j** using a Sonogashira cross-coupling reaction with phenylacetylene was carried out in the presence of PdCl_2_(PPh_3_)_2_ and CuI to afford the corresponding alkyne **4** in 68% yield. Finally, *N*-Boc deprotection of compound **3a** was readily achieved by heating in dioxane/H_2_O and the desired amine **5** was isolated in 90% yield.

## 3. Materials and Methods

### 3.1. General Information

All air- and/or water-sensitive reactions were carried out under an argon atmosphere. THF, CH_2_Cl_2_ and toluene were dried over alumina columns in a solvent purification apparatus (Innovative Technology, Oldham, UK). Methanol, isopropanol, chlorobenzene, trifluoromethylbenzene, ethyl acetate and acetonitrile from Sigma-Aldrich (Darmstadt, Germany) were used without further purification. Hexafluoroisopropanol (HFIP) was purchased from TCI and was used without further purification. Formic acid/triethylamine (1:1) mixture was purchased from Fluka or Alfa Aesar and was used without further purification. Reactions were monitored by thin layer chromatography carried out on precoated silica gel plates (Merck 60F254, Darmstadt, Germany) and revealed with either an ultraviolet lamp (*λ* = 254 nm) or a potassium permanganate solution. Proton nuclear magnetic resonance (^1^H-NMR) spectra were recorded using a Bruker AC 400 (400 MHz). The chemical shifts are expressed in parts per million (ppm) referenced to residual chloroform (7.26 ppm). Data are reported as follows: chemical shifts (*δ*), multiplicity (recorded as s, singlet; d, doublet; t, triplet; q, quadruplet; quint, quintuplet; sext, sextuplet; hept, heptuplet; m, multiplet and br, broad), coupling constants and integration. Carbon-13 nuclear magnetic resonance (^13^C-NMR) spectra were recorded using a Bruker AC 400 (101 MHz). The chemical shifts are expressed in parts per million (ppm) relative to the centre line of the triplet at 77.16 ppm for CDCl_3_. Melting points (m. p.) were determined on a Köfler melting point apparatus. Optical rotations were measured on a Jasco P-1010 polarimeter. High resolution mass spectrometric (HRMS) analyses were measured on LTQ-Orbitrap (Thermo Fisher Scientific, Waltham, Massachusetts, US) at Sorbonne Université.

### 3.2. Method for the Synthesis of Compounds ***2***

To a 0 °C solution of LiHMDS (1.0 M in THF, 1.05 equiv) in a round-bottom tube set under argon, was slowly added a solution of the ketone (1.2 mmol, 1.0 equiv) in THF (2 mL) over 5 min. The mixture was stirred at 0 °C for 1.5 h. The resulting solution was then added dropwise over 5 min via a cannula to a −78 °C solution of NFSI (1.4 mmol, 1.2 equiv) in THF (4 mL). The reaction mixture was stirred at −78 °C for 30 min and then allowed to come to room temperature overnight. The reaction was diluted with 3 mL of CH_2_Cl_2_, quenched with 5 mL of saturated NH_4_Cl aqueous solution and extracted with CH_2_Cl_2_ (3 × 10 mL). The combined organic layers were washed with brine, dried over MgSO_4_, filtered, and concentrated under reduced pressure [25]. The pure products were isolated by flash column chromatography on silica gel (see Supplementary Materials for NMR spectra).

*tert*-Butyl 3-fluoro-4-oxo-3,4-dihydroquinoline-1(2*H*)-carboxylate (**2a**). 2.0 g of *tert*-butyl 4-oxo-3,4-dihydroquinoline-1(2H)-carboxylate (8.09 mmol, 1.0 equiv) were used following the described procedure. Purification via flash column chromatography on silica gel (95:5 petroleum ether/ethyl acetate) yielded 1.56 g of **2a** as a white solid (73%). m.p. 96–100 °C. ^1^H-NMR (400 MHz, Chloroform-*d*) δ 8.03 (dd, *J* = 8.0, 1.6 Hz, 1H), 7.81 (dd, *J* = 8.5, 1.0 Hz, 1H), 7.54 (ddd, *J* = 8.7, 7.2, 1.7 Hz, 1H), 7.20 (td, *J* = 7.6, 1.0 Hz, 1H), 5.10 (ddd, *J* = 46.9, 10.5, 4.7 Hz, 1H), 4.63 (td, *J* = 13.0, 4.7 Hz, 1H), 4.04 (ddd, *J* = 13.3, 10.6, 4.8 Hz, 1H), 1.57 (s, 9H). ^19^F-NMR (376 MHz, Chloroform-*d*) δ −198.77. ^13^C-NMR (101 MHz, Chloroform-*d*) δ 189.7 (C-F, ^2^*J*_CF_ = 14.1 Hz), 189.6 (C-F, ^2^*J*_CF_ = 14.1 Hz), 152.8, 144.1, 135.0, 128.1, 124.6, 123.8, 123.2, 88.1 (C-F, ^1^*J*_CF_ = 190.9 Hz), 86.2 (C-F, ^1^*J*_CF_ = 190.9 Hz), 83.2, 48.8 (C-F, ^2^*J*_CF_ = 27.3 Hz), 48.5 (C-F, ^2^*J*_CF_ = 27.3 Hz), 28.3. HRMS (APCI): *m*/*z* [M + H]^+^ calcd. for C_14_H_17_FNO_3_ 266.1187, found 266.1186.

*tert*-Butyl 3-fluoro-6-methyl-4-oxo-3,4-dihydroquinoline-1(2*H*)-carboxylate (**2b**). 550 mg of *tert*-butyl 6-methyl-4-oxo-3,4-dihydroquinoline-1(2*H*)-carboxylate (2.10 mmol, 1.0 equiv) were used following the described procedure. Purification via flash column chromatography on silica gel (95:5 petroleum ether/ethyl acetate) yielded 470 mg of **2b** as a white solid (80%). m.p. 122–126 °C. ^1^H-NMR (400 MHz, Chloroform-*d*) δ 7.75 (s, 1H), 7.62 (d, *J* = 8.5 Hz, 1H), 7.28 (dd, *J* = 9.8, 3.4 Hz, 1H), 5.01 (ddd, *J* = 46.9, 10.6, 4.7 Hz, 1H), 4.55 (tdd, *J* = 13.2, 4.7, 0.9 Hz, 1H), 3.93 (ddd, *J* = 13.5, 10.6, 4.8 Hz, 1H), 2.28 (s, 3H), 1.49 (s, 9H). ^19^F-NMR (376 MHz, Chloroform-*d*) δ –198.75. ^13^C-NMR (101 MHz, Chloroform-*d*) δ 189.9 (C-F, ^2^*J*_CF_ = 14.1 Hz), 189.8 (C-F, ^2^*J*_CF_ = 14.1 Hz), 152.9, 141.8, 136.0, 134.5, 127.8, 123.7, 123.0, 88.2 (C-F, ^1^*J*_CF_ = 190.9 Hz), 86.3 (C-F, ^1^*J*_CF_ = 190.9 Hz), 83.0, 48.8 (C-F, ^2^*J*_CF_ = 27.3 Hz), 48.5 (C-F, ^2^*J*_CF_ = 27.3 Hz), 28.4, 20.7. HRMS (APCI): *m*/*z* [M − Boc + H]^+^ calcd. for C_10_H_11_FNO 180.0825, found 180.0819.

*tert*-Butyl 3-fluoro-6-methoxy-4-oxo-3,4-dihydroquinoline-1(2*H*)-carboxylate (**2c**). 700 mg of *tert*-butyl 6-methoxy-4-oxo-3,4-dihydroquinoline-1(2*H*)-carboxylate (2.52 mmol, 1.0 equiv) were used following the described procedure. Purification via flash column chromatography on silica gel (95:5 to 85:15 petroleum ether/ethyl acetate) yielded 487 mg of **2c** as a pale-yellow solid (70%). m.p. 136–140 °C. ^1^H-NMR (400 MHz, Chloroform-*d*) δ 7.69 (dd, *J* = 9.7, 2.9 Hz, 1H), 7.43 (dd, *J* = 7.0, 4.2 Hz, 1H), 7.11 (dt, *J* = 12.5, 8.2 Hz, 1H), 5.08 (ddd, *J* = 46.8, 17.6, 13.6 Hz, 1H), 4.60 (td, *J* = 13.2, 8.9 Hz, 1H), 4.01 (ddt, *J* = 15.3, 10.6, 3.2 Hz, 1H), 3.82 (s, 3H), 1.54 (s, 9H). ^19^F-NMR (376 MHz, Chloroform-*d*) δ –198.57. ^13^C-NMR (101 MHz, Chloroform-*d*) δ 189.7 (C-F, ^2^*J*_CF_ = 15.2 Hz), 189.5 (C-F, ^2^*J*_CF_ = 15.2 Hz), 156.5, 152.9, 137.9, 125.5, 124.0, 123.5, 108.9, 88.7 (C-F, ^1^*J*_CF_ = 190.9 Hz), 86.4 (C-F, ^1^*J*_CF_ = 190.9 Hz), 82.9, 55.8, 48.9 (C-F, ^2^*J*_CF_ = 27.3 Hz), 48.7 (C-F, ^2^*J*_CF_ = 27.3 Hz), 28.3. HRMS (APCI): *m*/*z* [M − Boc + H]^+^ calcd. for C_10_H_11_FNO_2_ 196.0774, found 196.0769.

*tert*-Butyl 3-fluoro-7-methoxy-4-oxo-3,4-dihydroquinoline-1(2*H*)-carboxylate (**2d**). 313 mg of *tert*-butyl 7-methoxy-4-oxo-3,4-dihydroquinoline-1(2*H*)-carboxylate (1.13 mmol, 1.0 equiv) were used following the described procedure. Purification via flash column chromatography on silica gel (95:5 to 85:15 petroleum ether/ethyl acetate) yielded 247 mg of **2d** as a pale-yellow solid (74%). m.p. 93–97 °C. ^1^H-NMR (400 MHz, Chloroform-*d*) δ 7.98 (dd, *J* = 9.0, 1.3 Hz, 1H), 7.37 (d, *J* = 2.4 Hz, 1H), 6.74 (dd, *J* = 8.9, 2.4 Hz, 1H), 5.04 (ddd, *J* = 47.0, 10.3, 4.6 Hz, 1H), 4.58 (td, *J* = 13.4, 4.6 Hz, 1H), 4.03 (ddd, *J* = 13.2, 10.3, 5.2 Hz, 1H), 3.88 (s, 3H), 1.58 (s, 9H). ^19^F-NMR (376 MHz, Chloroform-*d*) δ –199.06. ^13^C-NMR (101 MHz, Chloroform-*d*) δ 188.3 (C-F, ^2^*J*_CF_ = 15.2 Hz), 188.1 (C-F, ^2^*J*_CF_ = 15.2 Hz), 165.1, 152.7, 146.0, 130.2, 116.9, 112.0, 107.7, 87.8 (C-F, ^1^*J*_CF_ = 189.9 Hz), 85.9 (C-F, ^1^*J*_CF_ = 189.9 Hz), 83.2, 55.9, 48.9 (C-F, ^2^*J*_CF_ = 27.3 Hz), 48.7 (C-F, ^2^*J*_CF_ = 27.3 Hz), 28.4. HRMS (APCI): *m*/*z* [M + H]^+^ calcd. for C_15_H_19_FNO_4_ 296.1293, found 296.1295.

*tert*-Butyl 3-fluoro-6,7-dimethoxy-4-oxo-3,4-dihydroquinoline-1(2*H*)-carboxylate (**2e**). 330 mg of *tert*-butyl 6,7-dimethoxy-4-oxo-3,4-dihydroquinoline-1(2*H*)-carboxylate (1.07 mmol, 1.0 equiv) were used following the described procedure. Purification via flash column chromatography on silica gel (90:10 to 80:20 petroleum ether/ethyl acetate) yielded 257 mg of **2e** as a pale-yellow solid (74%). m.p. 168–172 °C. ^1^H-NMR (400 MHz, Chloroform-*d*) δ 7.46–7.36 (m, 2H), 5.05 (ddd, *J* = 47.0, 10.1, 4.5 Hz, 1H), 4.56 (ddd, *J* = 14.3, 13.2, 4.5 Hz, 1H), 4.06 (ddd, *J* = 13.3, 10.1, 5.4 Hz, 1H), 3.96 (s, 3H), 3.91 (s, 3H), 1.57 (s, 9H). ^19^F-NMR (376 MHz, Chloroform-*d*) δ −198.64. ^13^C-NMR (101 MHz, Chloroform-*d*) δ 188.3 (C-F, ^2^*J*_CF_ = 15.2 Hz), 188.1 (C-F, ^2^*J*_CF_ = 15.2 Hz), 154.8, 152.8, 146.6, 140.0, 116.2, 108.0, 106.3, 87.9 (C-F, ^1^*J*_CF_ = 188.9 Hz), 86.0 (C-F, ^1^*J*_CF_ = 188.9 Hz), 83.0, 56.4, 56.3, 49.4 (C-F, ^2^*J*_CF_ = 27.3 Hz), 49.1 (C-F, ^2^*J*_CF_ = 27.3 Hz), 28.4. HRMS (APCI): *m*/*z* [M + H]^+^ calcd. for C_16_H_21_FNO_5_ 326.1398, found 326.1399.

*tert*-Butyl 3,6-difluoro-4-oxo-3,4-dihydroquinoline-1(2*H*)-carboxylate (**2f**). 500 mg of *tert*-butyl 6-fluoro-4-oxo-3,4-dihydroquinoline-1(2*H*)-carboxylate (1.88 mmol, 1.0 equiv) were used following the described procedure. Purification via flash column chromatography on silica gel (95:5 to 85:15 petroleum ether/ethyl acetate) yielded 474 mg of **2f** as a pale-yellow solid (86%). m.p. 89–93 °C. ^1^H-NMR (400 MHz, Chloroform-*d*) δ 7.81 (ddd, *J* = 9.7, 4.5, 2.3 Hz, 1H), 7.65 (dt, *J* = 8.3, 4.3 Hz, 1H), 7.31–7.20 (m, 1H), 5.10 (ddd, *J* = 46.7, 10.0, 2.7 Hz, 1H), 4.59 (td, *J* = 13.4, 3.8 Hz, 1H), 4.06 (ddd, *J* = 12.1, 5.1, 2.5 Hz, 1H), 1.55 (s, 9H). m.p. 89–93 °C. ^19^F-NMR (376 MHz, Chloroform-*d*) δ −116.65, −198.88. ^13^C-NMR (101 MHz, Chloroform-*d*) δ 188.8 (C-F, ^2^*J*_CF_ = 15.2 Hz), 188.6 (C-F, ^2^*J*_CF_ = 15.2 Hz), 160.5 (C-F, ^1^*J*_CF_ = 248.5 Hz), 158.1 (C-F, ^1^*J*_CF_ = 248.5 Hz), 152.7, 140.4, 126.1 (C-F, ^3^*J*_CF_ = 7.1 Hz), 126.1 (C-F, ^3^*J*_CF_ = 7.1 Hz), 124.6 (C-F, ^3^*J*_CF_ = 6.1 Hz), 124.6 (C-F, ^3^*J*_CF_ = 6.1 Hz), 122.5 (C-F, ^2^*J*_CF_ = 23.2 Hz), 122.3 (C-F, ^2^*J*_CF_ = 23.2 Hz), 113.5 (C-F, ^2^*J*_CF_ = 23.2 Hz), 113.3 (C-F, ^2^*J*_CF_ = 23.2 Hz), 88.0 (C-F, ^1^*J*_CF_ = 191.9 Hz), 86.1 (C-F, ^1^*J*_CF_ = 191.9 Hz), 83.4, 48.8 (C-F, ^2^*J*_CF_ = 26.3 Hz), 48.5 (C-F, ^2^*J*_CF_ = 26.3 Hz), 28.3. HRMS (APCI): *m*/*z* [M − Boc + H]^+^ calcd. for C_9_H_8_F_2_NO 184.0574, found 184.0569.

*tert*-Butyl 3-fluoro-4-oxo-6-(trifluoromethyl)-3,4-dihydroquinoline-1(2*H*)-carboxylate (**2g**). 700 mg of *tert*-butyl 4-oxo-6-(trifluoromethyl)-3,4-dihydroquinoline-1(2*H*)-carboxylate (2.22 mmol, 1.0 equiv) were used following the described procedure. Purification via flash column chromatography on silica gel (95:5 to 85:15 petroleum ether/ethyl acetate) yielded 335 mg of **2g** as a white solid (45%). m.p. 101–105 °C. ^1^H-NMR (400 MHz, Chloroform-*d*) δ 8.29 (d, *J* = 2.3 Hz, 1H), 8.03 (dd, *J* = 8.9, 0.6 Hz, 1H), 7.75 (dd, *J* = 7.1, 2.8 Hz, 1H), 5.13 (ddd, *J* = 46.7, 10.3, 4.6 Hz, 1H), 4.62 (ddd, *J* = 14.3, 13.6, 4.6 Hz, 1H), 4.12 (ddd, *J* = 13.5, 10.3, 5.1 Hz, 1H), 1.58 (s, 9H). ^19^F-NMR (376 MHz, Chloroform-*d*) δ −62.76, −199.13. ^13^C-NMR (101 MHz, Chloroform-*d*) δ 188.4 (C-F, ^2^*J*_CF_ = 15.2 Hz), 188.2 (C-F, ^2^*J*_CF_ = 15.2 Hz), 152.3, 146.6, 131.3 (C-F, ^3^*J*_CF_ = 4.0 Hz), 131.2 (C-F, ^3^*J*_CF_ = 4.0 Hz), 127.6 (C-F, ^1^*J*_CF3_ = 272.7 Hz), 127.2 (C-F, ^2^*J*_CF3_ = 33.3 Hz), 126.9 (C-F, ^2^*J*_CF3_ = 33.3 Hz), 126.6 (C-F, ^2^*J*_CF3_ = 33.3 Hz), 126.2 (C-F, ^2^*J*_CF3_ = 33.3 Hz), 125.6, 124.9 (C-F, ^1^*J*_CF3_ = 272.7 Hz), 124.2, 122.7, 122.2 (C-F, ^1^*J*_CF3_ = 272.7 Hz), 119.5 (C-F, ^1^*J*_CF3_ = 272.7 Hz), 87.6 (C-F, ^1^*J*_CF_ = 190.9 Hz), 85.7 (C-F, ^1^*J*_CF_ = 190.9 Hz), 84.1, 48.5 (C-F, ^2^*J*_CF_ = 27.3 Hz), 48.3 (C-F, ^2^*J*_CF_ = 27.3 Hz), 28.3. HRMS (APCI): *m*/*z* [M − Boc + H]^+^ calcd. for C_10_H_8_F_4_NO 234.0542, found 234.0537.

*tert*-Butyl 6-chloro-3-fluoro-4-oxo-3,4-dihydroquinoline-1(2*H*)-carboxylate (**2h**). 260 mg of *tert*-butyl 4-oxo-6-chloro-3,4-dihydroquinoline-1(2*H*)-carboxylate (0.92 mmol, 1.0 equiv) were used following the described procedure. Purification via flash column chromatography on silica gel (95:5 petroleum ether/ethyl acetate) yielded 107 mg of **2h** as a yellow solid (39%). m.p. 117–121 °C. ^1^H-NMR (400 MHz, Chloroform-*d*) δ 7.97 (d, *J* = 2.6 Hz, 1H), 7.81 (d, *J* = 9.0 Hz, 1H), 7.48 (dd, *J* = 9.0, 2.6 Hz, 1H), 5.09 (ddd, *J* = 46.8, 10.4, 4.7 Hz, 1H), 4.60 (td, *J* = 13.5, 4.6 Hz, 1H), 4.06 (ddd, *J* = 13.4, 10.4, 4.9 Hz, 1H), 1.56 (s, 9H). ^19^F-NMR (376 MHz, Chloroform-*d*) δ −198.92. ^13^C-NMR (101 MHz, Chloroform-*d*) δ 188.5 (C-F, ^2^*J*_CF_ = 15.2 Hz), 188.4 (C-F, ^2^*J*_CF_ = 15.2 Hz), 152.51, 142.5, 134.8, 130.4, 127.4, 125.3, 124.1, 87.8 (C-F, ^1^*J*_CF_ = 191.9 Hz), 85.9 (C-F, ^1^*J*_CF_ = 191.9 Hz), 83.6, 48.6 (C-F, ^2^*J*_CF_ = 26.3 Hz), 48.4 (C-F, ^2^*J*_CF_ = 26.3 Hz), 28.3. HRMS (APCI): *m*/*z* [M + H]^+^ calcd. for C_14_H_16_ClFNO_3_ 300.0797, found 300.0799.

*tert*-Butyl 6-bromo-3-fluoro-4-oxo-3,4-dihydroquinoline-1(2*H*)-carboxylate (**2i**). 380 mg of *tert*-butyl 4-oxo-6-bromo-3,4-dihydroquinoline-1(2*H*)-carboxylate (1.16 mmol, 1.0 equiv) were used following the described procedure. Purification via flash column chromatography on silica gel (95:5 petroleum ether/ethyl acetate) yielded 165 mg of **2i** as a white solid (41%). m.p. 111–114 °C. ^1^H-NMR (400 MHz, Chloroform-*d*) δ 8.12 (d, *J* = 2.4 Hz, 1H), 7.75 (d, *J* = 9.0 Hz, 1H), 7.62 (dd, *J* = 9.0, 2.5 Hz, 1H), 5.09 (ddd, *J* = 46.8, 10.4, 4.7 Hz, 1H), 4.59 (td, *J* = 13.5, 4.6 Hz, 1H), 4.05 (ddd, *J* = 13.4, 10.4, 5.0 Hz, 1H), 1.56 (s, 9H). ^19^F-NMR (376 MHz, Chloroform-*d*) δ −198.90. ^13^C-NMR (101 MHz, Chloroform-*d*) δ 188.4 (C-F, ^2^*J*_CF_ = 15.2 Hz), 188.3 (C-F, ^2^*J*_CF_ = 15.2 Hz), 152.4, 143.0, 137.7, 130.5, 125.5, 124.4, 117.8, 87.8 (C-F, ^1^*J*_CF_ = 191.9 Hz), 85.9 (C-F, ^1^*J*_CF_ = 191.9 Hz), 83.7, 48.6 (C-F, ^2^*J*_CF_ = 27.3 Hz), 48.3 (C-F, ^2^*J*_CF_ = 27.3 Hz), 28.3. HRMS (APCI): *m*/*z* [M − Boc + H]^+^ calcd. for C_9_H_8_BrFNO 243.9773, found 243.9768.

*tert*-Butyl 3-fluoro-6-iodo-4-oxo-3,4-dihydroquinoline-1(2*H*)-carboxylate (**2j**). 339 mg of *tert*-butyl 4-oxo-6-iodo-3,4-dihydroquinoline-1(2*H*)-carboxylate (0.91 mmol, 1.0 equiv) were used following the described procedure. Purification via flash column chromatography on silica gel (97:3 petroleum ether/ethyl acetate) yielded 160 mg of **2j** as a white solid (45%). m.p. 113–117 °C. ^1^H-NMR (400 MHz, Chloroform-*d*) δ 8.31 (d, *J* = 2.2 Hz, 1H), 7.80 (dd, *J* = 8.9, 2.3 Hz, 1H), 7.61 (d, *J* = 8.9 Hz, 1H), 5.08 (ddd, *J* = 46.8, 10.4, 4.7 Hz, 1H), 4.59 (td, *J* = 13.5, 4.7 Hz, 1H), 4.04 (ddd, *J* = 13.4, 10.4, 5.0 Hz, 1H), 1.56 (s, 9H). ^19^F-NMR (376 MHz, Chloroform-*d*) δ −198.84. ^13^C-NMR (101 MHz, Chloroform-*d*) δ 188.3 (C-F, ^2^*J*_CF_ = 15.2 Hz), 188.2 (C-F, ^2^*J*_CF_ = 15.2 Hz), 152.4, 143.6, 143.4, 136.7, 125.6, 124.5, 88.0, 87.6 (C-F, ^1^*J*_CF_ = 190.9 Hz), 85.8 (C-F, ^1^*J*_CF_ = 190.9 Hz), 83.7, 48.5 (C-F, ^2^*J*_CF_ = 24.2 Hz), 48.3 (C-F, ^2^*J*_CF_ = 24.2 Hz), 28.3. HRMS (APCI): *m*/*z* [M + H]^+^ calcd. for C_14_H_16_FINO_3_ 392.0153, found 392.0155.

### 3.3. General Procedure for the ATH of tert-Butyl 3-fluoro-4-oxo-3,4-dihydroquinoline-1(2H)-carboxylates 

In a round-bottom tube charged with the corresponding 3-fluoro-4-oxo-3,4-dihydroquinoline-1(2*H*)-carboxylate (1.0 equiv) set under argon, the necessary volume of a solution of (*R,R*)-A in acetonitrile (1.0 mL of a 1.24 mg/mL solution; 0.005 equiv) was added. The mixture was stirred for one minute before adding, by syringe, the necessary volume of formic acid/triethylamine (1:1) mixture (6.0 equiv). The reaction mixture was stirred at 40 °C for the time needed and then quenched with 3 mL of saturated NaHCO_3_ aqueous solution. The media was extracted with CH_2_Cl_2_ (2 × 4 mL) and the organic layers were dried over MgSO_4_, filtered, and concentrated under vacuum. The diastereoisomeric ratio was determined by ^1^H-NMR analysis of the crude product. The product was purified with a flash column chromatography on silica gel (petroleum ether/EtOAc) and the enantiomeric excess was determined by SFC analysis (CHIRALPAK IE or IF column) (see Supplementary Materials for NMR and SFC spectra).

*tert*-Butyl (3*R*,4*S*)-3-fluoro-4-hydroxy-3,4-dihydroquinoline-1(2*H*)-carboxylate (**3a**). 100 mg of *tert*-butyl 3-fluoro-4-oxo-3,4-dihydroquinoline-1(2*H*)-carboxylate (**2a**) (0.38 mmol; 1.0 equiv) were used according to the described procedure (reaction time of 3 h). The product was purified by flash column chromatography on silica gel (petroleum ether/ethyl acetate 75:25) and yielded 93 mg of a beige/white solid (93%). m.p. 142–144 °C dr (*cis*/*trans*) = 98:2, ee*_cis_* = > 99%. [*α*]_D_^30^ = −5 (*c* 1.0, CHCl_3_). ^1^H-NMR (400 MHz, Chloroform-*d*) δ 7.70 (d, *J* = 8.3 Hz, 1H), 7.51 (dt, *J* = 7.6, 1.4 Hz, 1H), 7.34–7.24 (m, 1H), 7.14 (td, *J* = 7.5, 1.2 Hz, 1H), 5.05 (ddt, *J* = 50.8, 4.8, 3.5 Hz, 1H), 4.78 (dd, *J* = 21.5, 5.9 Hz, 1H), 4.10 (ddd, *J* = 14.9, 14.2, 4.8 Hz, 1H), 3.94 (ddd, *J* = 29.9, 14.2, 3.7 Hz, 1H), 2.38 (d, *J* = 8.3 Hz, 1H), 1.53 (s, 9H). ^19^F-NMR (376 MHz, Chloroform-*d*) δ −200.34. ^13^C-NMR (101 MHz, Chloroform-*d*) δ 153.8, 136.8, 128.2, 127.9, 127.3, 124.3, 123.5, 89.6 (C-F, ^1^*J*_CF_ = 176.8 Hz), 87.9 (C-F, ^1^*J*_CF_ = 176.8 Hz), 81.9, 68.0 (C-F, ^2^*J*_CF_ = 19.2 Hz), 67.8 (C-F, ^2^*J*_CF_ = 19.2 Hz), 46.3 (C-F, ^2^*J*_CF_ = 23.2 Hz), 46.1 (C-F, ^2^*J*_CF_ = 23.2 Hz), 28.4. HRMS (APCI): *m*/*z* [M + H]^+^ calcd. for C_14_H_19_FNO_3_ 268.1343, found 268.1345. SFC: Chiralpak IE, *sc*CO_2_/EtOH 96/4, 2.0 mL/min, P = 100 bar, λ = 215 nm, t_R_ [*trans*] = 11.04 min, t_R_ [*trans*] = 12.85 min, t_R_ [*cis*-(3*R*,4S)] = 14.54 min (major), t_R_ [*cis*-(3*S*,4*R*)] = 15.43 min.

*tert*-Butyl (3*R*,4*S*)-3-fluoro-4-hydroxy-6-methyl-3,4-dihydroquinoline-1(2*H*)-carboxylate (**3b**). 100 mg of *tert*-butyl 3-fluoro-6-methyl-4-oxo-3,4-dihydroquinoline-1(2*H*)-carboxylate (**2b**) (0.36 mmol; 1.0 equiv) were used according to the described procedure (reaction time of 3 h). The product was purified by flash column chromatography on silica gel (petroleum ether/ethyl acetate 75:25) and yielded 91 mg of a colourless oil (91%). dr (*cis*/*trans*) = 98:2, ee*_cis_* = > 99%. [*α*]_D_^22^ = −8.4 (*c* 1.0, CHCl_3_). ^1^H-NMR (400 MHz, Chloroform-*d*) δ 7.56 (d, *J* = 8.4 Hz, 1H), 7.34–7.28 (m, 1H), 7.08 (dd, *J* = 8.4, 2.1 Hz, 1H), 5.00 (dq, *J* = 50.9, 4.2 Hz, 1H), 4.72 (dd, *J* = 22.0, 7.0 Hz, 1H), 4.10–3.84 (m, 2H), 2.53 (s, 1H), 2.32 (s, 3H), 1.52 (s, 9H). ^19^F-NMR (376 MHz, Chloroform-*d*) δ −200.17. ^13^C-NMR (101 MHz, Chloroform-*d*) δ 153.8, 134.2, 133.9, 128.9, 127.6, 123.4, 89.7, 88.0 (C-F, ^1^*J*_CF_ = 176.8 Hz), 81.7 (C-F, ^1^*J*_CF_ = 176.8 Hz), 67.9 (C-F, ^2^*J*_CF_ = 20.2 Hz), 67.7 (C-F, ^2^*J*_CF_ = 20.2 Hz), 46.2 (C-F, ^2^*J*_CF_ = 23.2 Hz), 46.0 (C-F, ^2^*J*_CF_ = 23.2 Hz), 28.4, 20.9. HRMS (APCI): *m*/*z* [M + H]^+^ calcd. for C_15_H_21_FNO_3_ 282.1500, found 282.1502. SFC: Chiralpak IE, *sc*CO_2_/EtOH 97.5/2.5, 2.0 mL/min, P = 100 bar, λ = 215 nm, t_R_ [*trans*] = 27.71 min, t_R_ [*cis*-(3*R*,4S)] = 32.43 min (major), t_R_ [*cis*-(3*S*,4*R*)] = 37.48 min, t_R_ [*trans*] = 42.41 min.

*tert*-Butyl (3*R*,4*S*)-3-fluoro-4-hydroxy-6-methoxy-3,4-dihydroquinoline-1(2*H*)-carboxylate (**3c**). 100 mg of *tert*-butyl 3-fluoro-6-methoxy-4-oxo-3,4-dihydroquinoline-1(2*H*)-carboxylate (**2c**) (0.34 mmol; 1.0 equiv) were used according to the described procedure (reaction time of 5 h). The product was purified by flash column chromatography on silica gel (petroleum ether/ethyl acetate 75:25) and yielded 95 mg of a white/red solid (95%). m.p. 114–118 °C dr (*cis*/*trans*) = 98:2, ee*_cis_* = > 99%. [*α*]_D_^22^ = −13.3 (*c* 1.0, CHCl_3_). ^1^H-NMR (400 MHz, Chloroform-*d*) δ 7.57 (d, *J* = 9.1 Hz, 1H), 7.05 (dd, *J* = 3.0, 1.0 Hz, 1H), 6.83 (dd, *J* = 9.1, 3.0 Hz, 1H), 5.06 (dd, *J* = 51.1, 3.7 Hz, 1H), 4.74 (ddd, *J* = 22.7, 9.4, 3.2 Hz, 1H), 4.08 (ddd, *J* = 15.9, 14.4, 4.4 Hz, 1H), 3.92 (ddd, *J* = 31.4, 14.5, 3.7 Hz, 1H), 3.81 (s, 3H), 2.37 (d, *J* = 9.4 Hz, 1H), 1.52 (s, 9H). ^19^F-NMR (376 MHz, Chloroform-d) δ −200.32. ^13^C-NMR (101 MHz, Chloroform-*d*) δ 156.5, 153.9, 129.8, 129.4, 125.0, 114.3, 111.4, 89.9 (C-F, ^1^*J*_CF_ = 176.8 Hz), 88.1 (C-F, ^1^*J*_CF_ = 176.8 Hz), 81.6, 68.2 (C-F, ^2^*J*_CF_ = 20.2 Hz), 68.0 (C-F, ^2^*J*_CF_ = 20.2 Hz), 55.7, 46.6 (C-F, ^2^*J*_CF_ = 23.2 Hz), 46.3 (C-F, ^2^*J*_CF_ = 23.2 Hz), 28.4. HRMS (APCI): *m*/*z* [M + Na]^+^ calcd. for C_15_H_20_FNO_4_Na 320.1269, found 320.1273. SFC: Chiralpak IF, *sc*CO_2_/EtOH 95.5/45, 2.0 mL/min, P = 100 bar, λ = 254 nm, t_R_ [*trans*] = 15.71 min, t_R_ [*cis*-(3*S*,4*R*)] = 17.11 min, t_R_ [*trans*] = 18.69 min, t_R_ [*cis*-(3*R*,4S)] = 22.79 min (major)

*tert*-Butyl (3*R*,4*S*)-3-fluoro-4-hydroxy-7-methoxy-3,4-dihydroquinoline-1(2*H*)-carboxylate (**3d**). 100 mg of *tert*-butyl 3-fluoro-7-methoxy-4-oxo-3,4-dihydroquinoline-1(2*H*)-carboxylate (**2d**) (0.34 mmol; 1.0 equiv) were used according to the described procedure (reaction time of 5 h). The product was purified by flash column chromatography on silica gel (petroleum ether/ethyl acetate 75:25) and yielded 95 mg of a beige/white solid (95%). m.p. 92–96 °C dr(*cis*/*trans*) = 95:5, ee*_cis_* = >99%. [*α*]_D_^22^ = −9.2 (*c* 1.0, CHCl_3_). ^1^H-NMR (400 MHz, Chloroform-*d*) δ 7.42–7.31 (m, 2H), 6.70 (dt, *J* = 8.6, 2.3 Hz, 1H), 4.98 (dd, *J* = 50.0, 5.9 Hz, 1H), 4.76 (d, *J* = 19.1 Hz, 1H), 4.16–4.04 (m, 1H), 4.01–3.85 (m, 1H), 3.80 (s, 3H), 2.33 (s, 1H), 1.54 (s, 9H). ^19^F-NMR (376 MHz, Chloroform-*d*) δ −201.08. ^13^C-NMR (101 MHz, Chloroform-*d*) δ 159.7, 153.6, 138.0, 128.9, 119.8, 110.8, 108.6, 89.1 (C-F, ^1^*J*_CF_ = 177.8 Hz), 87.3 (C-F, ^1^*J*_CF_ = 177.8 Hz), 82.0, 67.6 (C-F, ^2^*J*_CF_ = 19.2 Hz), 67.4 (C-F, ^2^*J*_CF_ = 19.2 Hz), 55.5, 45.7 (C-F, ^2^*J*_CF_ = 25.3 Hz), 45.5 (C-F, ^2^*J*_CF_ = 25.3 Hz), 28.5. HRMS (APCI): *m*/*z* [M + NH_4_]^+^ calcd. for C_15_H_24_FN_2_O_4_ 315.1715, found 315.1718. SFC: Chiralpak IE, *sc*CO_2_/EtOH 95/5, 2.0 mL/min, P = 100 bar, λ = 215 nm, t_R_ [*trans*] = 11.87 min, t_R_ [*trans*] = 13.19 min, t_R_ [*cis*-(3*R*,4S)] = 16.63 min (major), t_R_ [*cis*-(3*S*,4*R*)] = 18.62 min.

*tert*-Butyl (3*R*,4*S*)-3-fluoro-4-hydroxy-6,7-dimethoxy-3,4-dihydroquinoline-1(2*H*)-carboxylate (**3e**). 100 mg of *tert*-butyl 3-fluoro-6,7-dimethoxy-4-oxo-3,4-dihydroquinoline-1(2*H*)-carboxylate (**2e**) (0.31 mmol; 1.0 equiv) were used according to the described procedure with 1.0 mol% of catalyst (*R,R*)-**D** (reaction time of 24 h). The product was purified by flash column chromatography on silica gel (petroleum ether/ethyl acetate 60:40) and yielded 90 mg of a colourless oil (90%). dr(*cis*/*trans*) = 94:6, ee*_cis_* = 99%. [*α*]_D_^22^ = −11.8 (*c* 1.0, CHCl_3_). ^1^H-NMR (400 MHz, Chloroform-*d*) δ 7.32 (s, 1H), 6.97 (d, *J* = 0.8 Hz, 1H), 4.99 (ddt, *J* = 50.2, 5.6, 3.4 Hz, 1H), 4.74 (dq, *J* = 19.9, 3.5 Hz, 1H), 4.20–4.11 (m, 1H), 3.94–3.80 (m, 7H), 2.38 (d, *J* = 8.4 Hz, 1H), 1.53 (s, 9H). ^19^F-NMR (376 MHz, Chloroform-d) δ −202.03. ^13^C-NMR (101 MHz, Chloroform-*d*) δ 153.8, 148.5, 146.2, 130.4, 119.6, 110.0, 107.5, 89.1 (C-F, ^1^*J*_CF_ = 177.8 Hz), 87.3 (C-F, ^1^*J*_CF_ = 177.8 Hz), 81.7, 67.7 (C-F, ^2^*J*_CF_ = 20.2 Hz), 67.5 (C-F, ^2^*J*_CF_ = 20.2 Hz), 56.2, 56.1, 46.0 (C-F, ^2^*J*_CF_ = 24.2 Hz), 45.8 (C-F, ^2^*J*_CF_ = 24.2 Hz), 28.5. HRMS (APCI): *m*/*z* [M + Na]^+^ calcd. for C_16_H_22_FNO_5_Na 350.1374, found 320.1377. SFC: Chiralpak IF, *sc*CO_2_/EtOH 95/5, 2.0 mL/min, P = 100 bar, λ = 215 nm, t_R_ [*trans*] = 14.75 min, t_R_ [*trans*] = 15.89 min, t_R_ [*cis*-(3*R*,4S)] = 17.45 min (major), t_R_ [*cis*-(3*S*,4*R*)] = 19.07 min.

*tert*-Butyl (3*R*,4*S*)-3,6-difluoro-4-hydroxy-3,4-dihydroquinoline-1(2*H*)-carboxylate (**3f**). 100 mg of *tert*-butyl 3,6-difluoro-4-oxo-3,4-dihydroquinoline-1(2*H*)-carboxylate (**2f**) (0.35 mmol; 1.0 equiv) were used according to the described procedure (reaction time of 24 h). The product was purified by flash column chromatography on silica gel (petroleum ether/ethyl acetate 75:25) and yielded 70 mg of a white solid (70%). m.p. 78–82 °C dr(*cis*/*trans*) = 98:2, ee*_cis_* = > 99%. [*α*]_D_^22^ = −11.7 (*c* 1.0, CHCl_3_). ^1^H-NMR (400 MHz, Chloroform-*d*) δ 7.64 (dd, *J* = 9.3, 5.1 Hz, 1H), 7.23 (ddd, *J* = 8.8, 3.0, 1.0 Hz, 1H), 6.97 (td, *J* = 8.9, 8.1, 3.1 Hz, 1H), 5.08 (dq, *J* = 51.2, 3.6 Hz, 1H), 4.71 (ddd, *J* = 23.6, 9.5, 3.1 Hz, 1H), 4.09 (ddd, *J* = 16.6, 14.6, 3.9 Hz, 1H), 3.91 (ddd, *J* = 33.1, 14.6, 3.6 Hz, 1H), 2.48 (dd, *J* = 9.7, 2.0 Hz, 1H), 1.52 (s, 9H). ^19^F-NMR (376 MHz, Chloroform-*d*) δ −118.05, −200.38. ^13^C-NMR (101 MHz, Chloroform-*d*) δ 160.8 (C-F, ^1^*J*_CF_ = 244.4 Hz), 158.4 (C-F, ^1^*J*_CF_ = 244.4 Hz), 153.7, 132.6 (C-F, ^4^*J*_CF_ = 2.0 Hz), 132.6 (C-F, ^4^*J*_CF_ = 2.0 Hz), 130.3 6 (C-F, ^3^*J*_CF_ = 6.1 Hz), 130.3 (C-F, ^3^*J*_CF_ = 6.1 Hz), 125.4 (C-F, ^3^*J*_CF_ = 7.1 Hz), 125.3 (C-F, ^3^*J*_CF_ = 7.1 Hz), 115.1 (C-F, ^2^*J*_CF_ = 22.2 Hz), 114.9 (C-F, ^2^*J*_CF_ = 22.2 Hz), 113.5 (C-F, ^2^*J*_CF_ = 22.2 Hz), 113.3 (C-F, ^2^*J*_CF_ = 22.2 Hz), 89.7 (C-F, ^1^*J*_CF_ = 176.8 Hz), 87.9 (C-F, ^1^*J*_CF_ = 176.8 Hz), 82.1, 67.9 (C-F, ^2^*J*_CF_ = 20.2 Hz), 67.7 (C-F, ^2^*J*_CF_ = 20.2 Hz), 46.8 (C-F, ^2^*J*_CF_ = 23.2 Hz), 46.6 (C-F, ^2^*J*_CF_ = 23.2 Hz), 28.4. HRMS (APCI): *m*/*z* [M + H]^+^ calcd. for C_14_H_18_F_2_NO_3_ 286.1249, found 282.1248. SFC: Chiralpak IE, *sc*CO_2_/MeOH 94/6, 2.0 mL/min, P = 100 bar, λ = 215 nm, t_R_ [*trans*] = 4.50 min, t_R_ [*trans*] = 4.96 min, t_R_ [*cis*-(3*S*,4*R*)] = 6.44 min, t_R_ [*cis*-(3*R*,4S)] = 7.00 min (major).

*tert*-Butyl (3*R*,4*S*)-3-fluoro-4-hydroxy-6-(trifluoromethyl)-3,4-dihydroquinoline-1(2*H*)-carboxylate (**3g**). 100 mg of *tert*-butyl 3-fluoro-6-trifluoromethyl-4-oxo-3,4-dihydroquinoline-1(2*H*)-carboxylate (**2g**) (0.30 mmol; 1.0 equiv) were used according to the described procedure (reaction time of 3 h). The product was purified by flash column chromatography on silica gel (petroleum ether/ethyl acetate 75:25) and yielded 97 mg of a beige/white solid (97%). m.p. 152–156 °C dr (*cis*/*trans*) = 98:2, ee*_cis_* = > 99%. [*α*]_D_^22^ = −0.9 (*c* 1.0, CHCl_3_). ^1^H-NMR (400 MHz, Chloroform-*d*) δ 7.88 (d, *J* = 8.7 Hz, 1H), 7.81 (d, *J* = 2.2 Hz, 1H), 7.51 (ddt, *J* = 8.7, 2.2, 0.7 Hz, 1H), 5.10 (dddd, *J* = 50.9, 7.7, 3.7, 1.4 Hz, 1H), 4.79 (dd, *J* = 23.1, 6.9 Hz, 1H), 4.17 (tdd, *J* = 15.5, 4.1, 1.1 Hz, 1H), 3.91 (dd, *J* = 33.7, 3.6 Hz, 1H), 2.45 (s, 1H), 1.54 (s, 9H). ^19^F-NMR (376 MHz, Chloroform-*d*) δ −62.20, −200.84. ^13^C-NMR (101 MHz, Chloroform-*d*) δ 153.4, 139.7, 128.3 (C-F, ^1^*J*_CF3_ = 272.7 Hz), 128.2, 128.1, 126.5 (C-F, ^2^*J*_CF3_ = 33.3 Hz), 126.2 (C-F, ^2^*J*_CF3_ = 33.3 Hz), 125.9 (C-F, ^2^*J*_CF3_ = 33.3 Hz), 125.6 (C-F, ^1^*J*_CF3_ = 272.7 Hz + C-F, ^2^*J*_CF3_ = 33.3 Hz), 125.1 (C-F, ^3^*J*_CF3_ = 4.0 Hz), 125.1 (C-F, ^3^*J*_CF3_ = 4.0 Hz), 124.4 (C-F, ^3^*J*_CF3_ = 4.0 Hz), 124.4 (C-F, ^3^*J*_CF3_ = 4.0 Hz), 123.4, 122.9 (C-F, ^1^*J*_CF3_ = 272.7 Hz), 120.2 (C-F, ^1^*J*_CF3_ = 272.7 Hz), 89.1 (C-F, ^1^*J*_CF_ = 176.8 Hz), 87.4 (C-F, ^1^*J*_CF_ = 176.8 Hz), 82.7, 67.7 (C-F, ^2^*J*_CF_ = 20.2 Hz), 67.5 (C-F, ^2^*J*_CF_ = 20.2 Hz), 46.8 (C-F, ^2^*J*_CF_ = 22.2 Hz), 46.6 (C-F, ^2^*J*_CF_ = 22.2 Hz), 28.3. HRMS (APCI): *m*/*z* [M + NH_4_]^+^ calcd. for C_15_H_21_FN_2_O3 353.1483, found 353.1483. SFC: Chiralpak IF, *sc*CO_2_/EtOH 96/4, 2.0 mL/min, P = 100 bar, λ = 254 nm, t_R_ [*cis*-(3*R*,4S)] = 5.68 min (major), t_R_ [*cis*-(3*S*,4*R*)] = 6.84 min, t_R_ [*trans*] = 8.54 min.

*tert*-Butyl (3*R*,4*S*)-6-chloro-3-fluoro-4-hydroxy-3,4-dihydroquinoline-1(2*H*)-carboxylate (**3h**). 60 mg of *tert*-butyl 3-fluoro-6-chloro-4-oxo-3,4-dihydroquinoline-1(2*H*)-carboxylate (**2h**) (0.20 mmol; 1.0 equiv) were used according to the described procedure (reaction time of 3 h). The product was purified by flash column chromatography on silica gel (petroleum ether/ethyl acetate 75:25) and yielded 97 mg of a beige/white solid (96%). m.p. 113–116 °C dr (*cis*/*trans*) = 99:1, ee*_cis_* = > 99%. [*α*]_D_^22^ = −12.8 (*c* 1.0, CHCl_3_). ^1^H-NMR (400 MHz, Chloroform-*d*) δ 7.66 (d, *J* = 8.9 Hz, 1H), 7.51 (dd, *J* = 2.6, 1.1 Hz, 1H), 7.22 (d, *J* = 9.2 Hz, 1H), 5.07 (dt, *J* = 50.8, 3.9 Hz, 1H), 4.78–4.67 (m, 1H), 4.17–4.05 (m, 1H), 3.99–3.81 (m, 1H), 2.44 (br, 1H), 1.52 (s, 9H). ^19^F-NMR (376 MHz, CDCl_3_) δ −200.57. ^13^C-NMR (101 MHz, Chloroform-*d*) δ 153.5, 135.2, 129.6, 128.2, 126.9, 124.8, 89.4 (C-F, ^1^*J*_CF_ = 176.8 Hz), 87.7 (C-F, ^1^*J*_CF_ = 176.8 Hz), 82.3, 67.7 (C-F, ^2^*J*_CF_ = 20.2 Hz), 67.5 (C-F, ^2^*J*_CF_ = 20.2 Hz), 46.7 (C-F, ^2^*J*_CF_ = 23.2 Hz), 46.5 (C-F, ^2^*J*_CF_ = 23.2 Hz), 28.4. HRMS (APCI): *m*/*z* [M + H]^+^ calcd. for C_14_H_18_ClFNO_3_ 302.0954, found 302.0955. SFC: Chiralpak IE, *sc*CO_2_/MeOH 94/6, 2.0 mL/min, P = 100 bar, λ = 254 nm, t_R_ [*trans*] = 6.95 min, t_R_ [*trans*] = 7.49 min, t_R_ [*cis*-(3*S*,4*R*)] = 10.31 min, t_R_ [*cis*-(3*R*,4S)] = 11.09 min (major).

*tert*-Butyl (3*R*,4*S*)-6-bromo-3-fluoro-4-hydroxy-3,4-dihydroquinoline-1(2*H*)-carboxylate (**3i**). 80 mg of *tert*-butyl 3-fluoro-6-bromo-4-oxo-3,4-dihydroquinoline-1(2*H*)-carboxylate (**2i**) (0.24 mmol; 1.0 equiv) were used according to the described procedure (reaction time of 3 h). The product was purified by flash column chromatography on silica gel (petroleum ether/ethyl acetate 75:25) and yielded 97 mg of a beige/white solid (95%). m.p. 128–131 °C dr (*cis*/*trans*) = 98:2, ee*_cis_* = > 99%. [*α*]_D_^22^ = −15.6 (*c* 1.0, CHCl_3_). ^1^H-NMR (400 MHz, Chloroform-*d*) δ 7.68–7.65 (m, 1H), 7.59 (d, *J* = 8.9 Hz, 1H), 7.37 (dd, *J* = 8.9, 2.4 Hz, 1H), 5.05 (dq, *J* = 51.0, 3.6 Hz, 1H), 4.71 (ddd, *J* = 23.2, 9.3, 3.1 Hz, 1H), 4.10 (ddd, *J* = 15.7, 14.4, 4.1 Hz, 1H), 3.88 (ddd, *J* = 33.1, 14.5, 3.5 Hz, 1H), 2.52 (d, *J* = 9.4 Hz, 1H), 1.52 (s, 9H). ^19^F-NMR (376 MHz, Chloroform-*d*) δ −200.49. ^13^C-NMR (101 MHz, Chloroform-*d*) δ 153.5, 135.7, 131.1, 130.0, 129.9, 125.1, 117.2, 89.4 (C-F, ^1^*J*_CF_ = 176.8 Hz), 87.6 (C-F, ^1^*J*_CF_ = 176.8 Hz), 82.3, 67.7 (C-F, ^2^*J*_CF_ = 20.2 Hz), 67.5 (C-F, ^2^*J*_CF_ = 20.2 Hz), 46.7 (C-F, ^2^*J*_CF_ = 23.2 Hz), 46.4 (C-F, ^2^*J*_CF_ = 23.2 Hz), 28.4. HRMS (APCI): *m*/*z* [M + NH_4_]^+^ calcd. for C_14_H_21_BrFN_2_O_3_ 363.0714, found 363.0714. SFC: Chiralpak IE, *sc*CO_2_/MeOH 93/7, 2.0 mL/min, P = 100 bar, λ = 254 nm, t_R_ [*trans*] = 7.72 min, t_R_ [*cis*-(3*S*,4*R*)] = 11.07 min, t_R_ [*cis*-(3*R*,4S)] = 11.96 min (major).

*tert*-Butyl (3*R*,4*S*)-3-fluoro-4-hydroxy-6-iodo-3,4-dihydroquinoline-1(2*H*)-carboxylate (**3j**). 80 mg of *tert*-butyl 3-fluoro-6-iodo-4-oxo-3,4-dihydroquinoline-1(2*H*)-carboxylate (**2j**) (0.20 mmol; 1.0 equiv) were used according to the described procedure (reaction time of 3 h). The product was purified by flash column chromatography on silica gel (petroleum ether/ethyl acetate 75:25) and yielded 97 mg of a beige/white solid (94%). m.p. 125–128 °C dr (*cis*/*trans*) = 98:2, ee*_cis_* = > 99%. [*α*]_D_^22^ = −19.6 (*c* 1.0, CHCl_3_). ^1^H-NMR (400 MHz, Chloroform-*d*) δ 7.84 (d, *J* = 0.8 Hz, 1H), 7.56 (dt, *J* = 8.7, 1.6 Hz, 1H), 7.47 (d, *J* = 8.8 Hz, 1H), 5.04 (dt, *J* = 50.9, 3.7 Hz, 1H), 4.71 (dd, *J* = 23.3, 8.8 Hz, 1H), 4.10 (ddt, *J* = 18.6, 15.1, 3.6 Hz, 1H), 3.97–3.78 (m, 1H), 2.46 (s, 1H), 1.52 (s, 9H). ^19^F-NMR (376 MHz, Chloroform-*d*) δ −200.57. ^13^C-NMR (101 MHz, Chloroform-*d*) δ 153.4, 137.0, 136.6, 135.9, 130.1, 125.3, 89.3 (C-F, ^1^*J*_CF_ = 176.8 Hz), 87.8 (C-F, ^1^*J*_CF_ = 176.8 Hz), 87.5, 82.4, 67.5 (C-F, ^2^*J*_CF_ = 20.2 Hz), 67.3 (C-F, ^2^*J*_CF_ = 20.2 Hz), 46.6 (C-F, ^2^*J*_CF_ = 23.2 Hz), 46.3 (C-F, ^2^*J*_CF_ = 23.2 Hz), 28.4. HRMS (APCI): *m*/*z* [M + NH_4_]^+^ calcd. for C_14_H_21_FIN_2_O_3_ 411.0575, found 411.0577. SFC: Chiralpak IE, *sc*CO_2_/MeOH 93/7, 2.0 mL/min, P = 100 bar, λ = 254 nm, t_R_ [*trans*] = 10.92 min, t_R_ [*trans*] = 11.41 min, t_R_ [*cis*-(3*S*,4*R*)] = 15.73 min, t_R_ [*cis*-(3*R*,4S)] = 17.25 min (major).

### 3.4. Gram-Scale Experiment 

In a 30 mL-round-bottom tube charged with *tert*-butyl 3-fluoro-4-oxo-3,4-dihydroquinoline-1(2*H*)-carboxylate (2a, 1.0 g, 3.8 mmol; 1.0 equiv) and the catalyst (*R,R*)-D (12.4 mg, 12 μmol; 0.005 equiv) set under argon were added 10 mL of acetonitrile. The mixture was stirred for one minute before adding by syringe a (1:1) mixture of formic acid and triethylamine (3.40 mL, 16 mmol; 6.0 equiv). The reaction mixture was stirred at 40 °C for 24 h. Then it was cooled down and quenched with 30 mL of NaHCO_3_ aqueous solution. The mixture was extracted with CH_2_Cl_2_ (2 × 40 mL) and the organic layers dried over MgSO_4_, filtered, and concentrated under vacuum. The diastereoisomeric ratio was determined by ^1^H-NMR analysis of the crude product. The product was purified with a flash column chromatography on silica gel (petroleum ether/EtOAc 70:30). 960 mg (96%) of (3*R*,4*R*)- 3-fluorochroman-4-ol (3a) was obtained as a white solid. dr (*cis*/*trans*) 97:3, ee*_cis_* = > 99%. ^1^H-NMR (400 MHz, Chloroform-*d*) δ 7.70 (d, *J* = 8.3 Hz, 1H), 7.51 (dt, *J* = 7.6, 1.4 Hz, 1H), 7.34–7.24 (m, 1H), 7.14 (td, *J* = 7.5, 1.2 Hz, 1H), 5.05 (ddt, *J* = 50.8, 4.8, 3.5 Hz, 1H), 4.78 (dd, *J* = 21.5, 5.9 Hz, 1H), 4.10 (ddd, *J* = 14.9, 14.2, 4.8 Hz, 1H), 3.94 (ddd, *J* = 29.9, 14.2, 3.7 Hz, 1H), 2.38 (d, *J* = 8.3 Hz, 1H), 1.53 (s, 9H). ^19^F-NMR (376 MHz, Chloroform-*d*) δ −200.34. ^13^C-NMR (101 MHz, Chloroform-*d*) δ 153.8, 136.8, 128.2, 127.9, 127.3, 124.3, 123.5, 89.6 (C-F, ^1^*J*_CF_ = 176.8 Hz), 87.9 (C-F, ^1^*J*_CF_ = 176.8 Hz), 81.9, 68.0 (C-F, ^2^*J*_CF_ = 19.2 Hz), 67.8 (C-F, ^2^*J*_CF_ = 19.2 Hz), 46.3 (C-F, ^2^*J*_CF_ = 23.2 Hz), 46.1 (C-F, ^2^*J*_CF_ = 23.2 Hz), 28.4. SFC: Chiralpak IE, *sc*CO_2_/EtOH 96/4, 2.0 mL/min, P = 100 bar, λ = 215 nm, t_R_ [*trans*] = 11.04 min, t_R_ [*trans*] = 12.85 min, t_R_ [*cis*-(3*R*,4S)] = 14.54 min (major), t_R_ [*cis*-(3*S*,4*R*)] = 15.43 min.

### 3.5. Sonogashira Coupling Reaction 

Tert-Butyl (3R,4S)-3-fluoro-4-hydroxy-6-(phenylethynyl)-3,4-dihydroquinoline-1(2H)-carboxylate (**4**). *Tert*-Butyl (3*R*,4*S*)-3-fluoro-4-hydroxy-6-iodo-3,4-dihydroquinoline-1(2*H*)-carboxylate (**3j**) (77 mg; 0.2 mmol; 1.0 equiv) was introduced into a 10 mL round-bottom tube at open air with 1 mL of a 1:1 CH_3_CN/Et_3_N mixture. Next, phenylacetylene was added (31 µL; 0.3 mmol; 1.5 equiv) followed by CuI (2.8 mg; 10 µmol; 0.05 equiv) and PdCl_2_(PPh_3_)_2_ (6.4 mg; 6 µmol; 0.03 equiv). The mixture was stirred for 30 min at room temperature. It was then quenched with 2 mL of NH_4_Cl saturated solution and extracted with EtOAc (3 × 10 mL). The combined organic phases were dried with MgSO_4_, filtered, and concentrated under reduced pressure. The crude product was purified by flash column chromatography on silica gel, (petroleum ether/EtOAc/toluene 7:2:1) to afford **4** as a pale-yellow solid (49 mg; 68%). ^1^H-NMR (400 MHz, CDCl_3_) δ 7.76–7.69 (m, 2H), 7.55–7.49 (m, 2H), 7.44 (ddd, *J* = 8.6, 2.1, 0.6 Hz, 1H), 7.37–7.30 (m, 3H), 5.06 (ddt, *J* = 50.8, 4.5, 3.4 Hz, 1H), 4.76 (dd, *J* = 23.3, 6.0 Hz, 1H), 4.13 (ddd, *J* = 15.3, 14.3, 4.4 Hz, 1H), 3.91 (ddd, *J* = 32.0, 14.3, 3.5 Hz, 1H), 2.52 (d, *J* = 9.0 Hz, 1H), 1.54 (s, 9H). ^19^F-NMR (376 MHz, CDCl_3_) δ −200.41. ^13^C-NMR (101 MHz, CDCl_3_) δ 153.5, 136.7, 131.7, 131.3, 130.5, 128.5, 128.3, 127.9, 123.4, 123.2, 118.9, 89.4, 89.3 (C-F, ^1^*J*_CF_ = 176.8 Hz), 89.1, 87.6 (C-F, ^1^*J*_CF_ = 176.8 Hz), 82.3, 67.7 (C-F, ^2^*J*_CF_ = 20.2 Hz), 67.5 (C-F, ^2^*J*_CF_ = 20.2 Hz), 46.6 (C-F, ^2^*J*_CF_ = 23.2 Hz), 46.4 (C-F, ^2^*J*_CF_ = 23.2 Hz), 28.4. MS (ESI) [M − Boc + H]^+^ = 267.

### 3.6. N-Boc Deprotection

(3R,4S)-3-Fluoro-1,2,3,4-tetrahydroquinolin-4-ol (**5**). *Tert*-Butyl (3*R*,4*S*)-3-fluoro-4-hydroxy-3,4-dihydroquinoline-1(2*H*)-carboxylate 3a (200 mg; 0.75 mmol; 1.0 equiv) was introduced in a 50 mL round-bottom tube set under argon. 3.0 mL of 1,4-dioxane were added followed by 5.0 mL of HPLC-grade H_2_O. The mixture was heated to reflux for 34 h [26]. It was then allowed to cool down to room temperature, and it was extracted with MTBE (3 × 20 mL). The combined organic phases were dried with MgSO_4_, filtered, and concentrated under reduced pressure. The crude product was purified by flash column chromatography on silica gel (petroleum ether/EtOAc 8:2 to 7:3) to afford 5 as a pale-yellow solid (112 mg; 90%). ^1^H-NMR (400 MHz, CDCl_3_) δ 7.35 (dt, *J* = 7.8, 1.0 Hz, 1H), 7.11 (ddd, *J* = 8.5, 7.5, 1.6 Hz, 1H), 6.75 (td, *J* = 7.4, 1.2 Hz, 1H), 6.55 (dd, *J* = 8.0, 1.1 Hz, 1H), 5.08–4.75 (m, 2H), 3.87 (s, 1H), 3.66 (dt, *J* = 11.9, 7.9 Hz, 1H), 3.45 (dddd, *J* = 22.2, 11.9, 3.3, 1.4 Hz, 1H), 2.26 (s, 1H). ^19^F-NMR (376 MHz, CDCl_3_) δ –203.21. ^13^C-NMR (101 MHz, CDCl_3_) δ 143.4, 129.7, 129.6, 120.0, 118.2, 114.2, 88.8 (C-F, ^1^*J*_CF_ = 176.8 Hz), 87.1 (C-F, ^1^*J*_CF_ = 176.8 Hz), 67.3 (C-F, ^2^*J*_CF_ = 19.2 Hz), 67.1 (C-F, ^2^*J*_CF_ = 19.2 Hz), 41.8 (C-F, ^2^*J*_CF_ = 24.2 Hz), 41.6 (C-F, ^2^*J*_CF_ = 24.2 Hz). HRMS (ESI) *m*/*z*: [M+H]^+^ calcd. for C_9_H_11_FNO 168.0819. Found 168.0819.

## 4. Conclusions

In summary, a straightforward method to access enantiomerically and diastereomerically enriched *cis-*fluoro-dihydrotetrahydroquinolinols has been developed. Ru catalyst (*R,R*)-TsDENEB ensures an efficient asymmetric transfer hydrogenation/dynamic kinetic resolution (ATH/DKR) under mild conditions (acetonitrile as solvent and heating at 40 °C) and low catalyst loading (0.5 mol%). The reaction was performed successfully on a series of 3-fluoro-dihydrotetrahydroquinolin-4-ones substituted with several electron-donating or electron-withdrawing groups with excellent yields and diastereo- and enantioselectivities.

## Figures and Tables

**Table 1:**
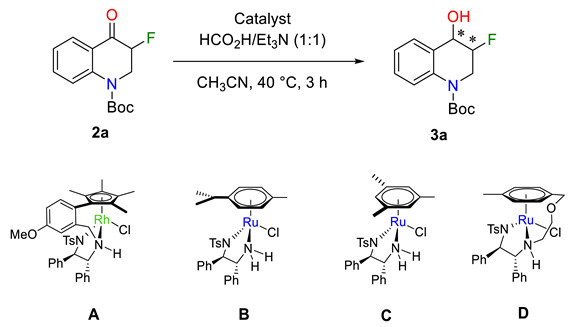
Catalyst screening for the ATH/DKR ^a^.

Entry	Catalyst	Yield ^b^	dr ^c^	ee ^d^
1	A	95%	79:21	95%
2	B	93%	97:3	99%
3	C	93%	97:3	98%
4	D	94%	98:2	>99%

^a^ Conditions: **2a** (0.32 mmol), catalyst (0.5 mol%), HCO_2_H/Et_3_N (1:1) (6 equiv), CH_3_CN (1 mL), 40 °C, 3 h. ^b^ Isolated yield. ^c^ Determined by ^1^H-NMR analysis of the crude mixture. ^d^ Determined by SFC analysis. See Supplementary Materials.

**Table 2:**
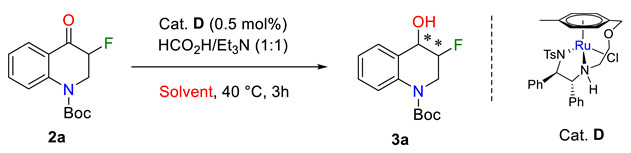
Solvent optimization ^a^.

Entry	Solvent	Conversion ^b^	Yield ^c^	dr ^b^	ee ^d^
1	CH_3_CN	100%	93%	98:2	>99%
2	CH_2_Cl_2_	83%	78%	98:2	95%
3	EtOAc	48%	46%	98:2	98%
4	THF	38%	36%	98:2	>99%
5	Me-THF	18%	15%	96:4	98%
6	Toluene	26%	26%	97:3	96%
7	Cl-benzene	43%	42%	97:3	94%
8	CF_3_-benzene	60%	57%	98:2	96%
9	MeOH	76%	73%	98:2	96%
10	*i*PrOH	95%	94%	98:2	98%
11	HFIP	26%	24%	98:2	>99%

^a^ Conditions: **2a** (0.32 mmol), catalyst (0.5 mol%), HCO_2_H/Et_3_N (1:1) (6 equiv), solvent (1 mL), 40 °C, 3 h. ^b^ Determined by ^1^H-NMR analysis of the crude mixture. ^c^ Isolated yield. ^d^ Determined by SFC analysis.

**Table 3:**
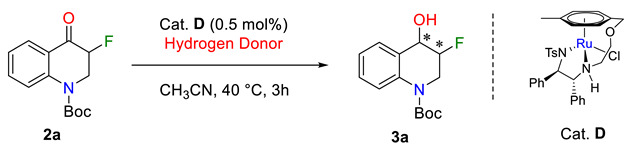
Survey of the hydrogen donor ^a^.

Entry	Hydrogen Donor	Conversion	Yield ^b^	dr ^c^	ee ^d^
1	HCO_2_H/Et_3_N (1:1)	100%	93%	98:2	>99%
2	HCO_2_H/Et_3_N (2:5)	100%	95%	98:2	99%
3	HCO_2_H/Et_3_N (5:2)	100%	94%	54:46	92%
4	HCO_2_H/Et_3_N (12:1)	0%	-	-	-
5	HCO_2_H /DBU (1:1)	90%	88%	98:2	99%
6	HCO_2_H /DABCO (1:1)	75%	73%	98:2	98%
7	HCO_2_NH_4_	35%	34%	98:2	99%
8 ^e^	(HCO_2_)_2_Ca	20%	16%	98:2	98%
9 ^f^	HCO_2_H /Et_3_N (1:1)	54%	53%	98:2	>99%
10 ^g^	HCO_2_H /Et_3_N (1:1)	30%	23%	98:2	>99%

^a^ Conditions: **2a** (0.32 mmol), catalyst (0.5 mol%), HCO_2_H/Et_3_N (1:1) (6 equiv), CH_3_CN (1 mL), 40 °C, 3 h. ^b^ Isolated yield. ^c^ Determined by ^1^H-NMR analysis of the crude mixture. ^d^ Determined by SFC analysis. ^e^ CH_3_CN/H_2_O (10:1) was used as solvent. ^f^ Catalyst loading of 0.2 mol% and reaction time of 24 h. ^g^ Reaction run at r. t. (22 °C) for 24 h.

## Data Availability

CCDC 2132626 contains the supplementary crystallographic data for this paper. These data can be obtained free of charge via www.ccdc.cam.ac.uk/data_request/cif (accessed on 5 January 2022), or by emailing data_request@ccdc.cam.ac.uk, or by contacting The Cambridge Crystallographic Data Centre, 12 Union Road, Cambridge CB2 1EZ, UK; Fax: +44-1223-336033.

## Supplementary Materials

A supporting information including NMR and SFC spectra, is available online.

## Sample Availability

Samples of the compounds are not available from the authors.
