# Supplementary material for: Straightforward Access to Enantioenriched cis-3-Fluoro-dihydroquinolin-4-ols Derivatives via Ru(II)-Catalyzed-Asymmetric Transfer Hydrogenation/Dynamic Kinetic Resolution
Source: Molecules. 2022 Feb 1;27(3):995. doi: 10.3390/molecules27030995 (PMC8838918; doi:10.3390/molecules27030995)

# SUPPORTING INFORMATION

# Straightforward Access to Enantioenriched *cis*-3-Fluoro-Dihydroquinolin-4-ols Derivatives *via* Ru(II)-Catalyzed-Asymmetric Transfer Hydrogenation/Dynamic Kinetic Resolution

Ricardo Molina Betancourt, Phannarath Phansavath* and Virginie Ratovelomanana-Vidal*

*PSL University, Chimie ParisTech, CNRS, Institute of Chemistry for Life and Health Sciences, CSB2D team, 75005 Paris, France*

*E-mail: phannarath.phansavath@chimieparistech.psl.eu; virginie.vidal@chimieparistech.psl.eu*

## Contents

**1) General information** **1**

**2) NMR spectra** **2**

**3) SFC spectra** **46**

### 1) General Information

All air and/or water sensitive reactions were carried out under an argon atmosphere. THF, $CH_2Cl_2$ and toluene were dried over alumina columns in a solvent purification apparatus (Innovative technology). Methanol, isopropanol, chlorobenzene, trifluoromethylbenzene, ethyl acetate and acetonitrile from Sigma-Aldrich were used without further purification. Hexafluoroisopropanol (HFIP) was purchased from TCI and was used without further purification. Formic acid/triethylamine (1:1) mixture was purchased from Fluka or Alfa Aesar and was used without further purification. Reactions were monitored by thin layer chromatography carried out on precoated silica gel plates (Merck 60F254) and revealed with either an ultra-violet lamp ($\lambda$ = 254 nm) or a potassium permanganate solution. Proton nuclear magnetic resonance ($^1$H NMR) spectra were recorded using a Bruker AC 400 (400 MHz). The chemical shifts are expressed in parts per million (ppm) referenced to residual chloroform (7.26 ppm). Data are reported as follows: chemical shifts ($\delta$), multiplicity (recorded as s, singlet; d, doublet; t, triplet; q, quadruplet; quint, quintuplet; sext, sextuplet; hept, heptuplet; m, multiplet and br, broad), coupling constants and integration. Carbon-13 nuclear magnetic resonance ($^{13}$C NMR) spectra were recorded using a Bruker AC 400 (101 MHz). The chemical shifts are expressed in parts per million (ppm) relative to the centre line of the triplet at 77.16 ppm for $CDCl_3$. Melting points (m. p.) were determined on a Köfler melting point apparatus. Optical rotations were measured on a Jasco P-1010 polarimeter. High resolution mass spectrometric (HRMS) analyses were measured on LTQ-Orbitrap (Thermo Fisher Scientific) at Sorbonne Université.

## 2) NMR Spectra

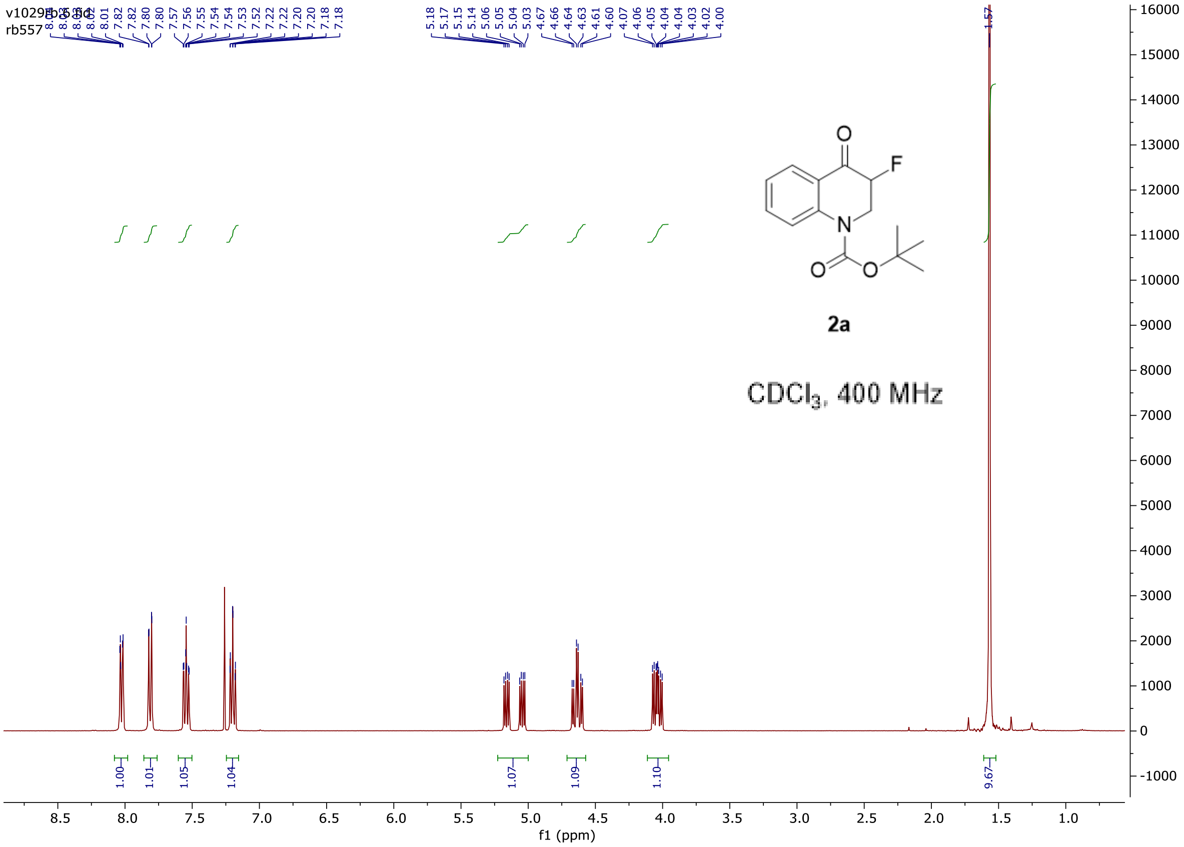


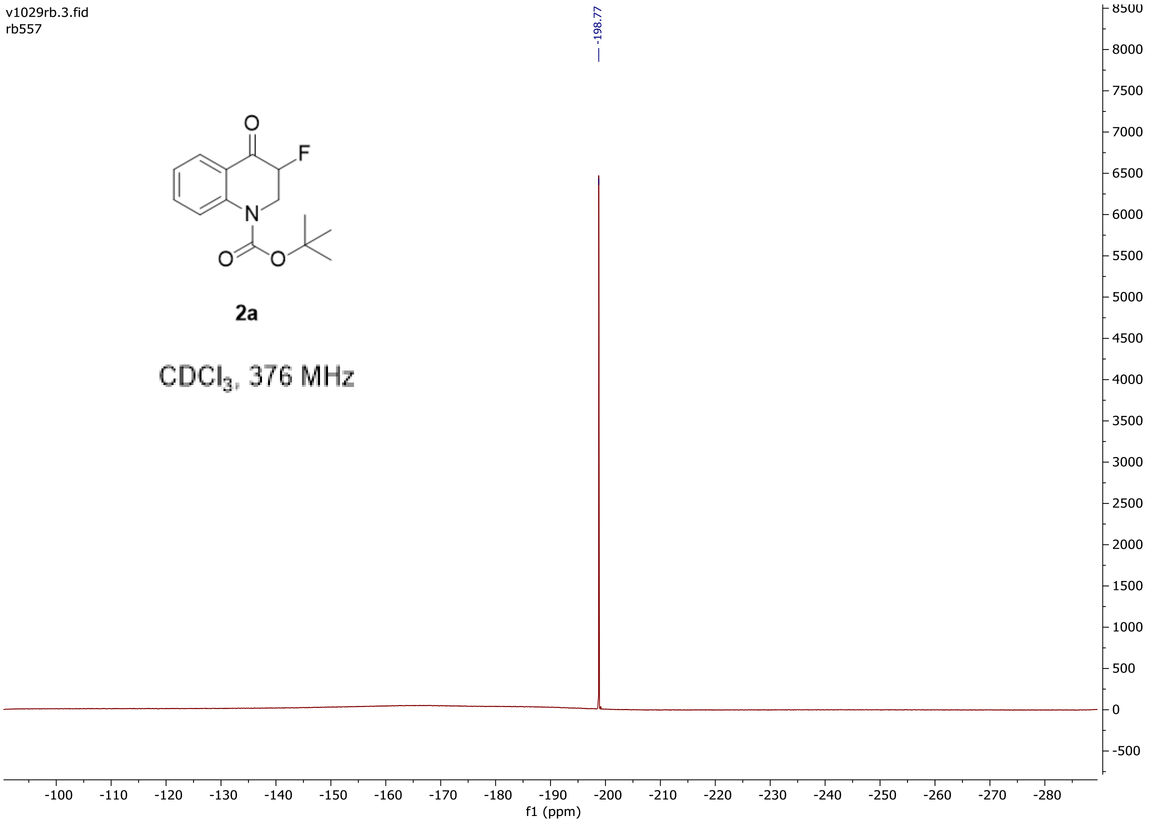

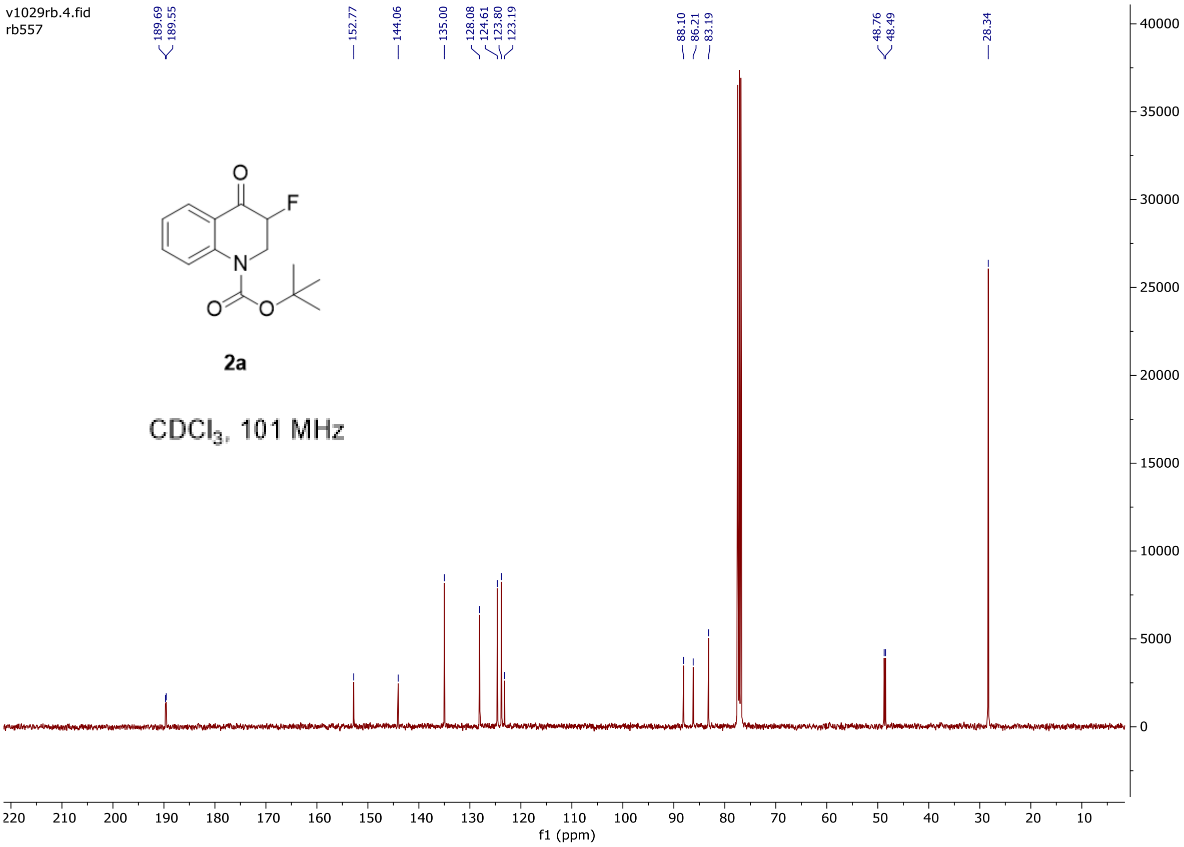
v1029rb.4.fid
rb557
189.69
189.55
152.77
144.06
135.00
128.08
124.61
123.80
123.19
88.10
86.21
83.19
48.76
48.49
28.34
O
F
N
O
O
2a
CDCl3, 101 MHz
f1 (ppm)

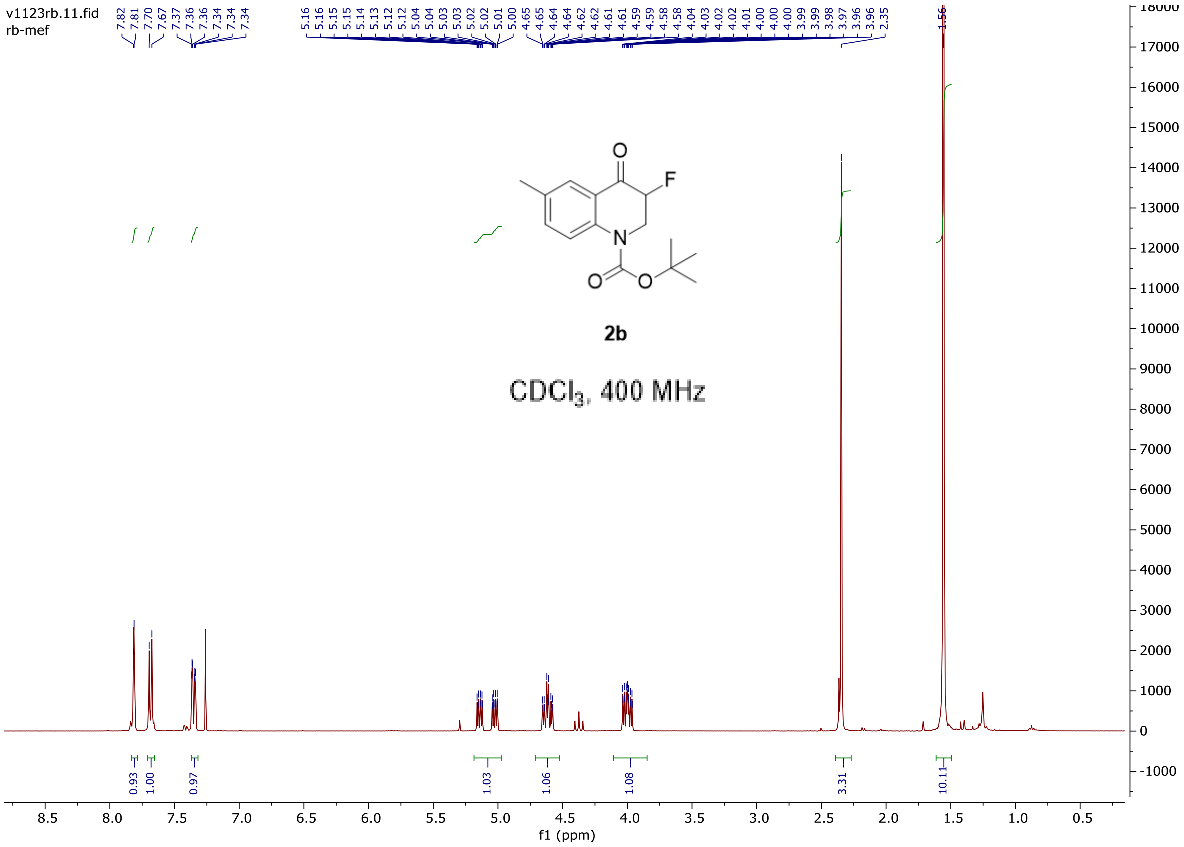
v1123rb.11.fid
rb-mef
7.82
7.81
7.70
7.67
7.37
7.36
7.36
7.34
7.34
7.34
5.16
5.16
5.15
5.15
5.14
5.13
5.12
5.12
5.04
5.04
5.03
5.03
5.02
5.02
5.01
5.00
4.65
4.65
4.64
4.64
4.62
4.62
4.61
4.61
4.59
4.59
4.58
4.58
4.04
4.03
4.03
4.02
4.02
4.01
4.00
4.00
4.00
3.99
3.99
3.98
3.97
3.96
3.96
2.35
1.56
O
F
N
O
O
2b
CDCl3, 400 MHz
0.93
1.00
0.97
1.03
1.06
1.08
3.31
10.11
f1 (ppm)

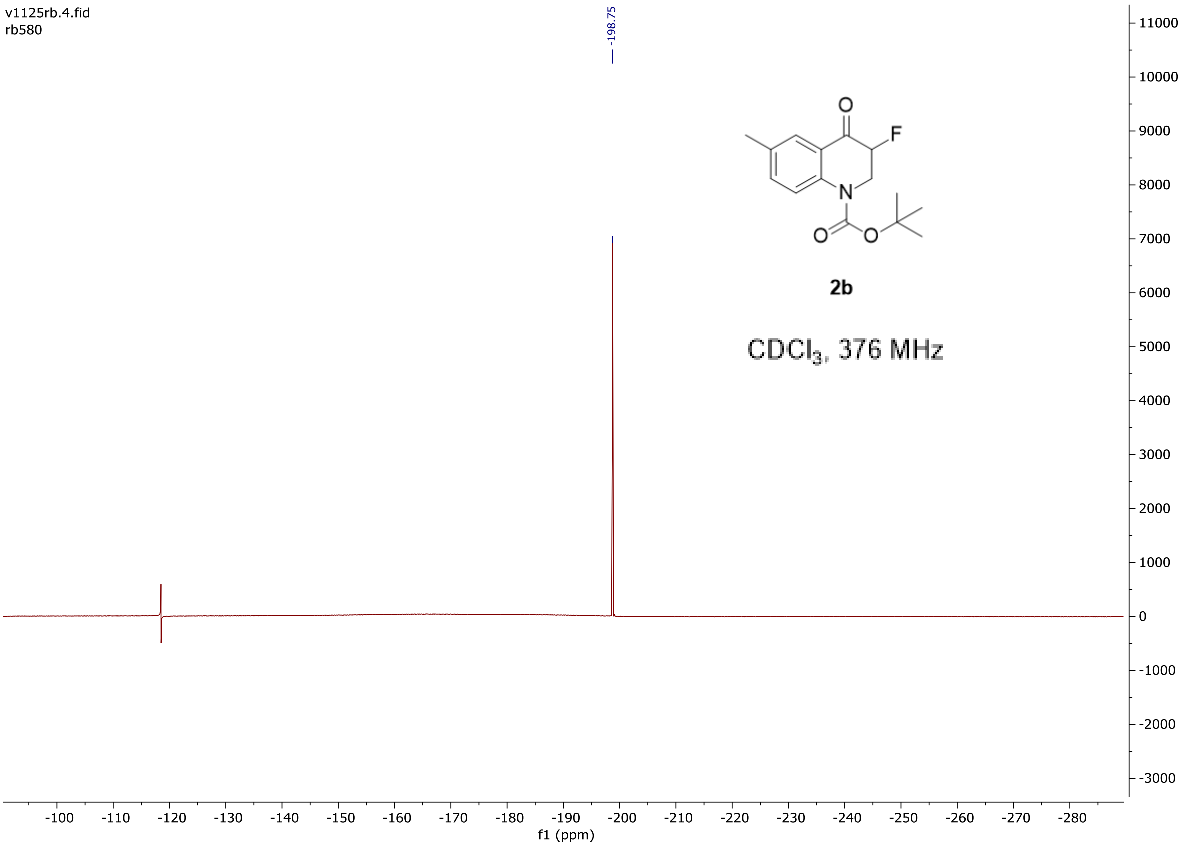
v1125rb.4.fid
rb580
-198.75
O
F
N
O
O
2b
CDCl3, 376 MHz
f1 (ppm)

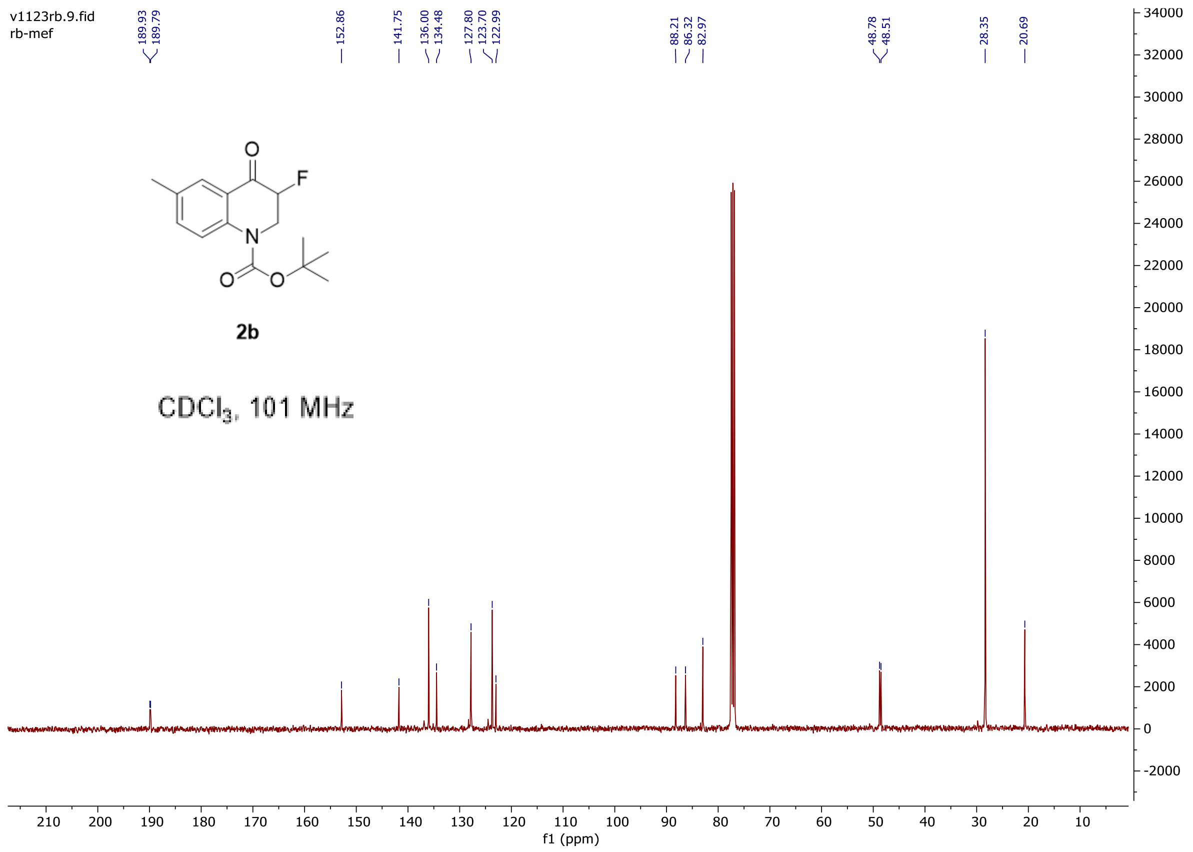
v1123rb.9.fid
rb-mef
189.93
189.79
152.86
141.75
136.00
134.48
127.80
123.70
122.99
88.21
86.32
82.97
48.78
48.51
28.35
20.69
O
F
N
O
O
2b
CDCl3, 101 MHz
f1 (ppm)

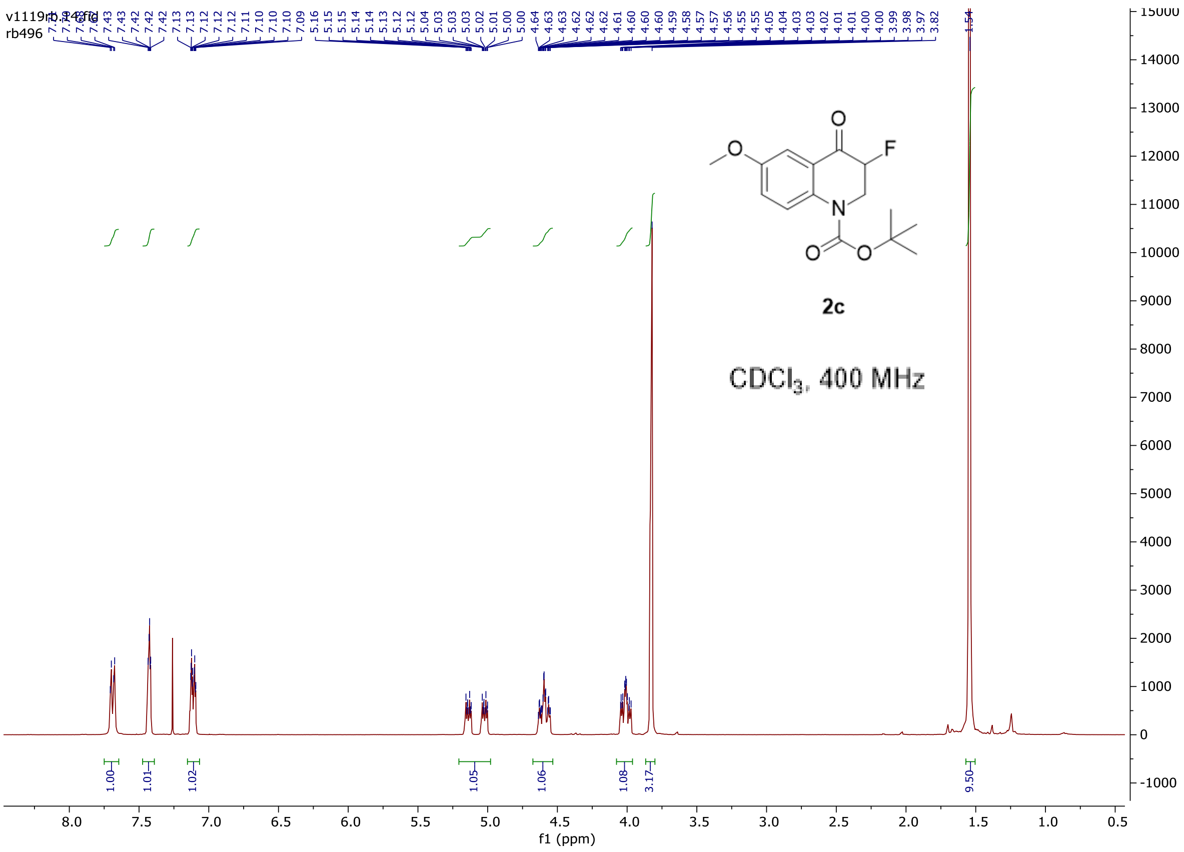
v1119rb.14.fid
rb496
2c
CDCl3, 400 MHz
f1 (ppm)

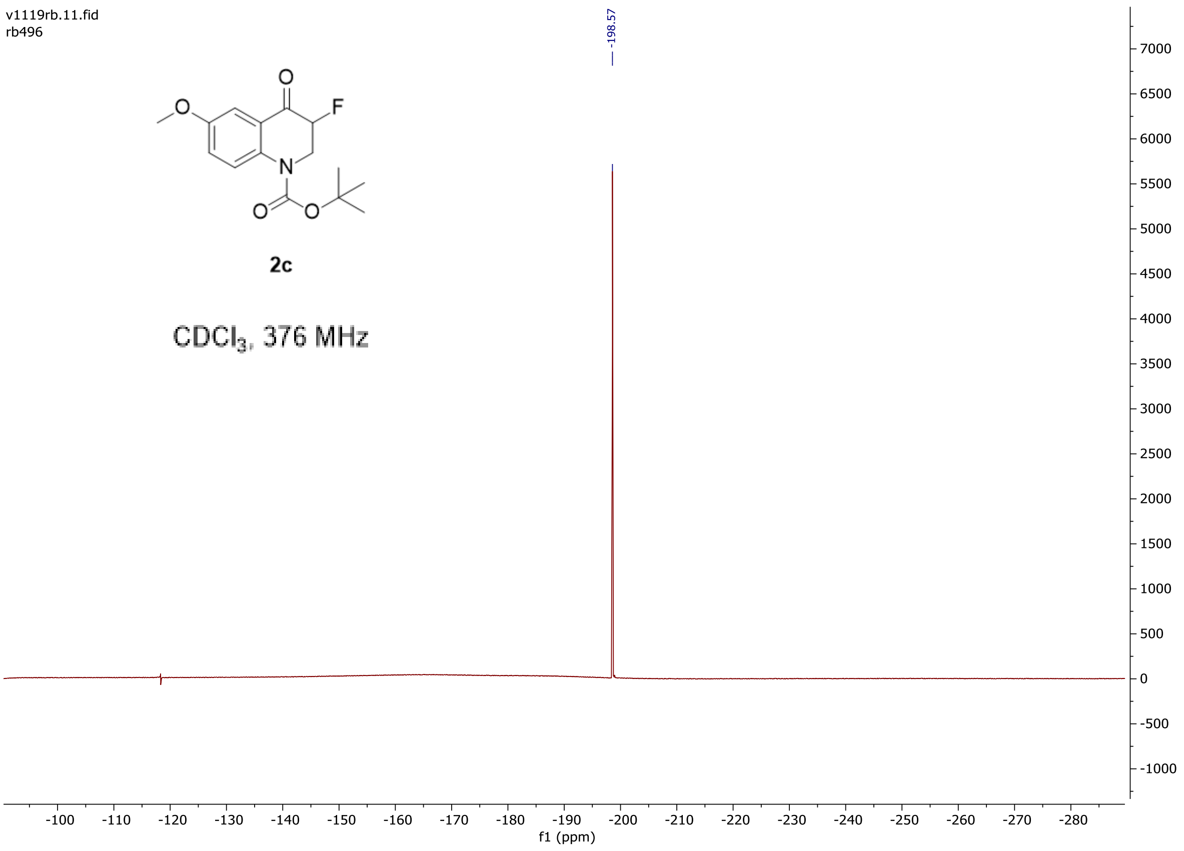
v1119rb.11.fid
rb496
-198.57
2c
CDCl3, 376 MHz
f1 (ppm)

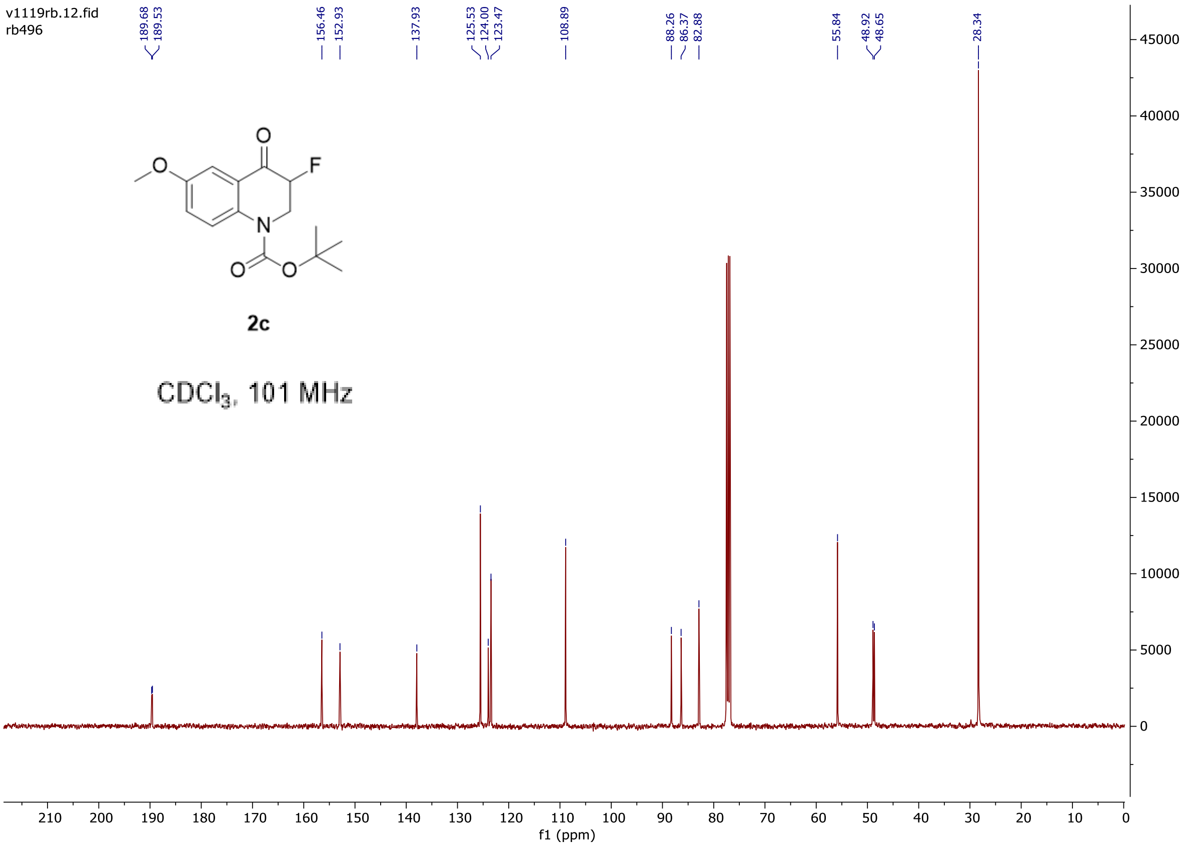

v1119rb.12.fid
rb496
189.68
189.53
156.46
152.93
137.93
125.53
124.00
123.47
108.89
88.26
86.37
82.88
55.84
48.92
48.65
28.34
2c
CDCl3, 101 MHz
f1 (ppm)

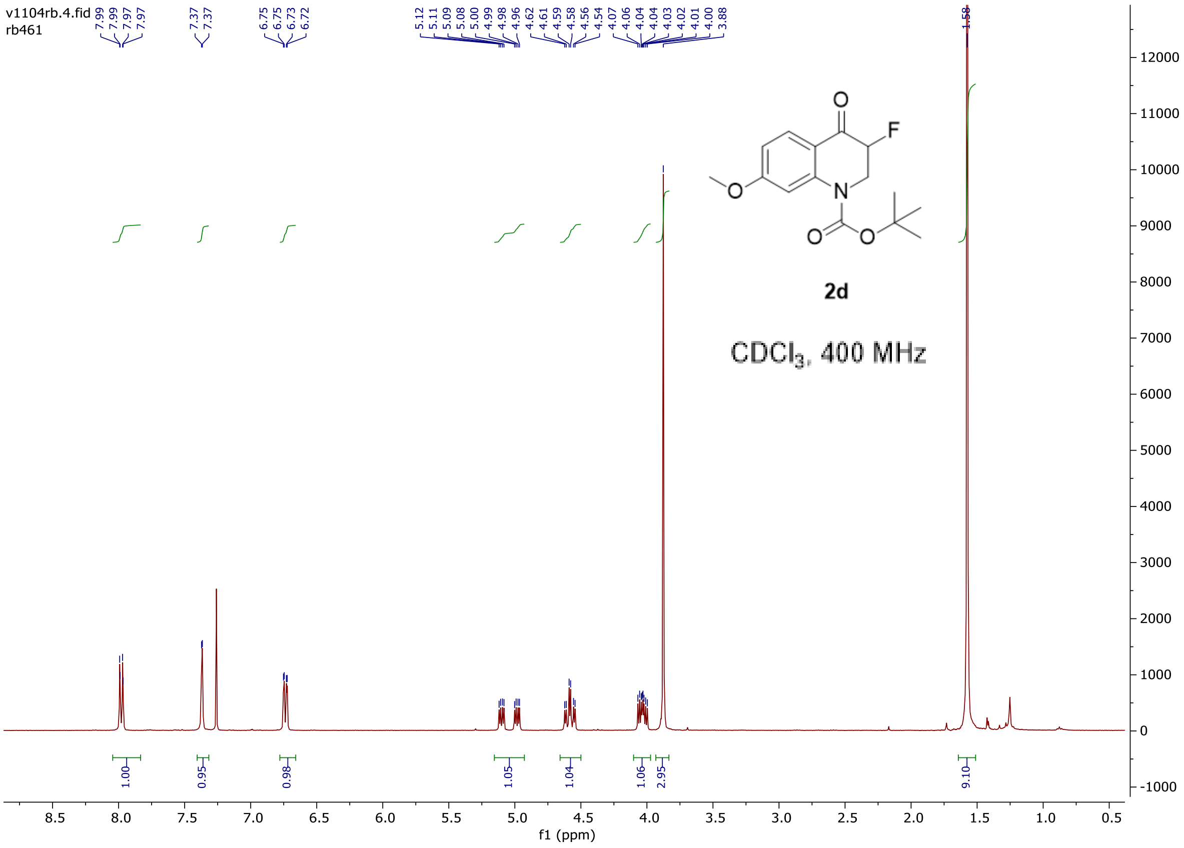
v1104rb.4.fid
rb461
2d
CDCl3, 400 MHz
f1 (ppm)

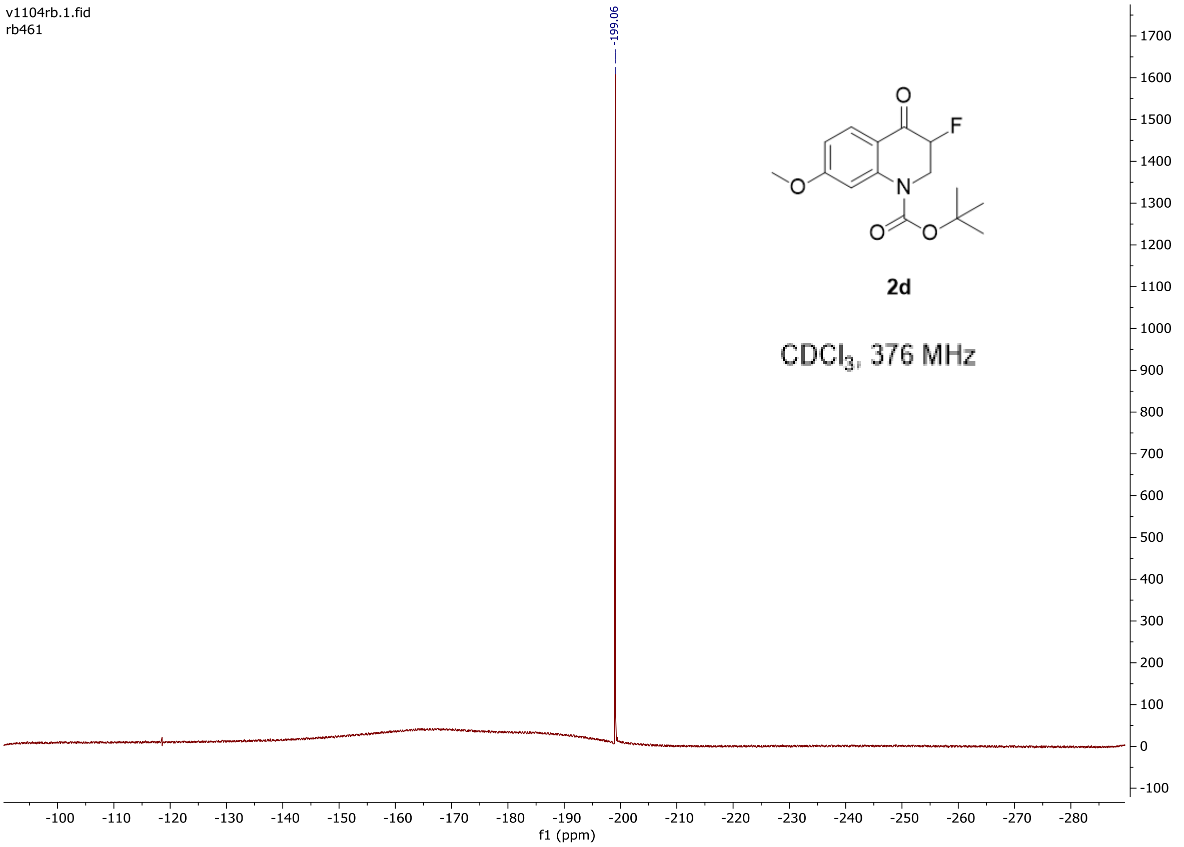
v1104rb.1.fid
rb461
-199.06
2d
CDCl3, 376 MHz
f1 (ppm)

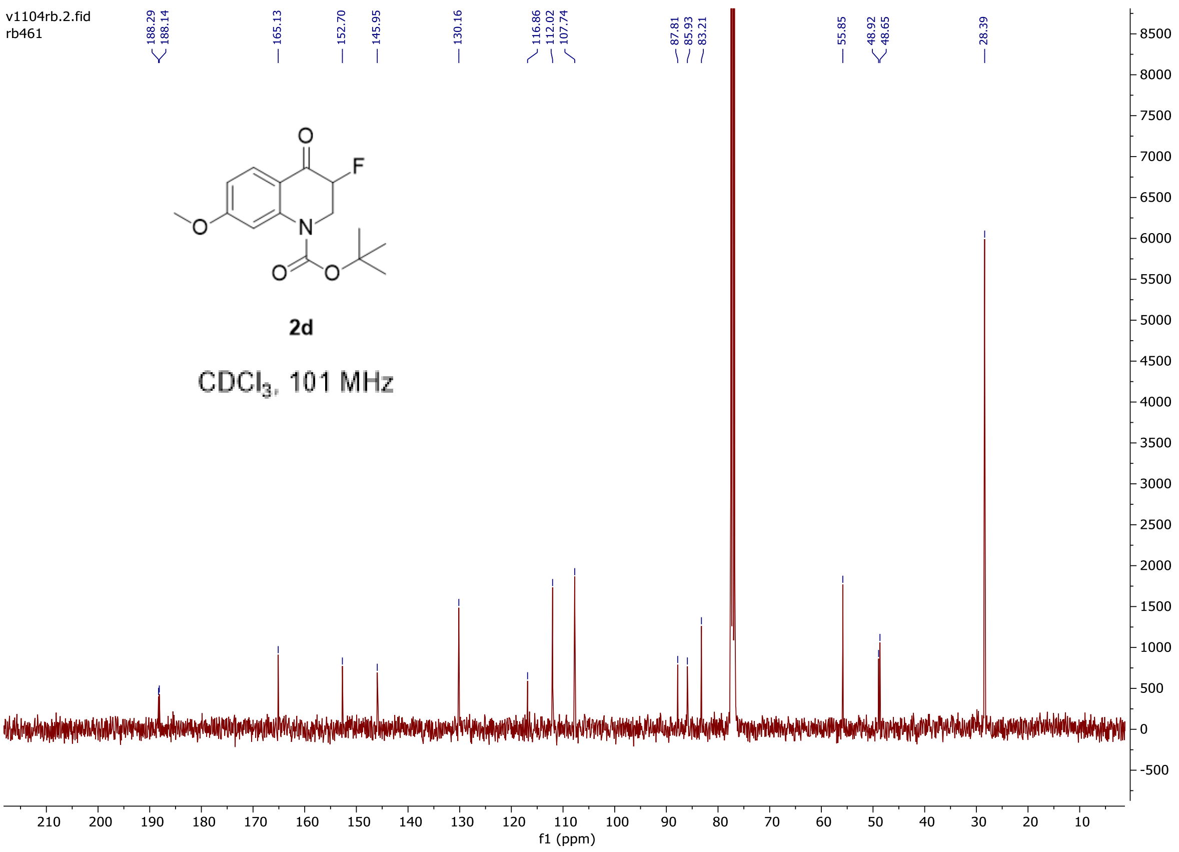
v1104rb.2.fid
rb461
188.29
188.14
165.13
152.70
145.95
130.16
116.86
112.02
107.74
87.81
85.93
83.21
55.85
48.92
48.65
28.39
2d
CDCl3, 101 MHz
f1 (ppm)

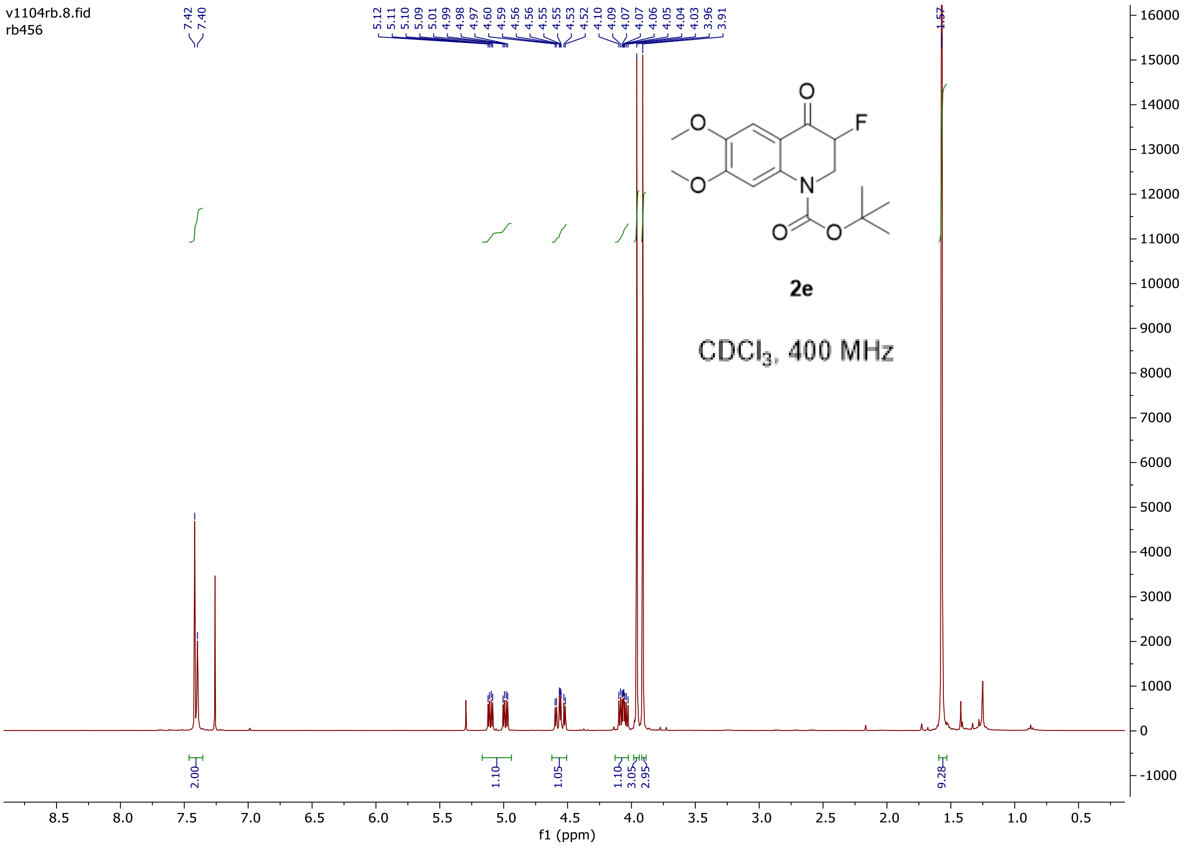
v1104rb.8.fid
rb456
7.42
7.40
5.12 5.11 5.10 5.09 5.01 4.99 4.98 4.97 4.60 4.59 4.56 4.56 4.55 4.55 4.53 4.52 4.10 4.09 4.07 4.07 4.06 4.05 4.04 4.03 3.96 3.91
1.57
O
F
O
O
N
O
O
2e
CDCl3, 400 MHz
2.00
1.10
1.05
1.10
3.05
2.95
9.28
8.5 8.0 7.5 7.0 6.5 6.0 5.5 5.0 4.5 4.0 3.5 3.0 2.5 2.0 1.5 1.0 0.5
f1 (ppm)
16000 15000 14000 13000 12000 11000 10000 9000 8000 7000 6000 5000 4000 3000 2000 1000 0 -1000

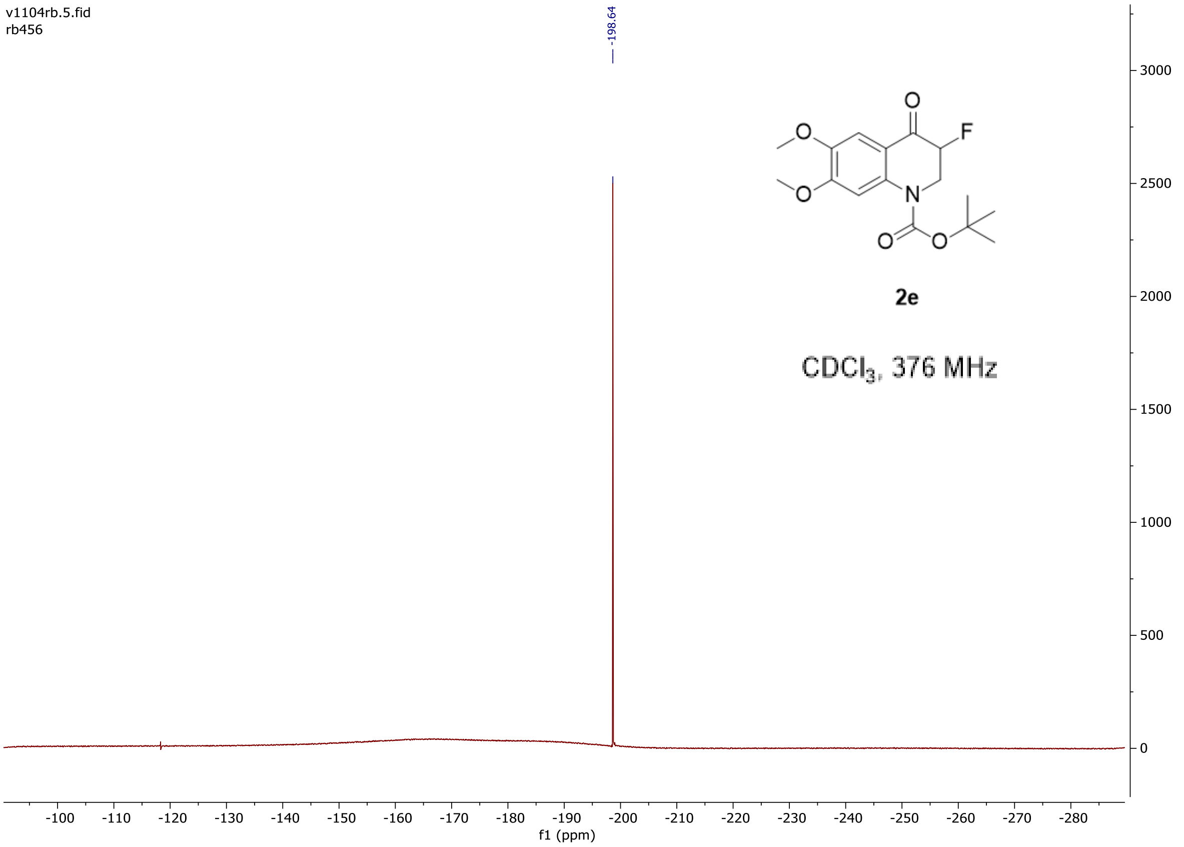
v1104rb.5.fid
rb456
-198.64
O
F
O
O
N
O
O
2e
CDCl3, 376 MHz
-100 -110 -120 -130 -140 -150 -160 -170 -180 -190 -200 -210 -220 -230 -240 -250 -260 -270 -280
f1 (ppm)
3000 2500 2000 1500 1000 500 0

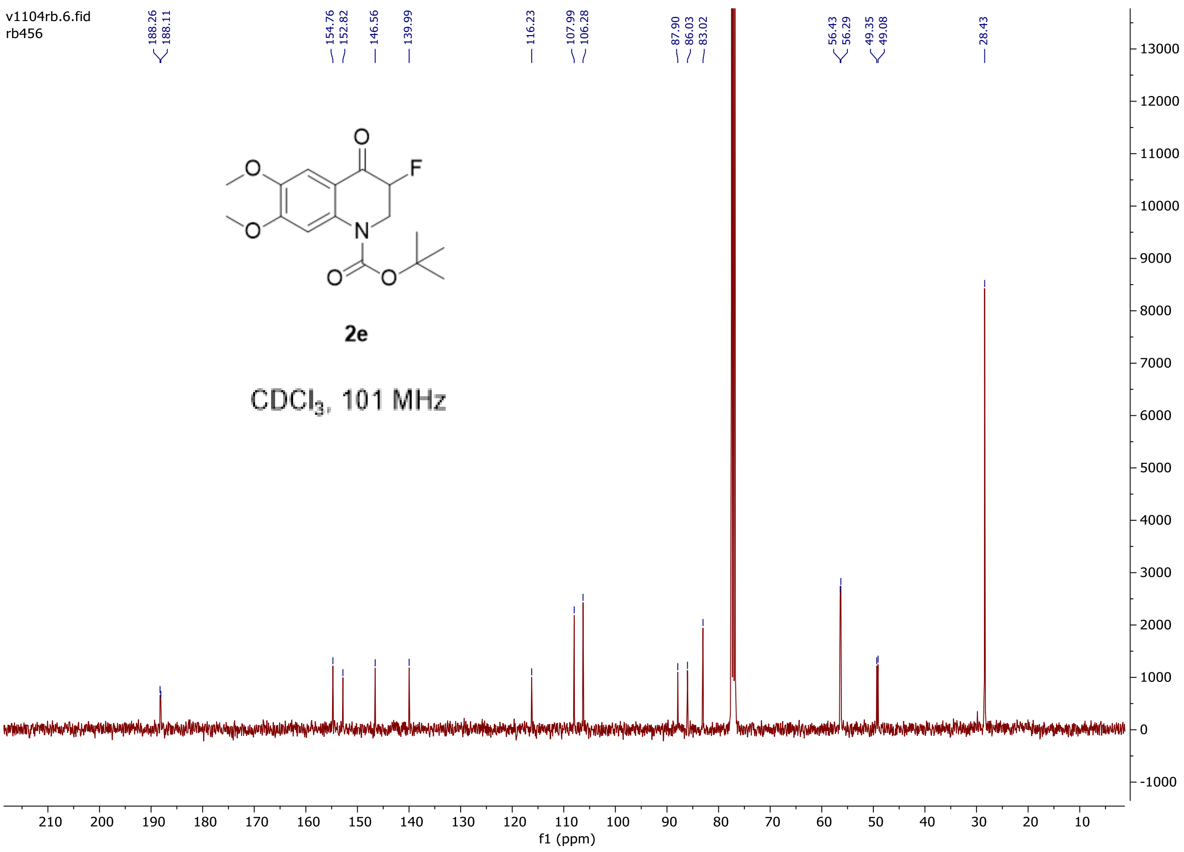
v1104rb.6.fid
rb456
188.26
188.11
154.76
152.82
146.56
139.99
116.23
107.99
106.28
87.90
86.03
83.02
56.43
56.29
49.35
49.08
28.43
2e
CDCl3, 101 MHz
f1 (ppm)

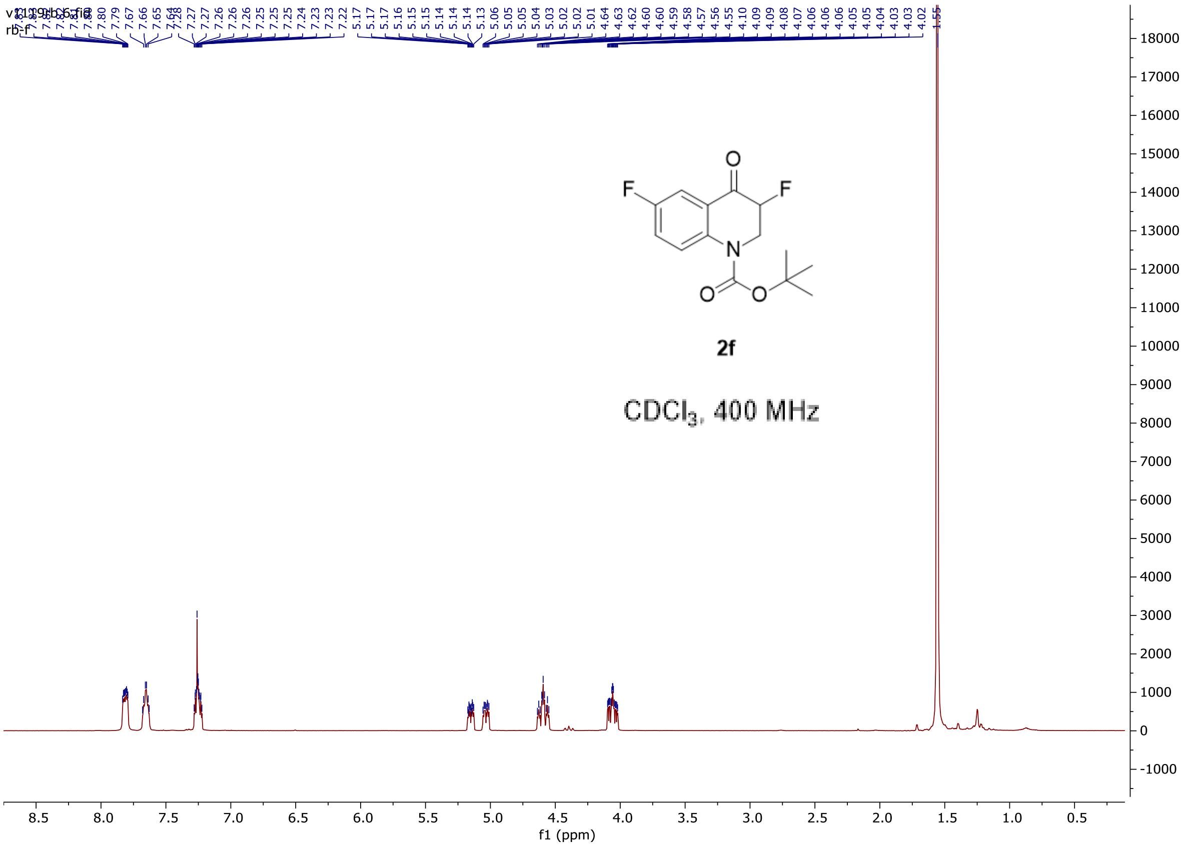
v1119rb.6.fid
rb-f
2f
CDCl3, 400 MHz
f1 (ppm)

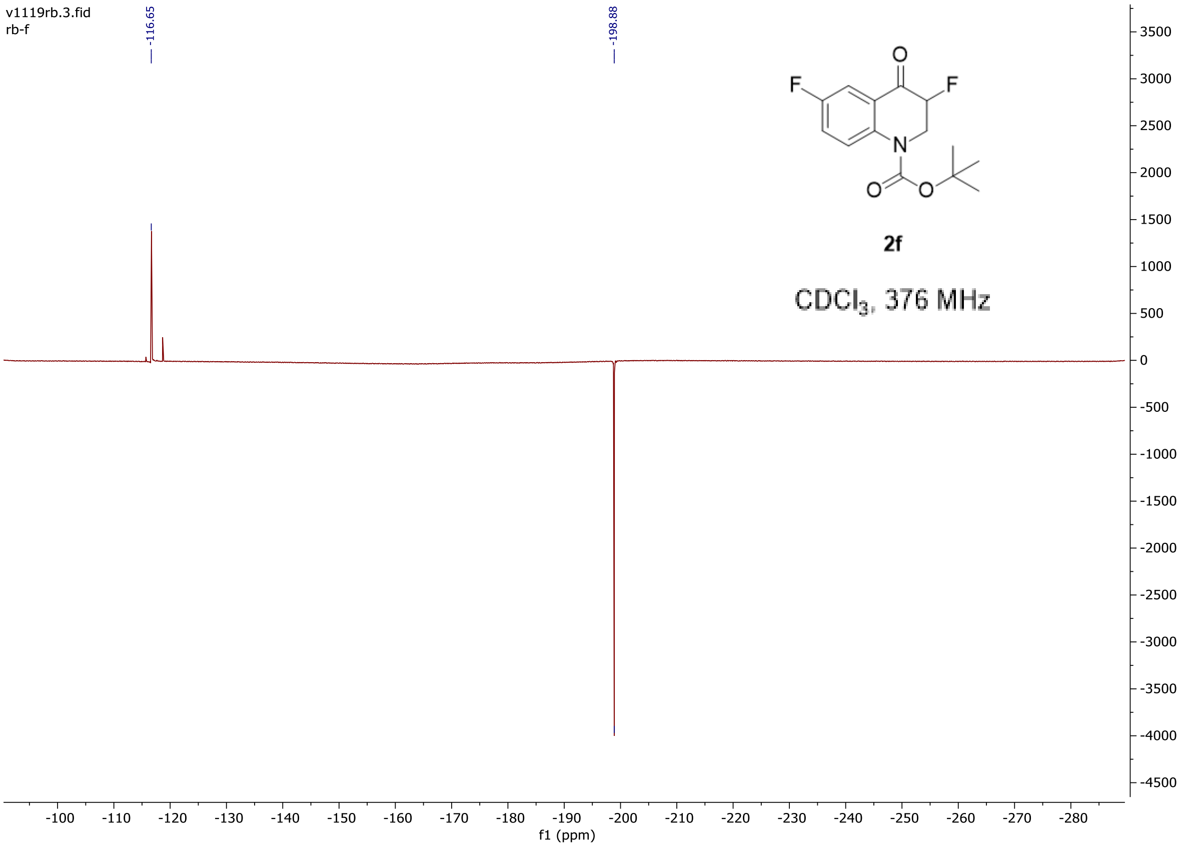
v1119rb.3.fid
rb-f
-116.65
-198.88
2f
CDCl3, 376 MHz
f1 (ppm)

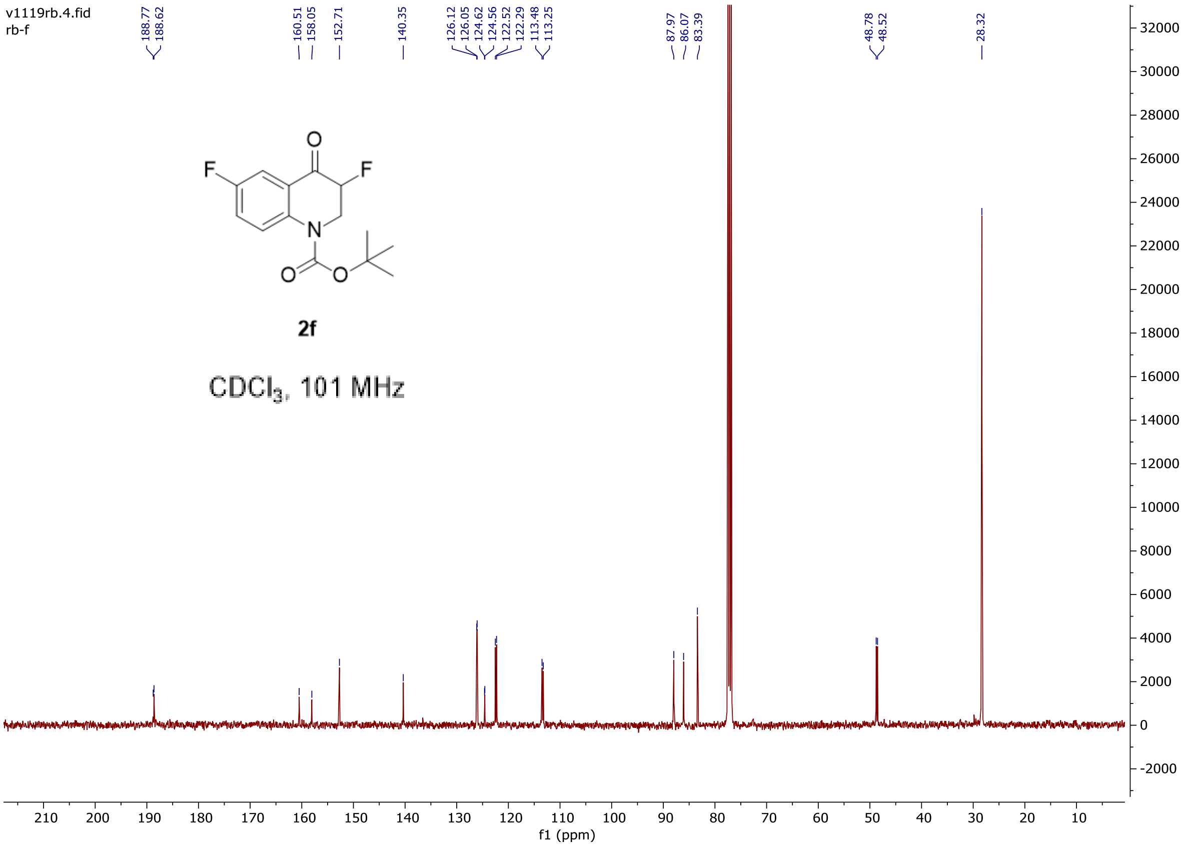
v1119rb.4.fid
rb-f
188.77
188.62
160.51
158.05
152.71
140.35
126.12
126.05
124.62
124.56
122.52
122.29
113.48
113.25
87.97
86.07
83.39
48.78
48.52
28.32
2f
CDCl3, 101 MHz
f1 (ppm)

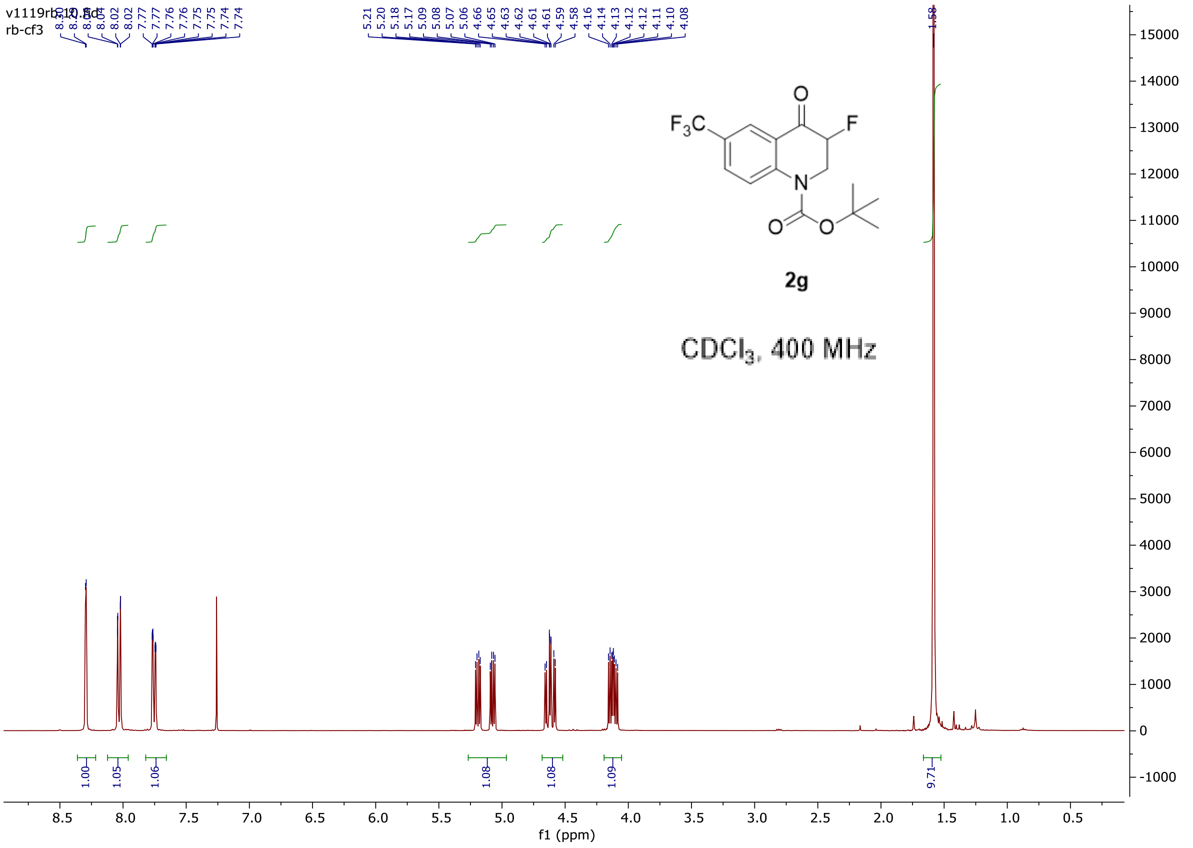
v1119rb.10.fid
rb-cf3
8.02
8.02
7.77
7.76
7.75
7.75
7.74
7.74
5.21
5.20
5.18
5.17
5.09
5.08
5.07
5.06
4.66
4.65
4.63
4.62
4.61
4.61
4.59
4.58
4.16
4.14
4.13
4.12
4.11
4.10
4.08
1.58
F3C
O
F
N
O
O
2g
CDCl3, 400 MHz
1.00
1.05
1.06
1.08
1.08
1.09
9.71
f1 (ppm)

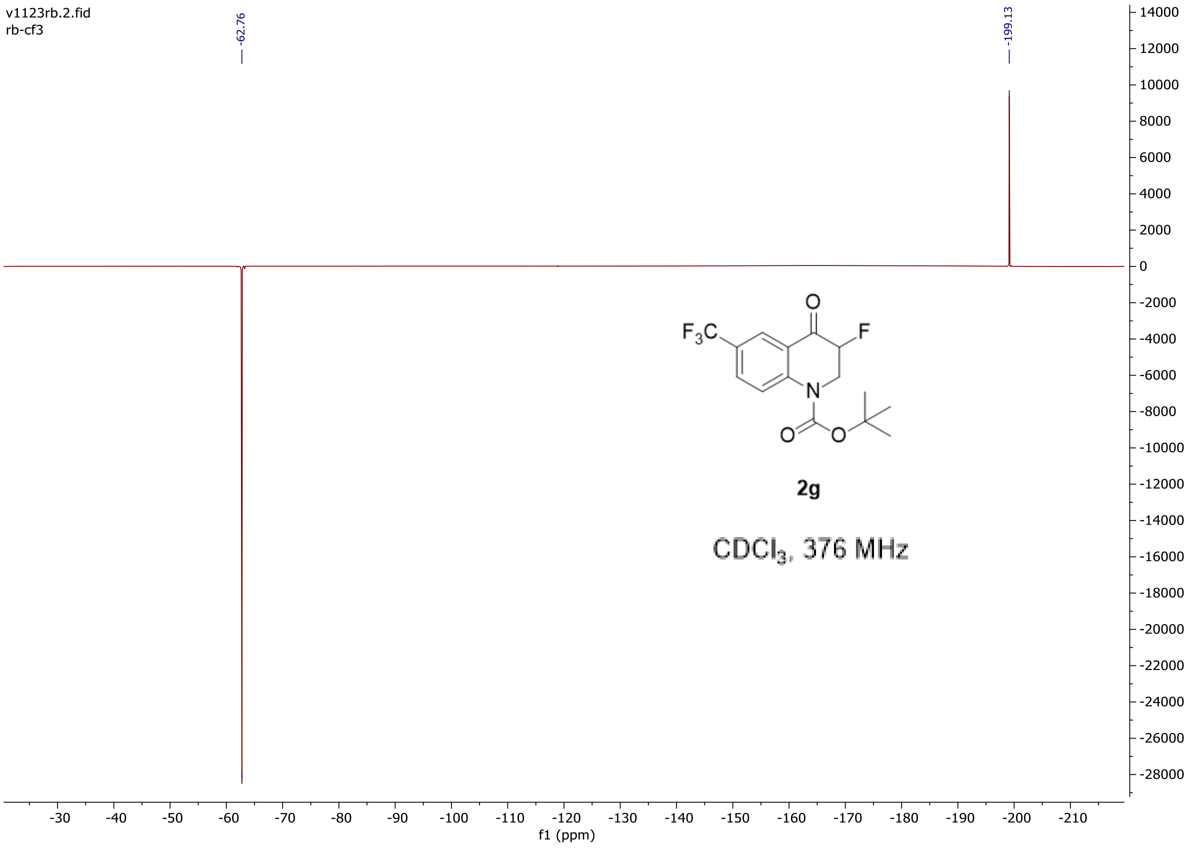
v1123rb.2.fid
rb-cf3
-62.76
-199.13
F3C
O
F
N
O
O
2g
CDCl3, 376 MHz
f1 (ppm)

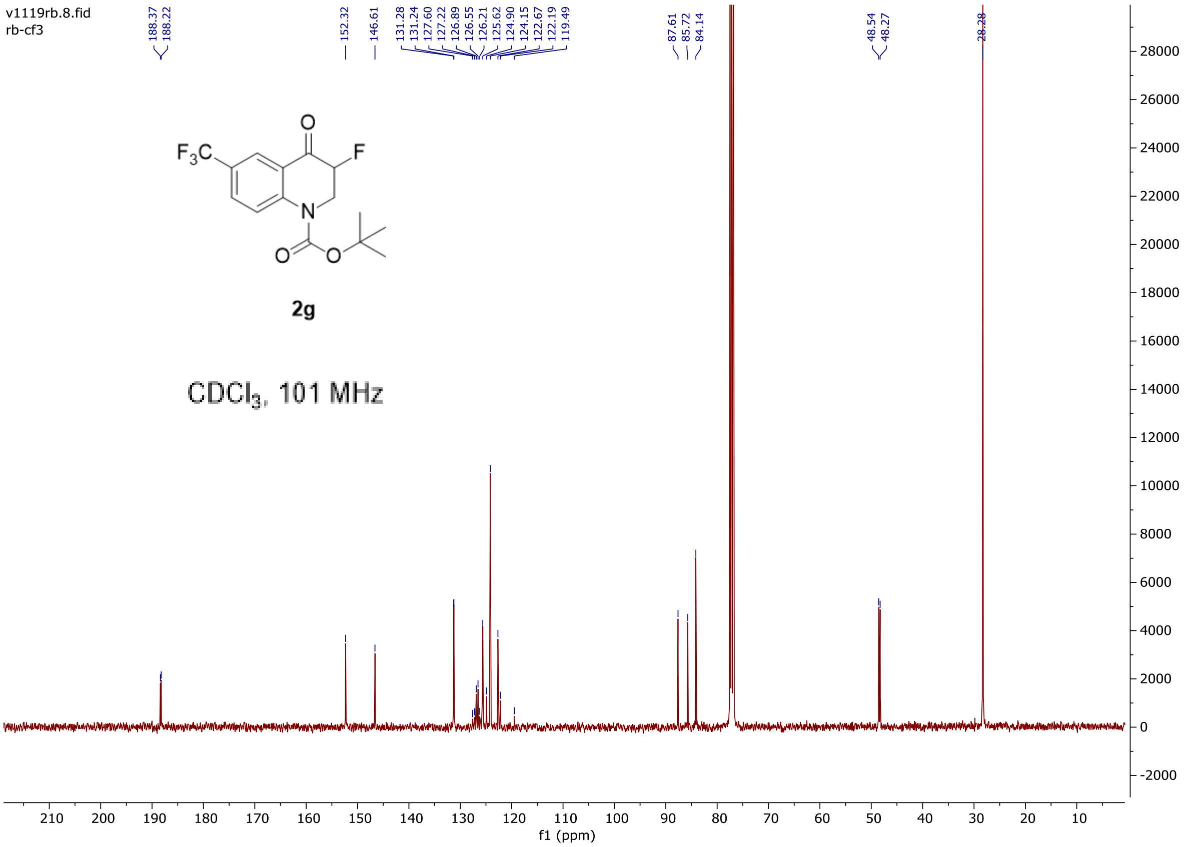


**2g**

CDCl$_3$, 101 MHz

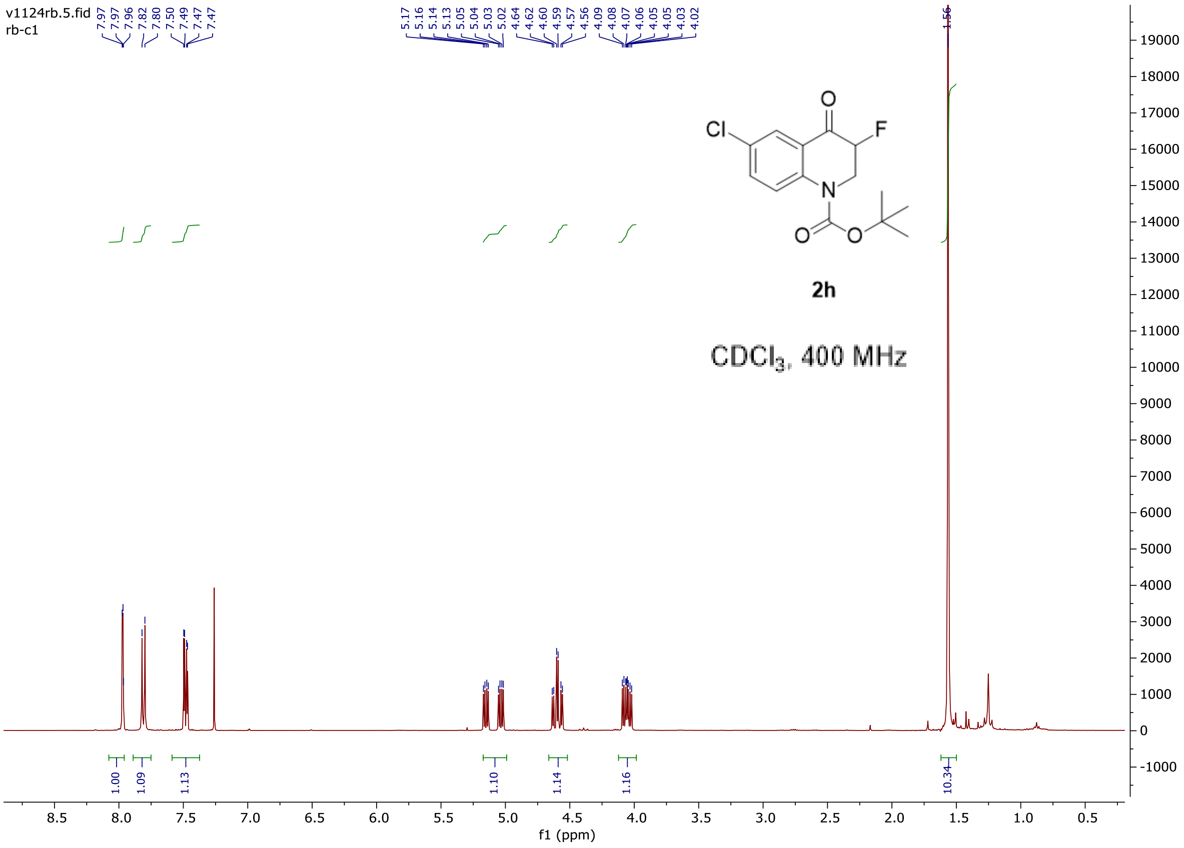
v1124rb.5.fid
rb-c1
7.97
7.97
7.96
7.82
7.80
7.50
7.49
7.47
7.47
5.17
5.16
5.14
5.13
5.05
5.04
5.03
5.03
5.02
4.64
4.62
4.60
4.59
4.57
4.56
4.09
4.08
4.07
4.06
4.05
4.05
4.03
4.02
1.56
Cl
O
F
N
O
O
2h
CDCl3, 400 MHz
1.00
1.09
1.13
1.10
1.14
1.16
10.34
f1 (ppm)

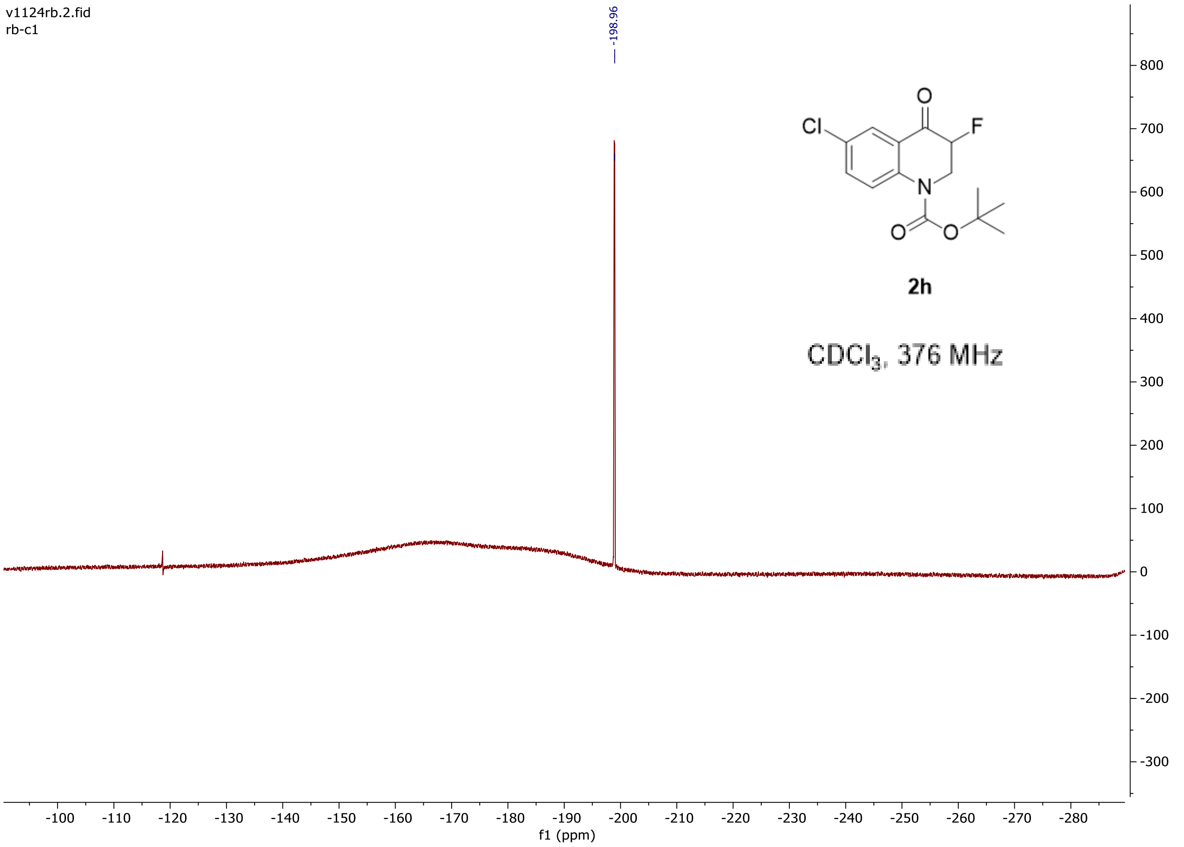
v1124rb.2.fid
rb-c1
-198.96
Cl
O
F
N
O
O
2h
CDCl3, 376 MHz
f1 (ppm)

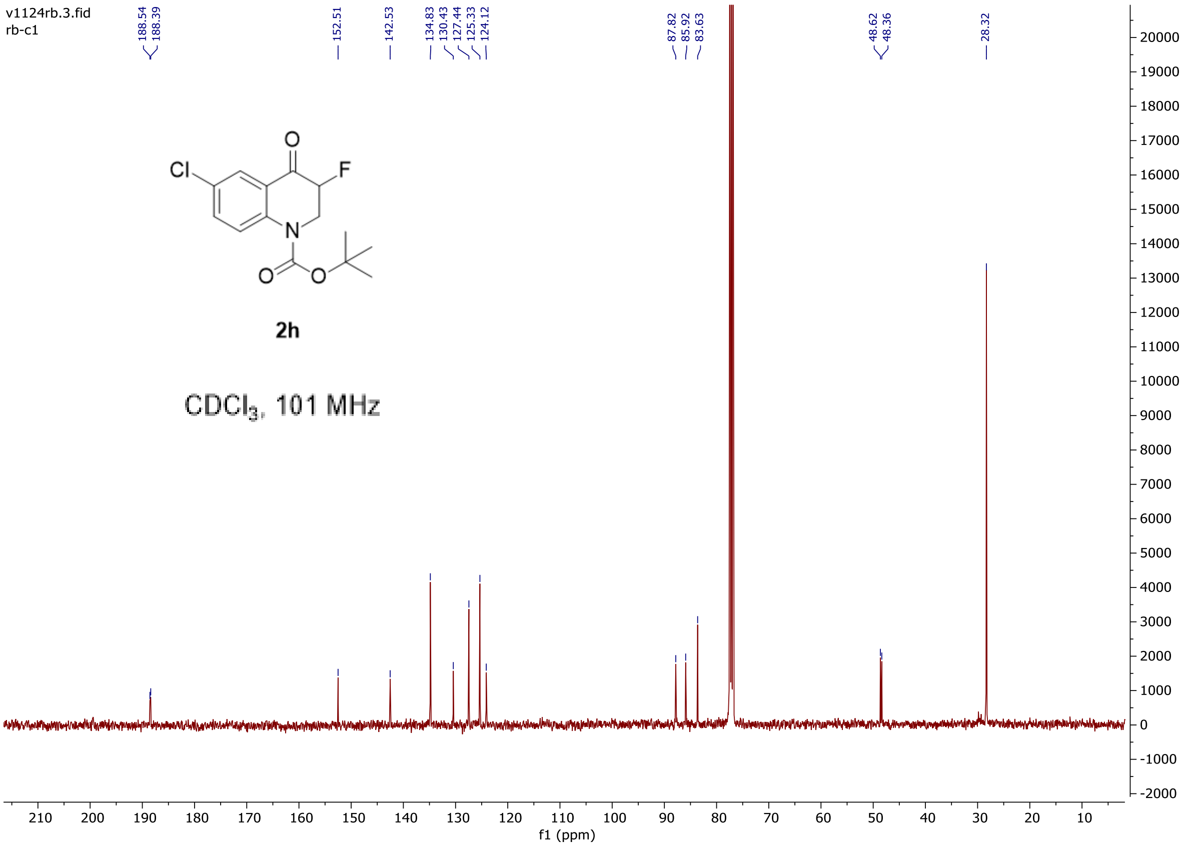
v1124rb.3.fid
rb-c1
188.54
188.39
152.51
142.53
134.83
130.43
127.44
125.33
124.12
87.82
85.92
83.63
48.62
48.36
28.32
Cl
O
F
N
O
O
2h
CDCl3, 101 MHz
f1 (ppm)

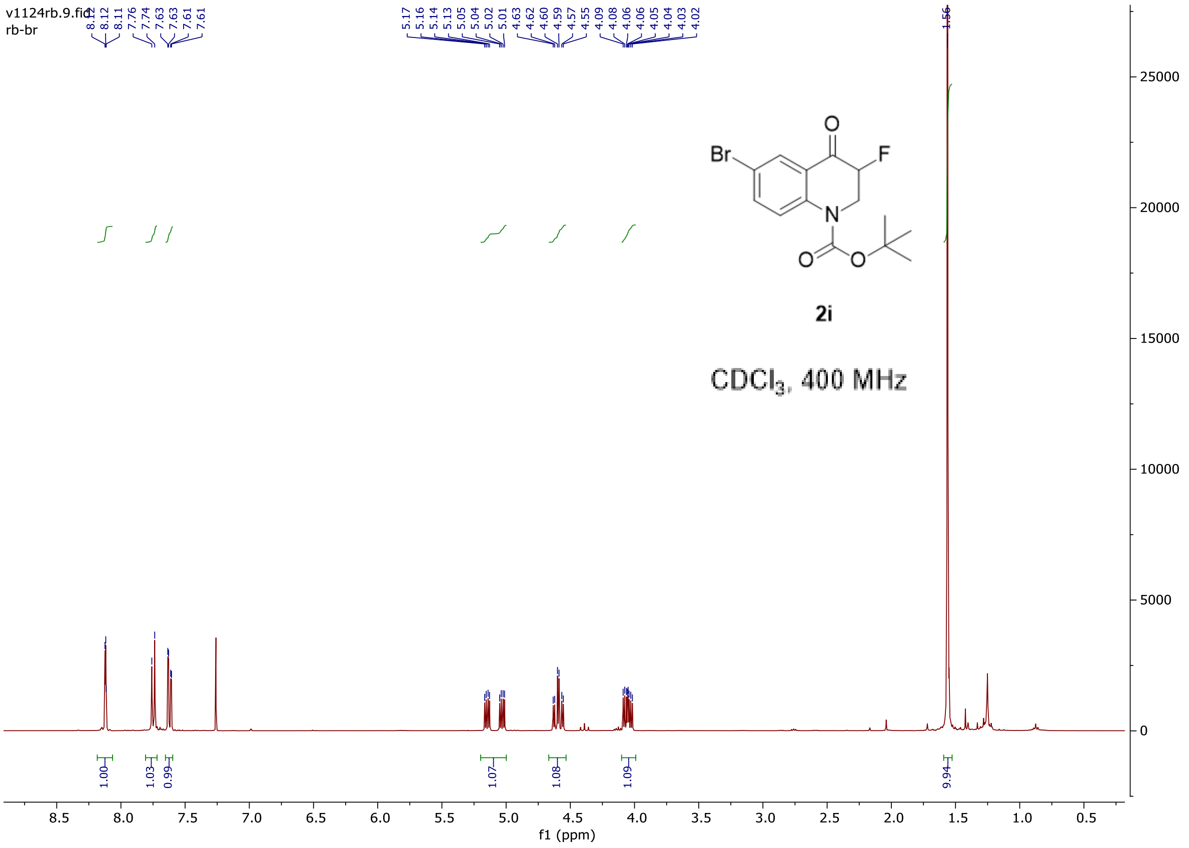
v1124rb.9.fid
rb-br
8.12
8.12
8.11
7.76
7.74
7.63
7.63
7.61
7.61
5.17
5.16
5.14
5.13
5.05
5.04
5.02
5.01
4.63
4.62
4.60
4.59
4.57
4.55
4.09
4.08
4.06
4.06
4.05
4.04
4.03
4.02
1.56
Br
O
F
N
O
O
2i
CDCl3, 400 MHz
1.00
1.03
0.99
1.07
1.08
1.09
9.94
f1 (ppm)

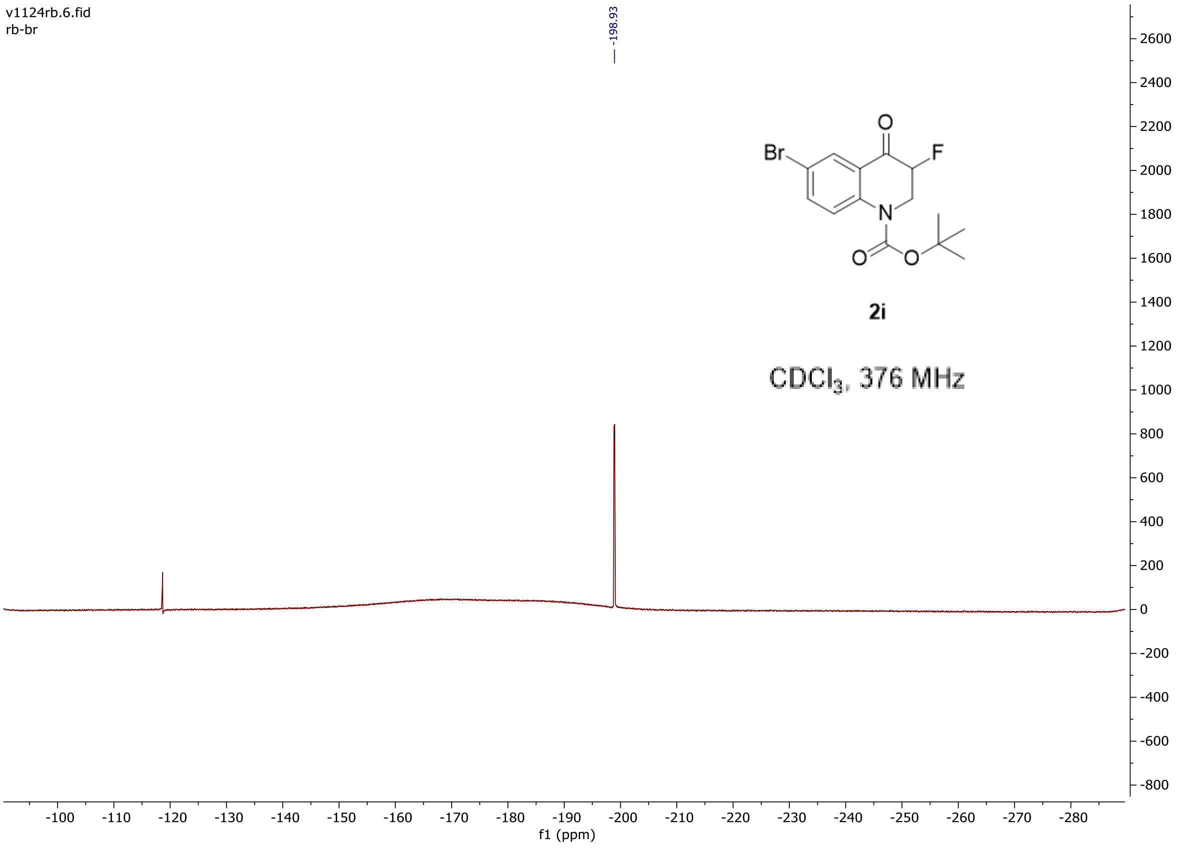
v1124rb.6.fid
rb-br
-198.93
Br
O
F
N
O
O
2i
CDCl3, 376 MHz
f1 (ppm)

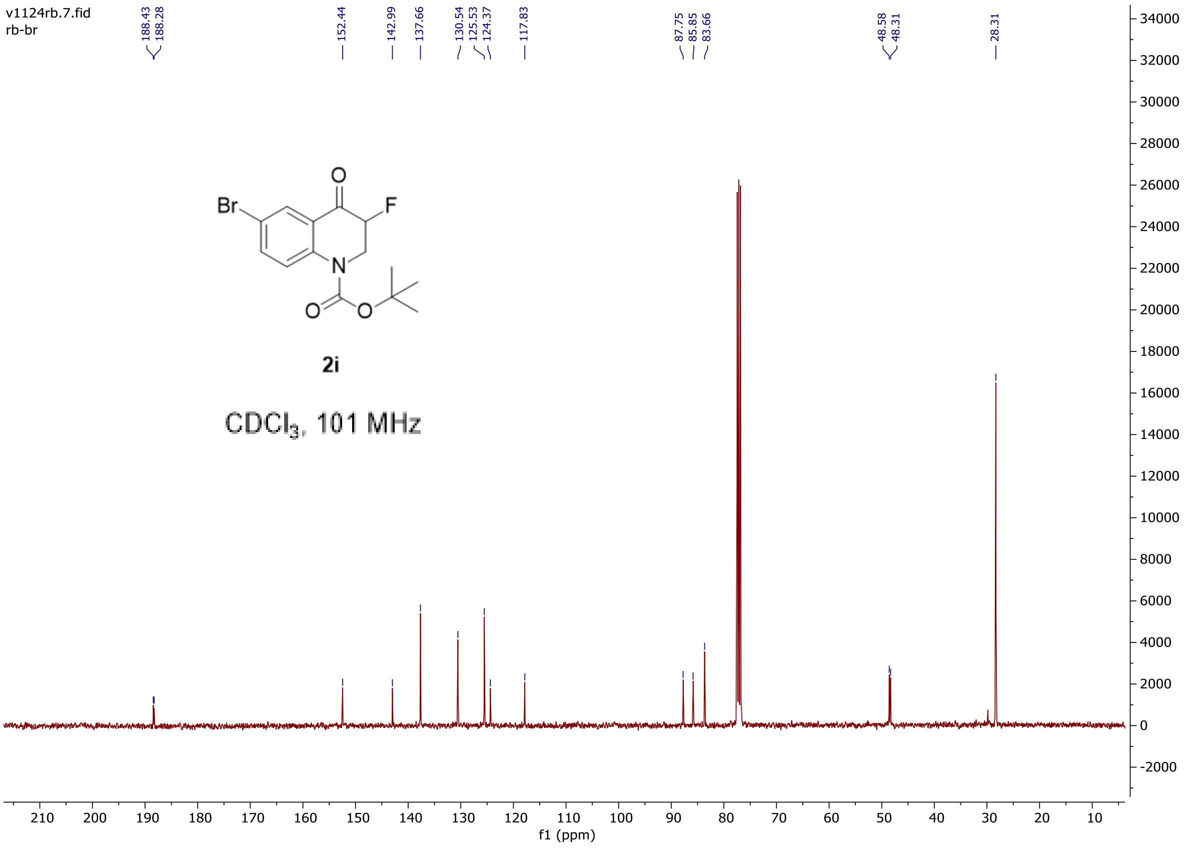
v1124rb.7.fid
rb-br
188.43
188.28
152.44
142.99
137.66
130.54
125.53
124.37
117.83
87.75
85.85
83.66
48.58
48.31
28.31
Br
O
F
N
O
O
2i
CDCl3, 101 MHz
f1 (ppm)

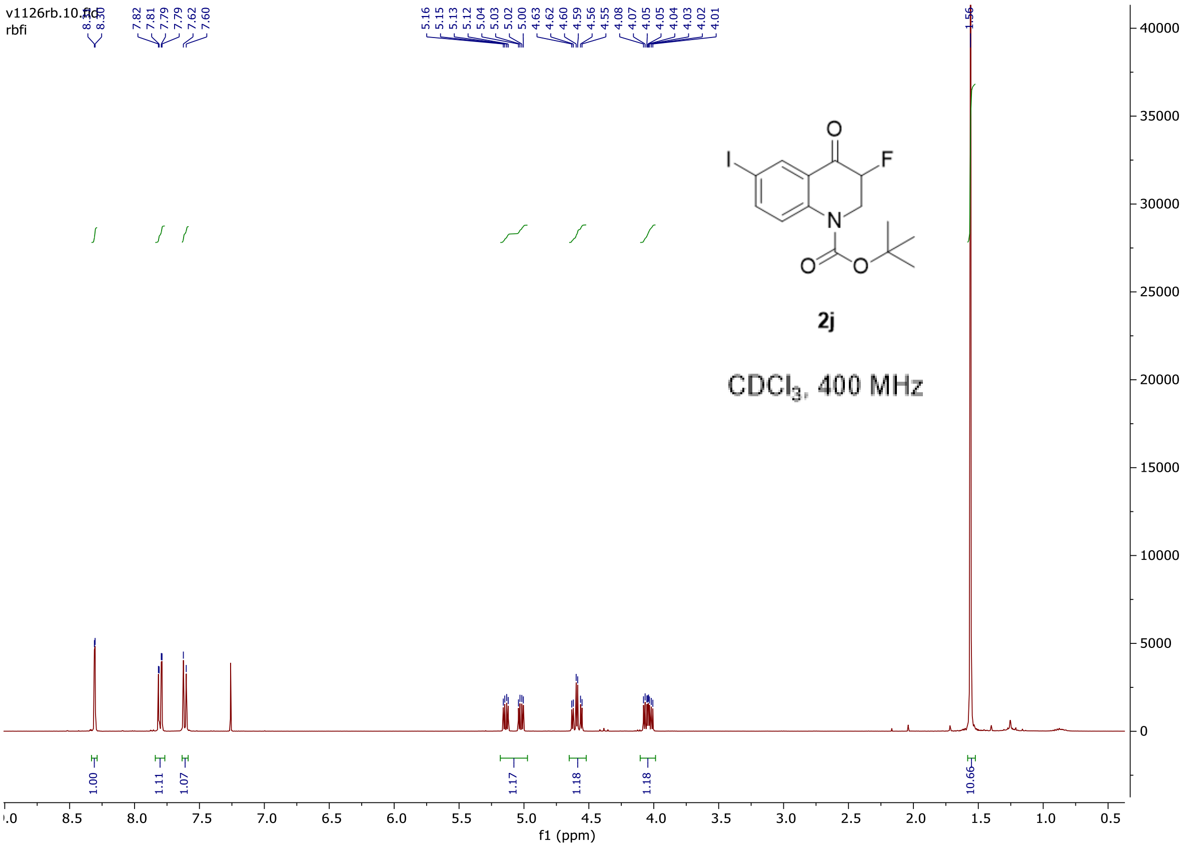
v1126rb.10.fid
rbfi
7.82
7.81
7.79
7.79
7.62
7.60
5.16
5.15
5.13
5.12
5.04
5.03
5.02
5.00
4.63
4.62
4.60
4.59
4.56
4.55
4.08
4.07
4.05
4.05
4.04
4.03
4.02
4.01
1.56
O
I
F
N
O
O
2j
CDCl3, 400 MHz
1.00
1.11
1.07
1.17
1.18
1.18
10.66
f1 (ppm)

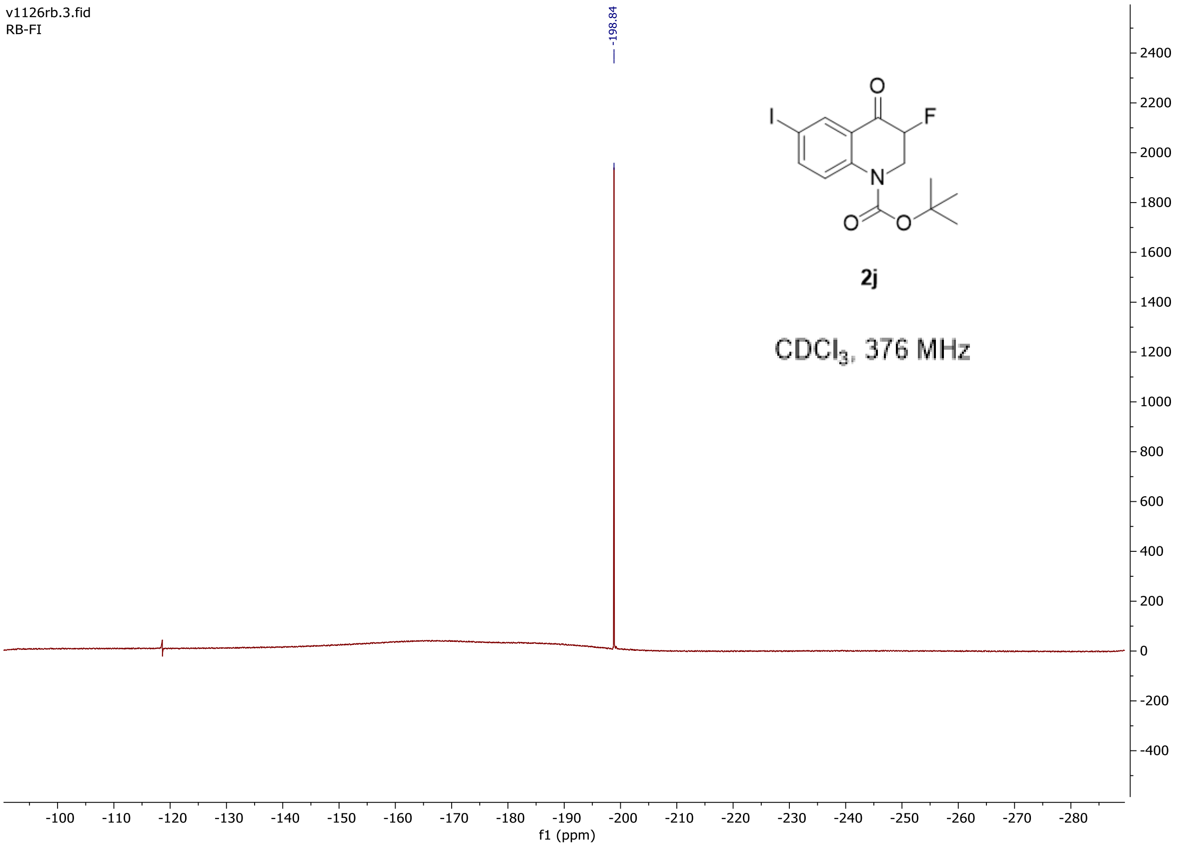
v1126rb.3.fid
RB-FI
-198.84
O
I
F
N
O
O
2j
CDCl3, 376 MHz
f1 (ppm)

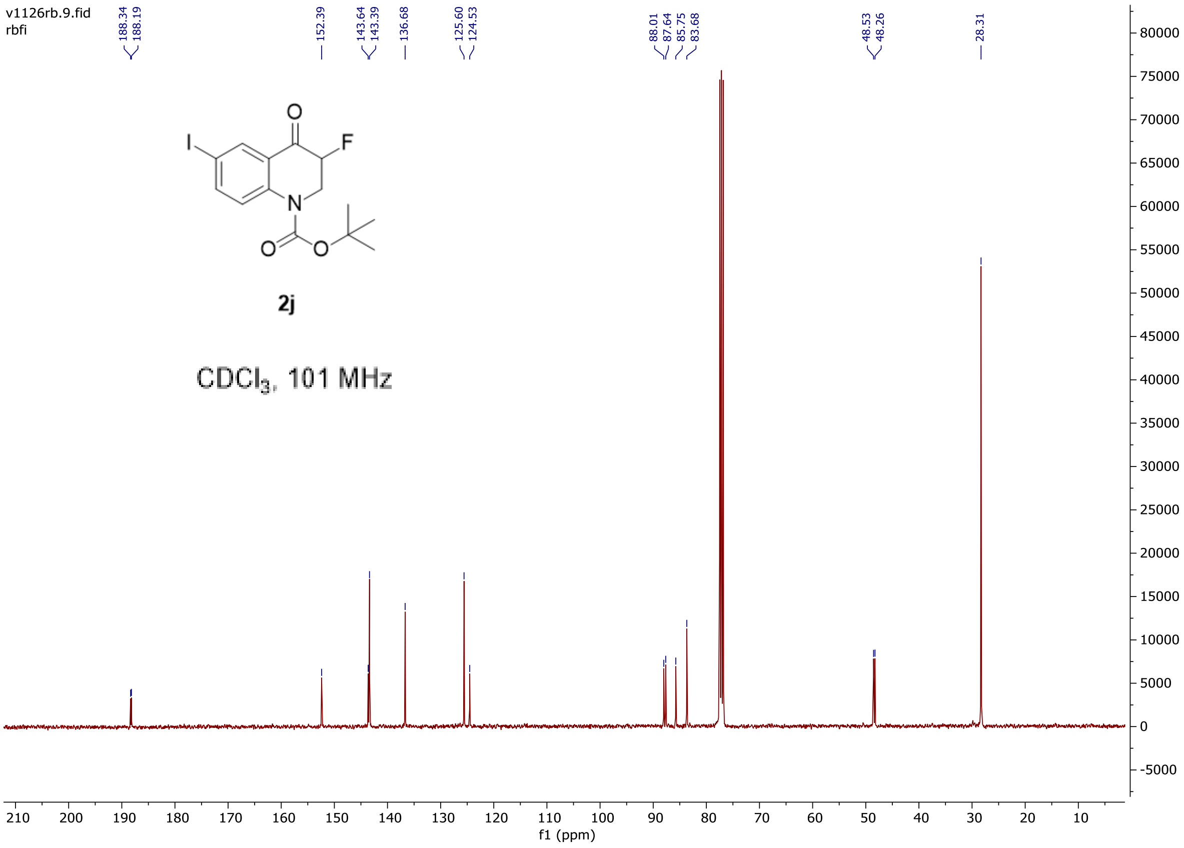
v1126rb.9.fid
rbfi
188.34
188.19
152.39
143.64
143.39
136.68
125.60
124.53
88.01
87.64
85.75
83.68
48.53
48.26
28.31
2j
CDCl3, 101 MHz
f1 (ppm)

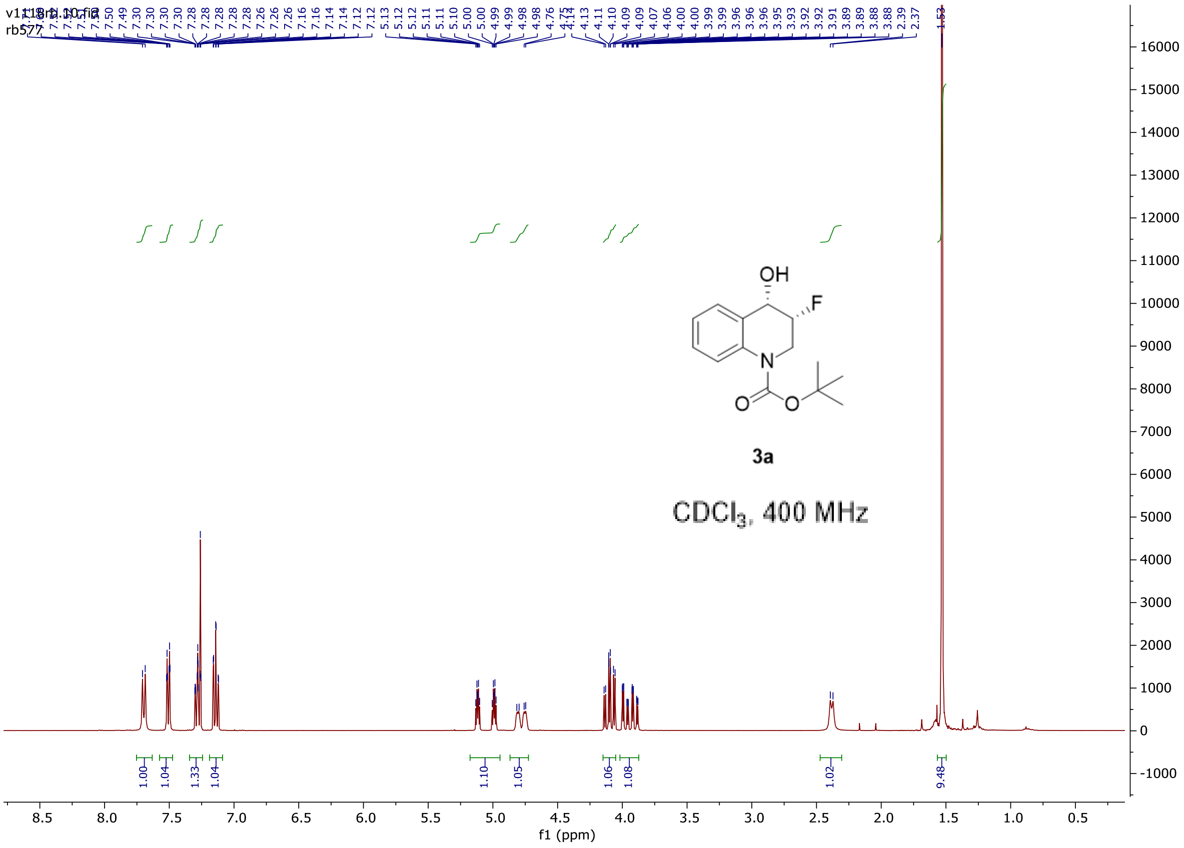
rb577
3a
CDCl3, 400 MHz
1.00
1.04
1.33
1.04
1.10
1.05
1.06
1.08
1.02
9.48
f1 (ppm)

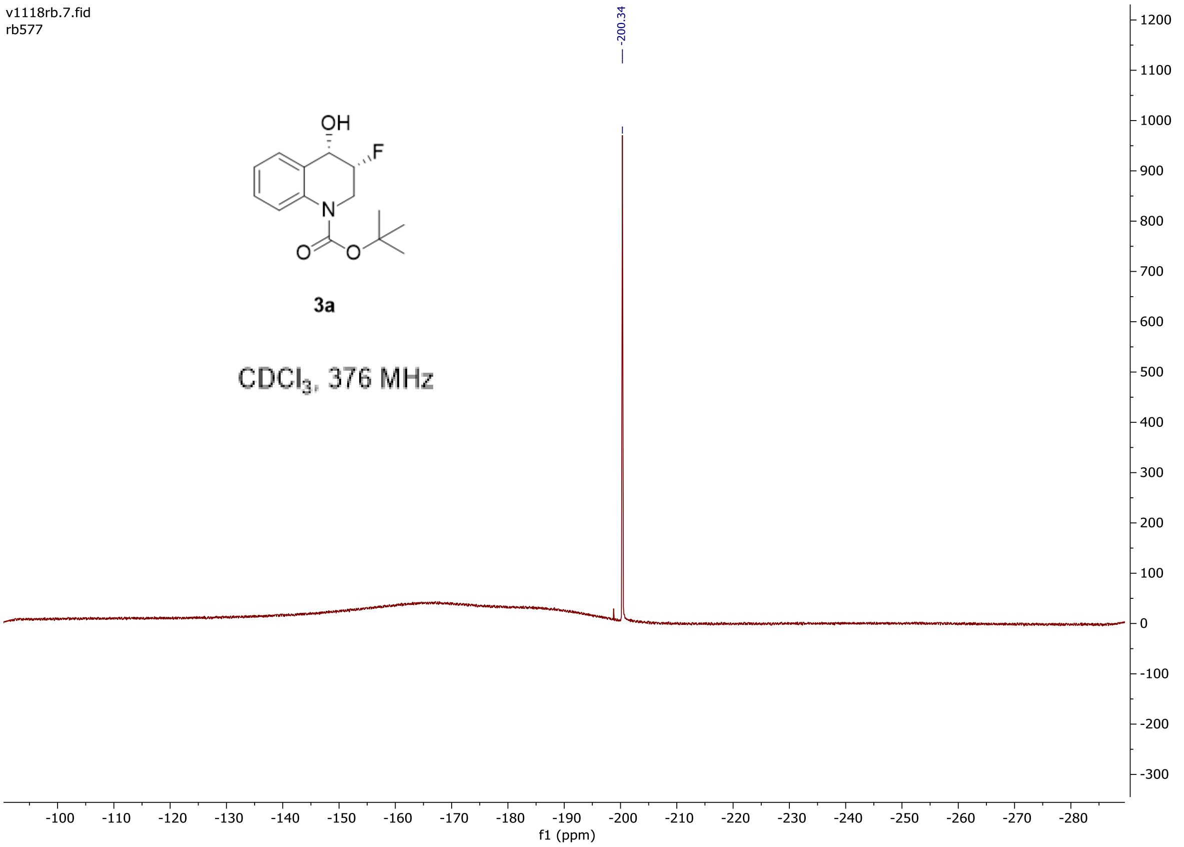
v1118rb.7.fid
rb577
-200.34
3a
CDCl3, 376 MHz
f1 (ppm)

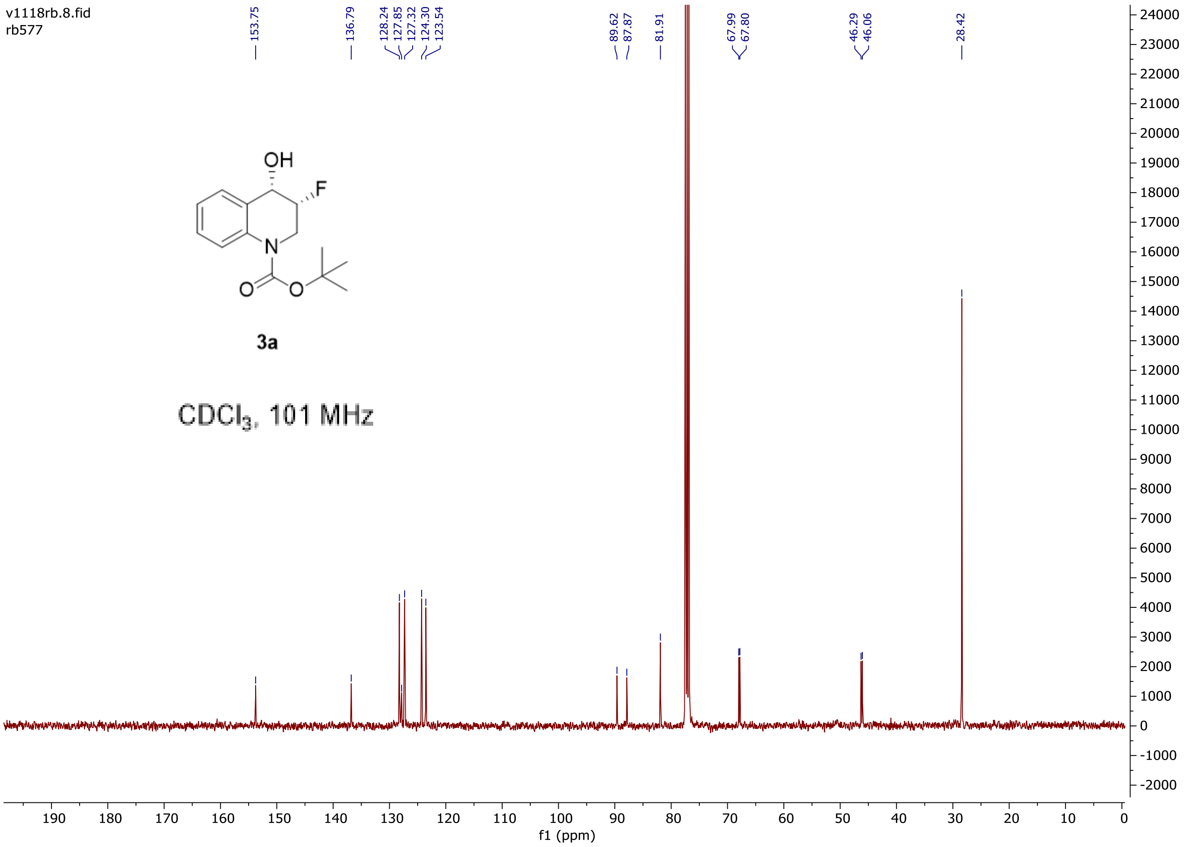
v1118rb.8.fid
rb577
153.75
136.79
128.24
127.85
127.32
124.30
123.54
89.62
87.87
81.91
67.99
67.80
46.29
46.06
28.42
OH
F
N
O
O
3a
CDCl3, 101 MHz
f1 (ppm)

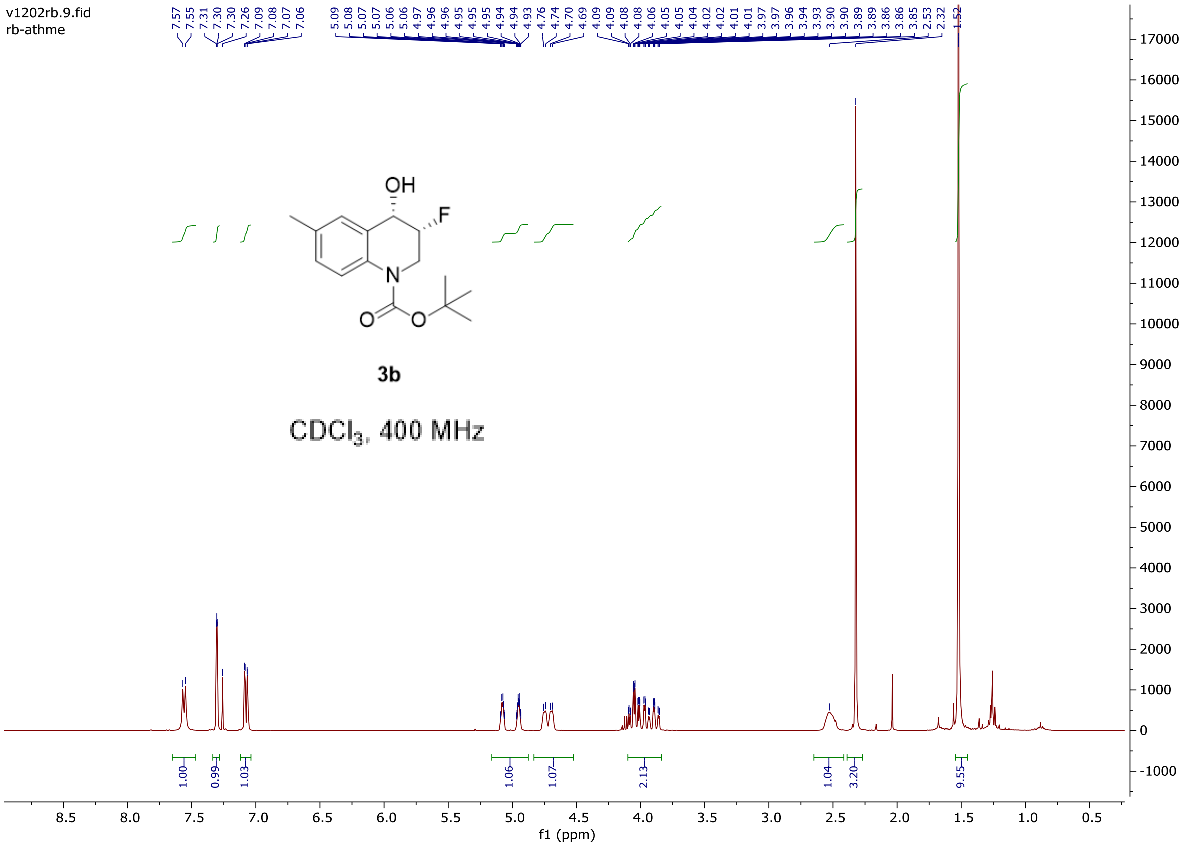
v1202rb.9.fid
rb-athme
3b
CDCl$_3$, 400 MHz
f1 (ppm)

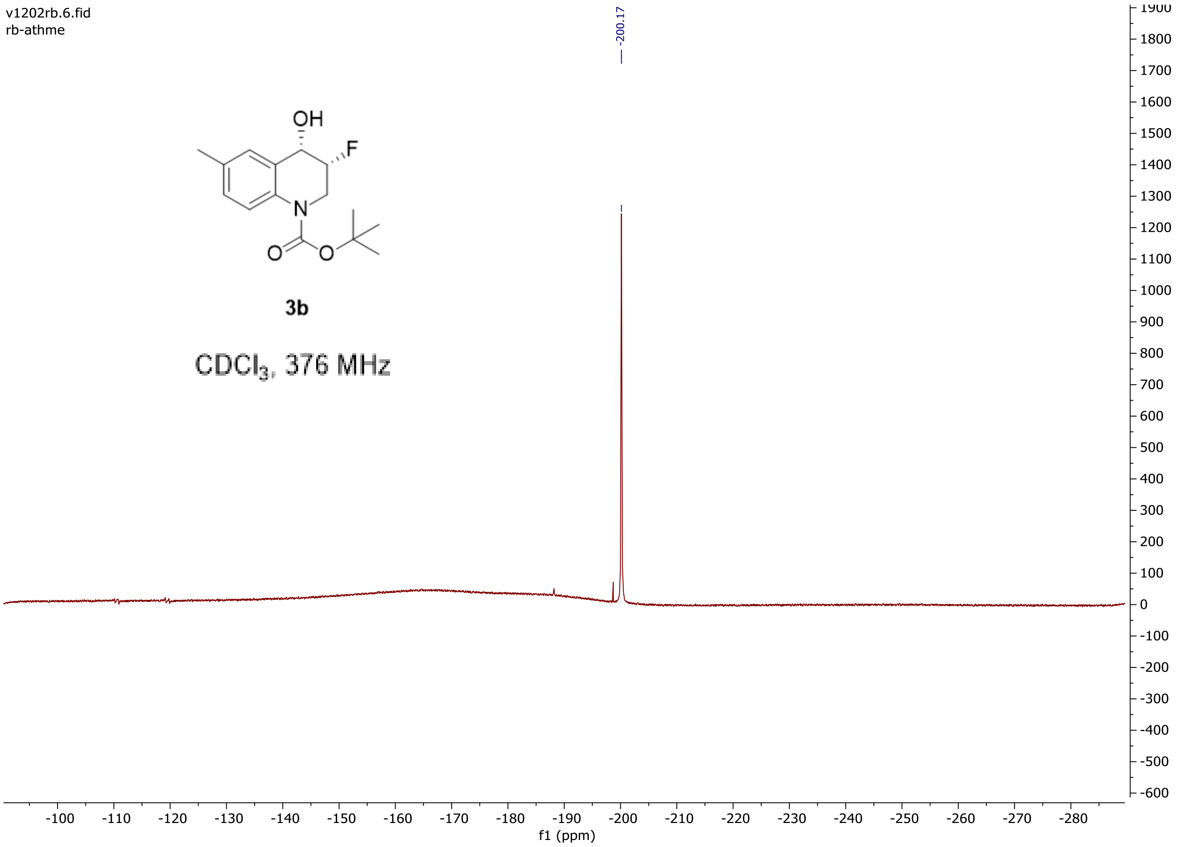
v1202rb.6.fid
rb-athme
-200.17
3b
CDCl$_3$, 376 MHz
f1 (ppm)

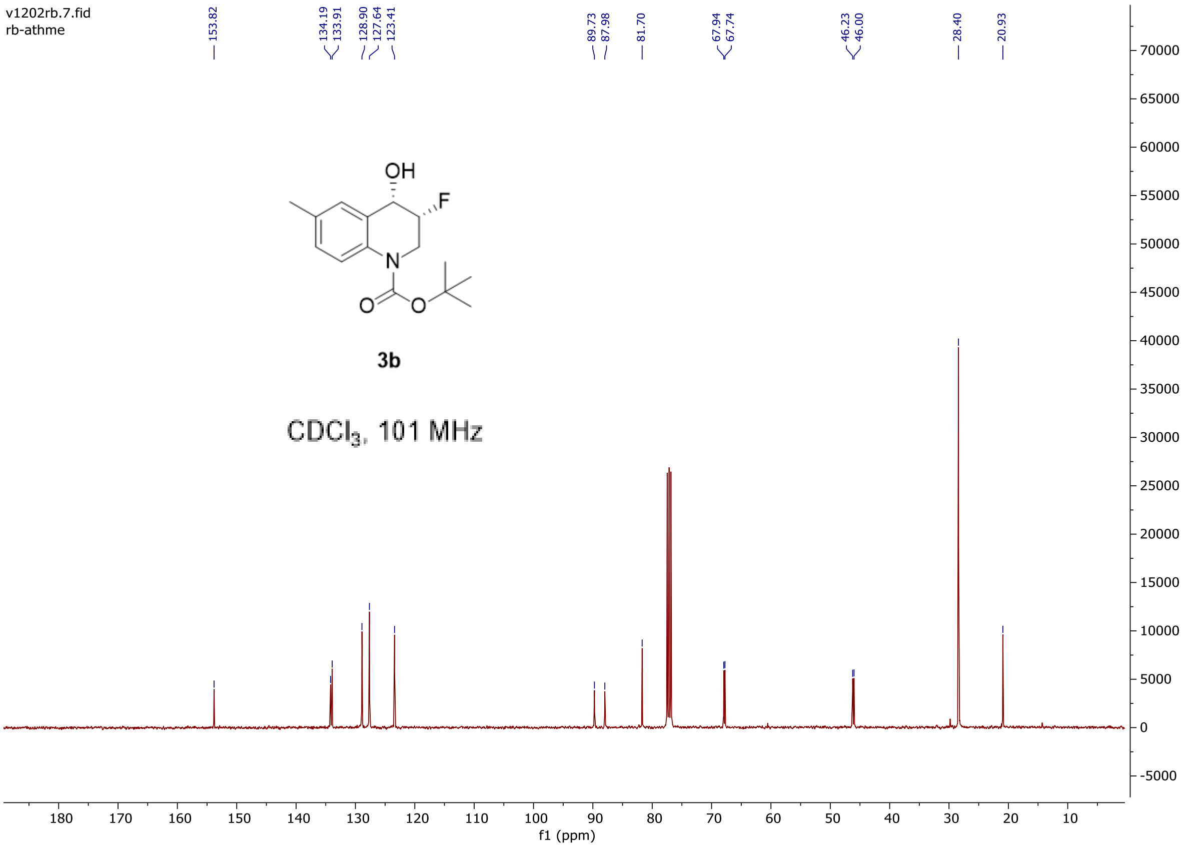
v1202rb.7.fid
rb-athme
153.82
134.19
133.91
128.90
127.64
123.41
89.73
87.98
81.70
67.94
67.74
46.23
46.00
28.40
20.93
OH
F
N
O
O
3b
CDCl3, 101 MHz
f1 (ppm)

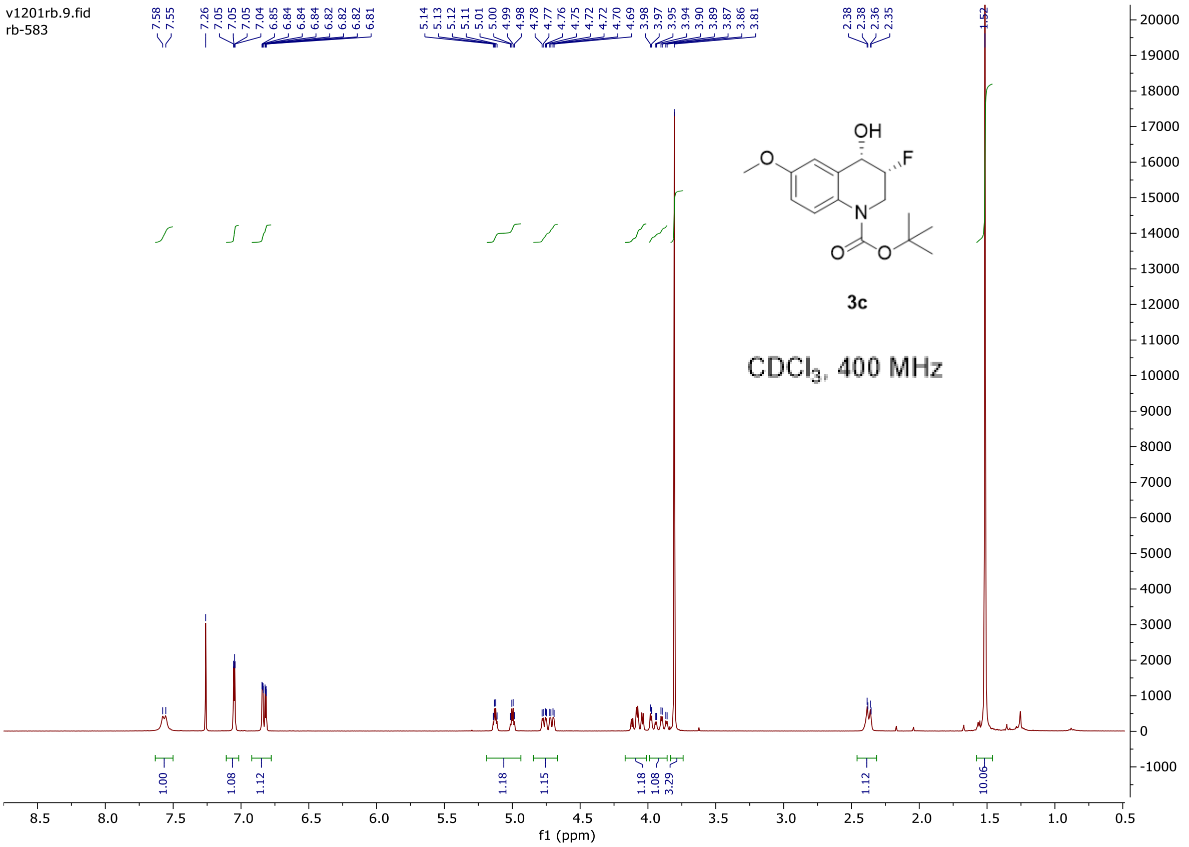
v1201rb.9.fid
rb-583
3c
CDCl3, 400 MHz
f1 (ppm)

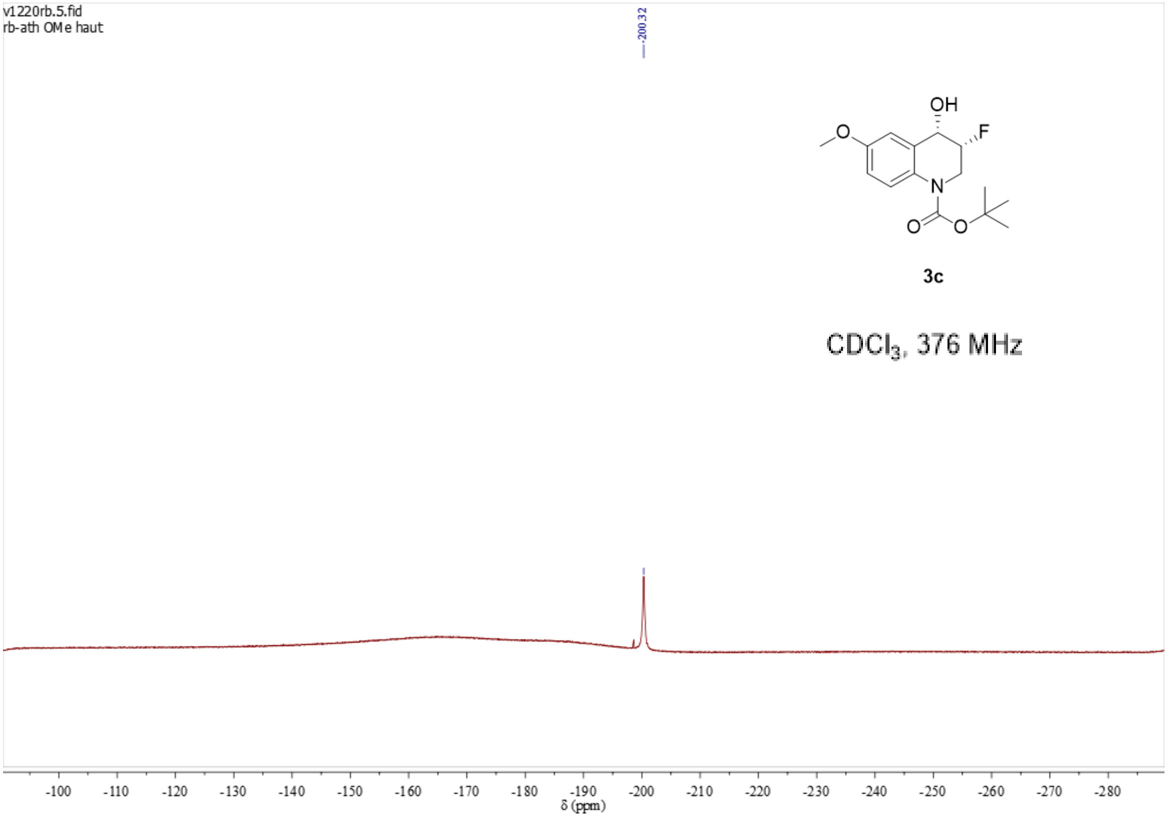
v1220rb.5.fid
rb-ath OMe haut
-200.32
3c
CDCl3, 376 MHz
δ (ppm)

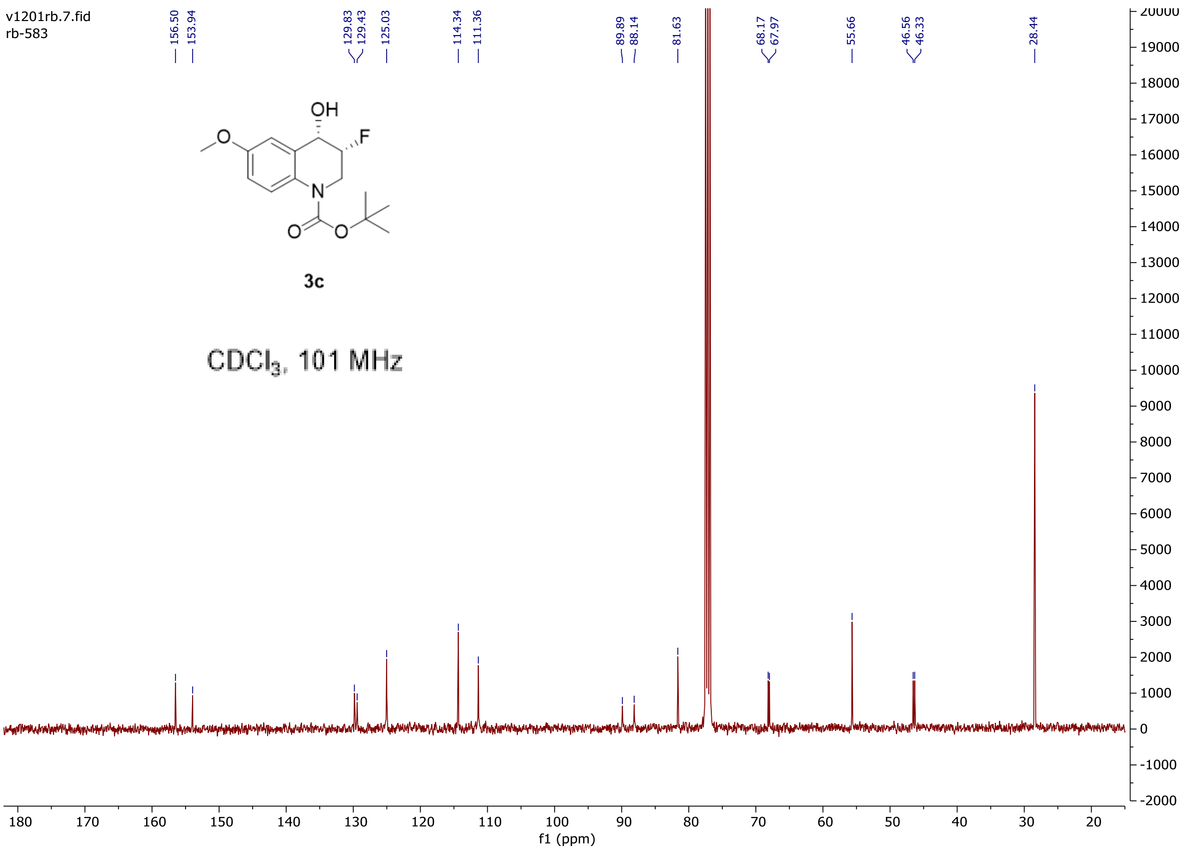
v1201rb.7.fid
rb-583
156.50
153.94
129.83
129.43
125.03
114.34
111.36
89.89
88.14
81.63
68.17
67.97
55.66
46.56
46.33
28.44
OH
F
O
N
O
O
3c
CDCl3, 101 MHz
f1 (ppm)

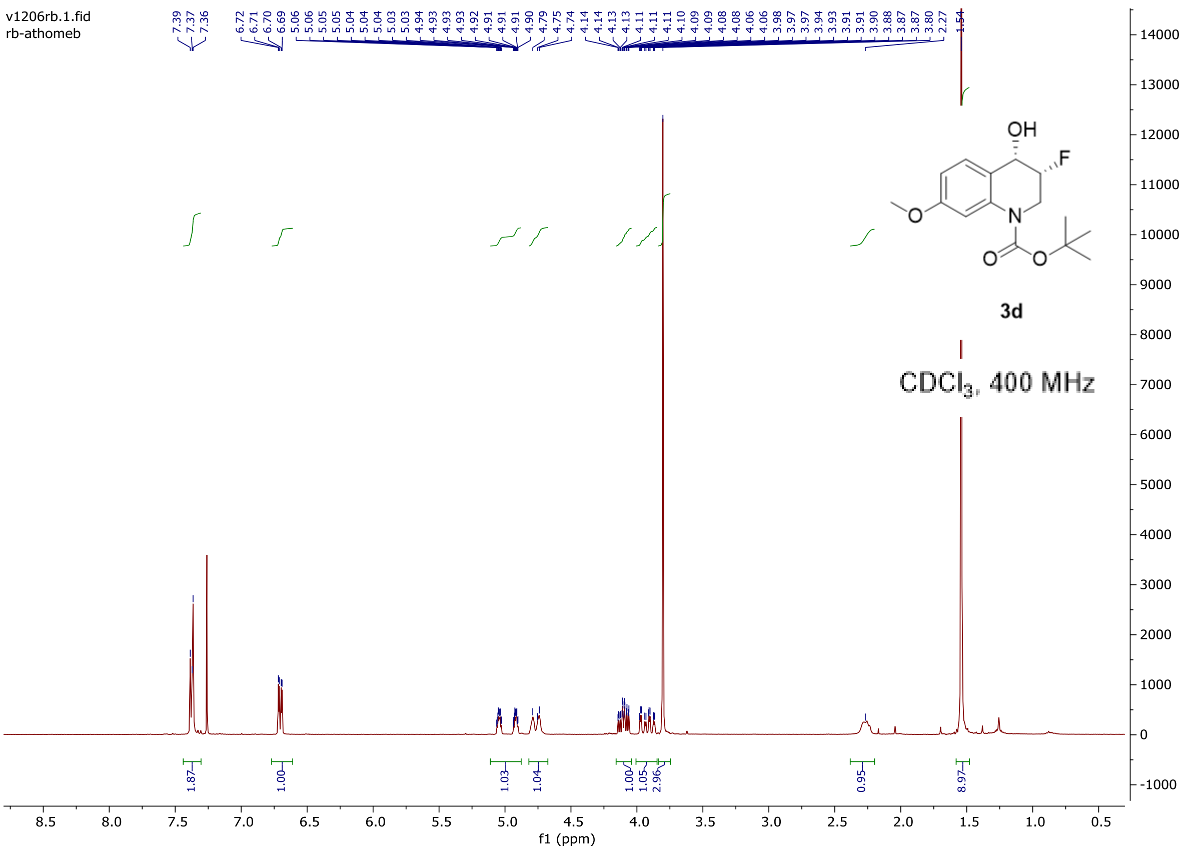
v1206rb.1.fid
rb-athomeb
3d
CDCl3, 400 MHz
f1 (ppm)

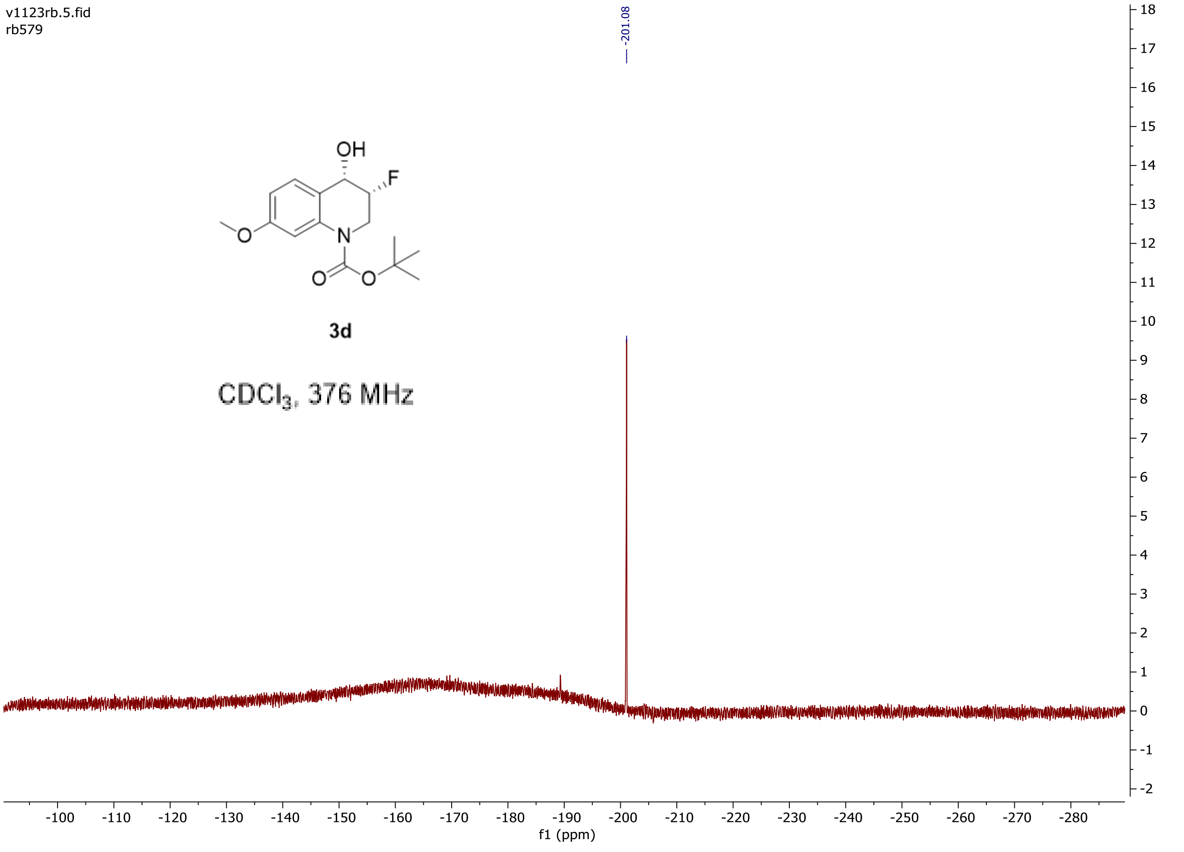
v1123rb.5.fid
rb579
-201.08
3d
CDCl3, 376 MHz
f1 (ppm)

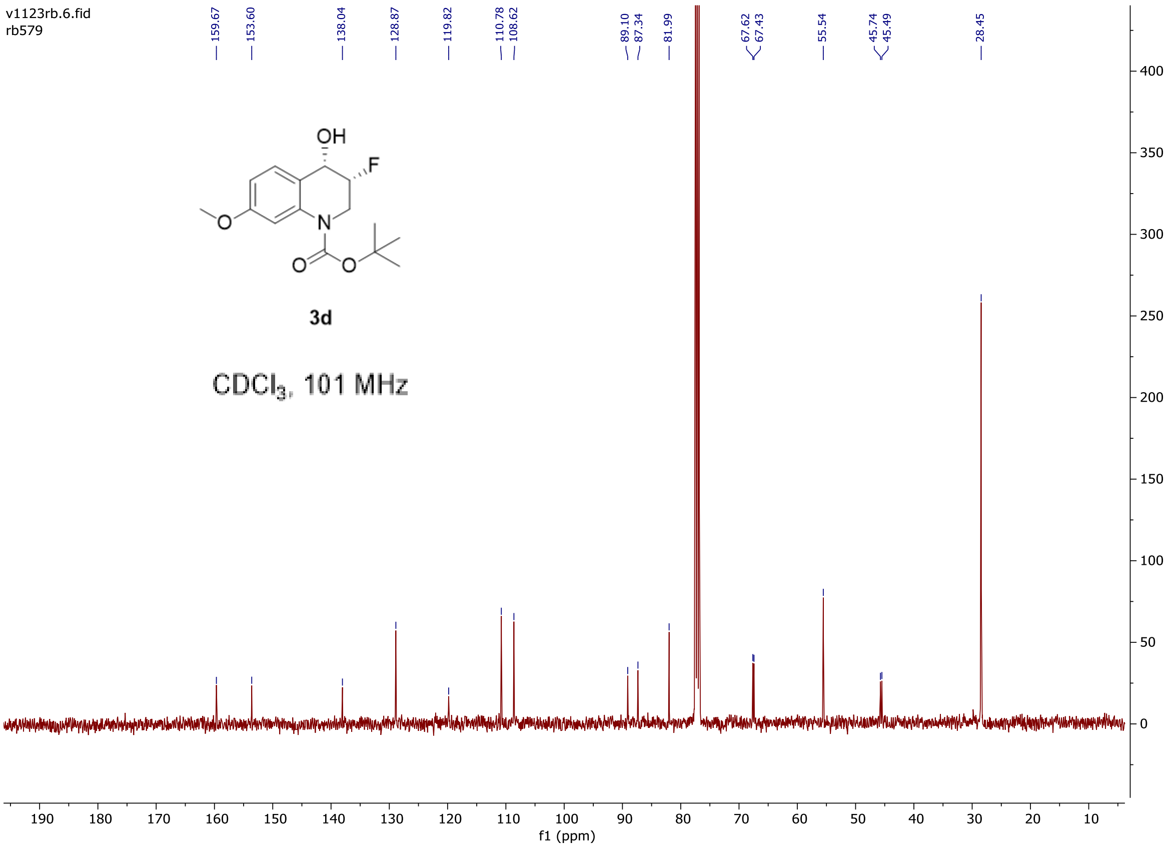
v1123rb.6.fid
rb579
159.67
153.60
138.04
128.87
119.82
110.78
108.62
89.10
87.34
81.99
67.62
67.43
55.54
45.74
45.49
28.45
OH
F
O
N
O
O
3d
CDCl3, 101 MHz
f1 (ppm)

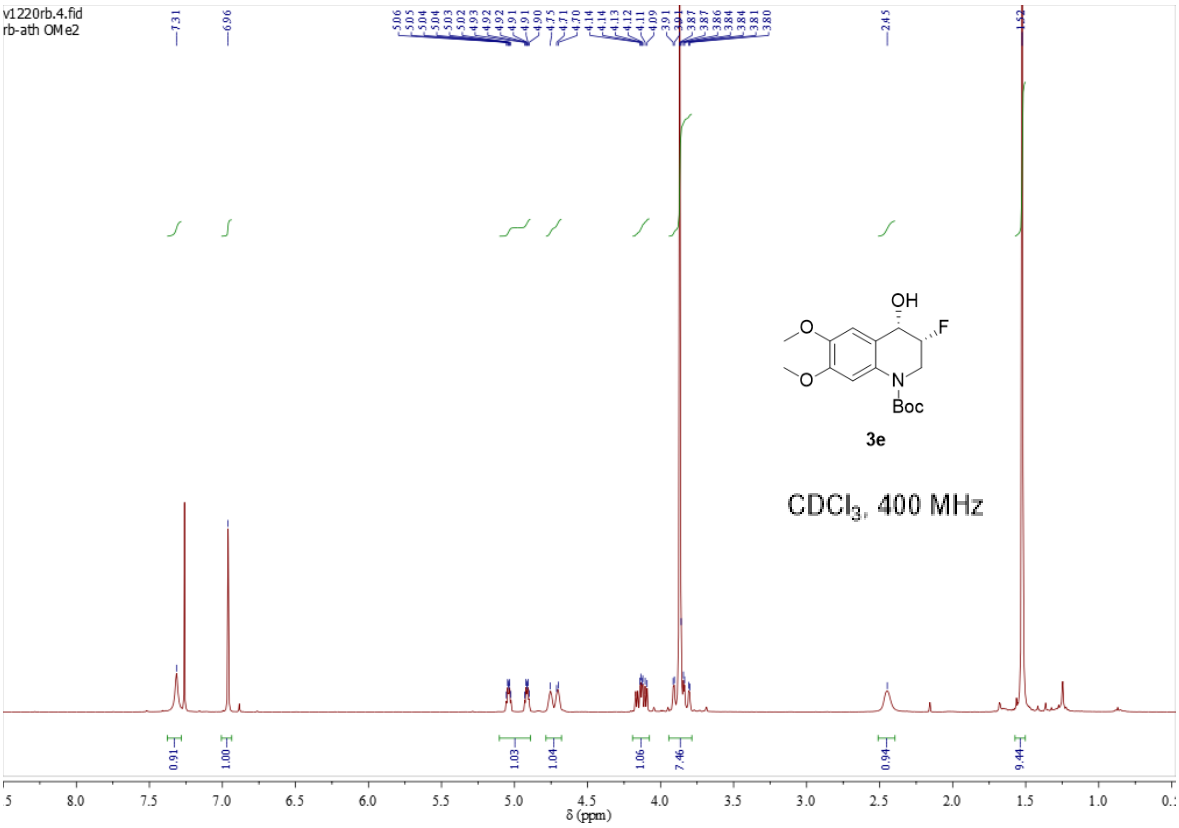
v1220rb.4.fid
rb-ath OMe2
3e
CDCl3, 400 MHz
δ (ppm)

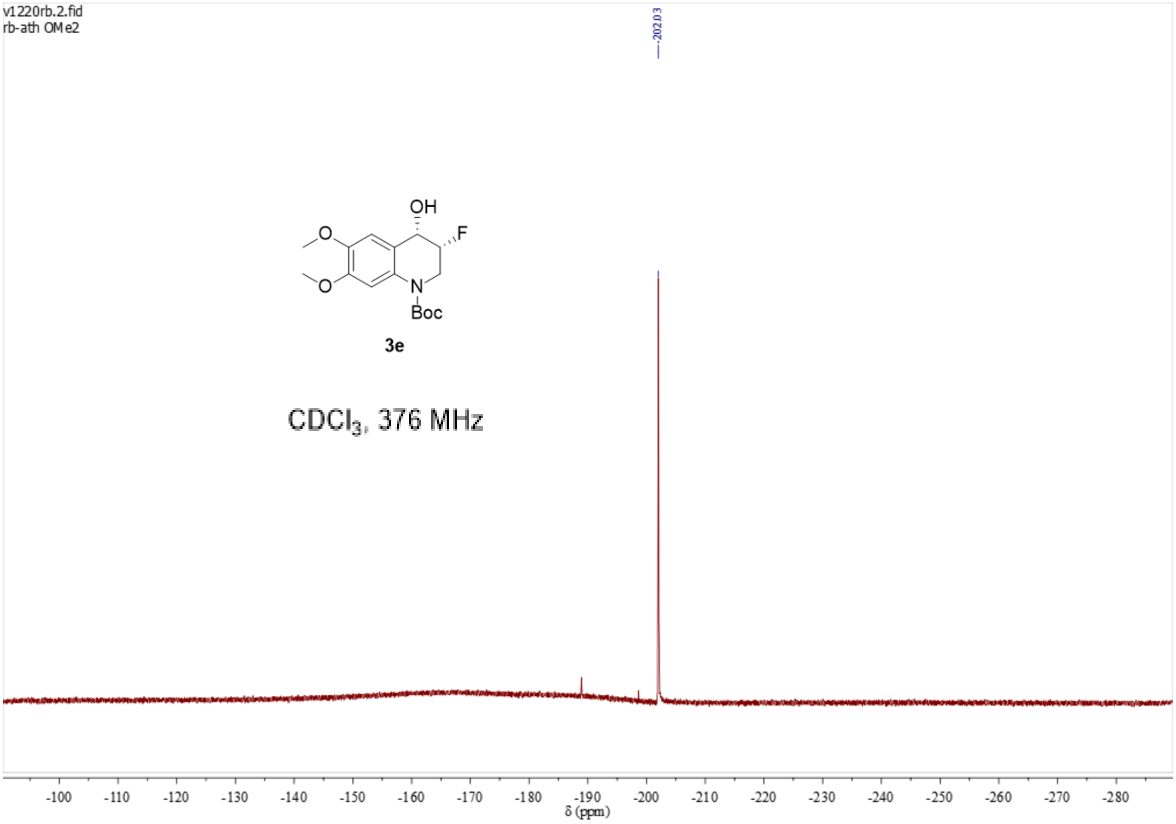
v1220rb.2.fid
rb-ath OMe2
-202.03
3e
CDCl3, 376 MHz
δ (ppm)

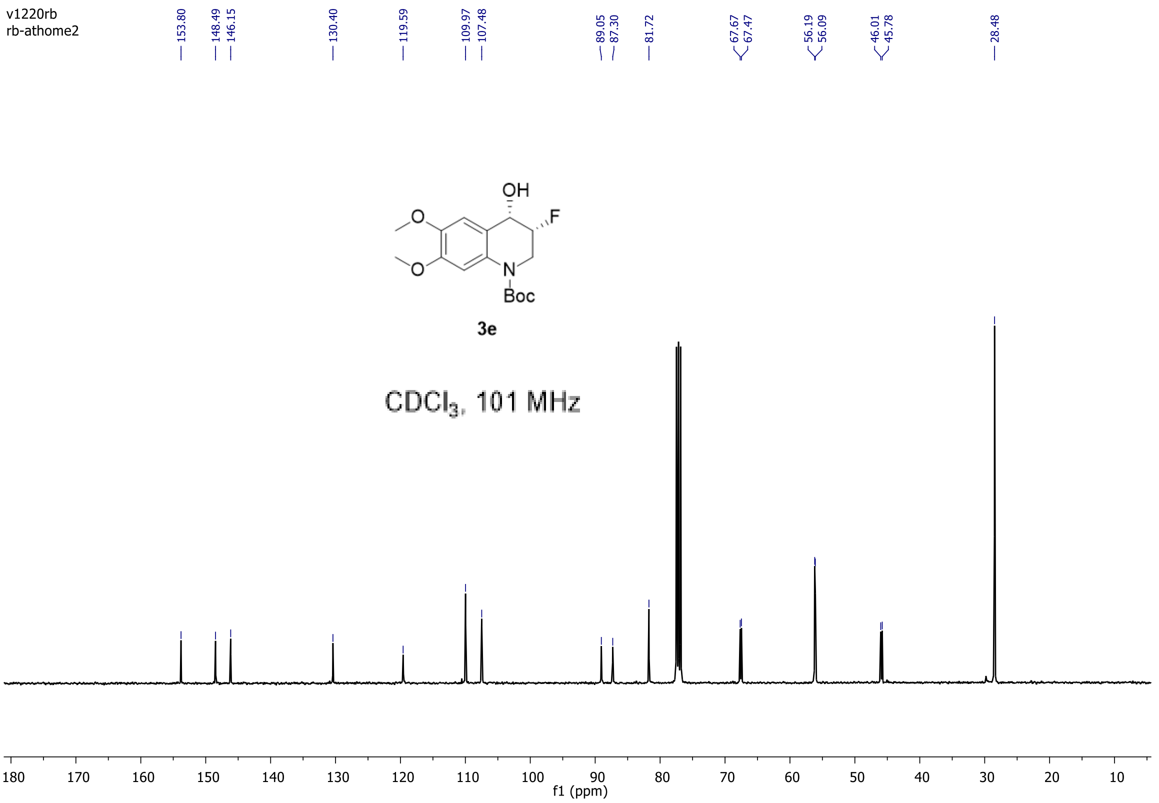

v1220rb
rb-athome2
153.80
148.49
146.15
130.40
119.59
109.97
107.48
89.05
87.30
81.72
67.67
67.47
56.19
56.09
46.01
45.78
28.48
OH
F
O
O
N
Boc
3e
CDCl3, 101 MHz
180
170
160
150
140
130
120
110
100
90
80
70
60
50
40
30
20
10
f1 (ppm)

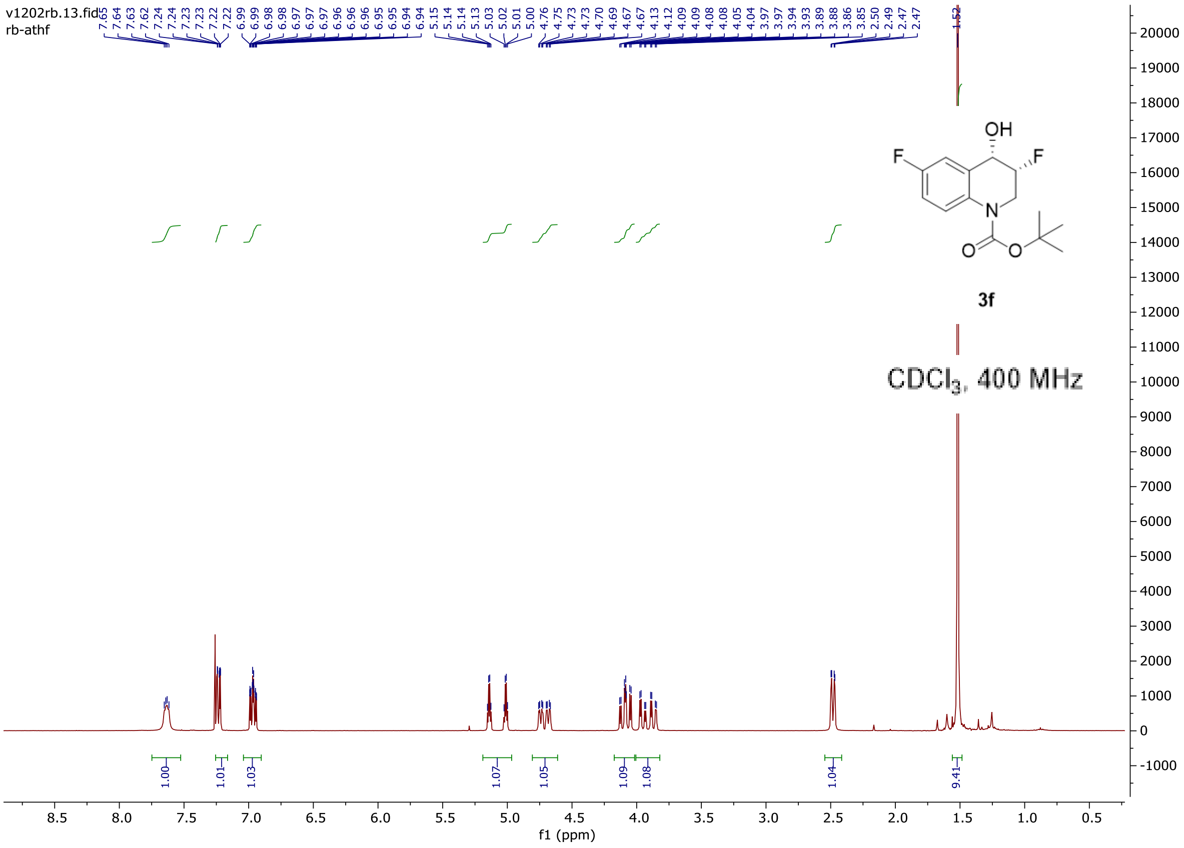
v1202rb.13.fid
rb-athf
3f
CDCl3, 400 MHz
f1 (ppm)

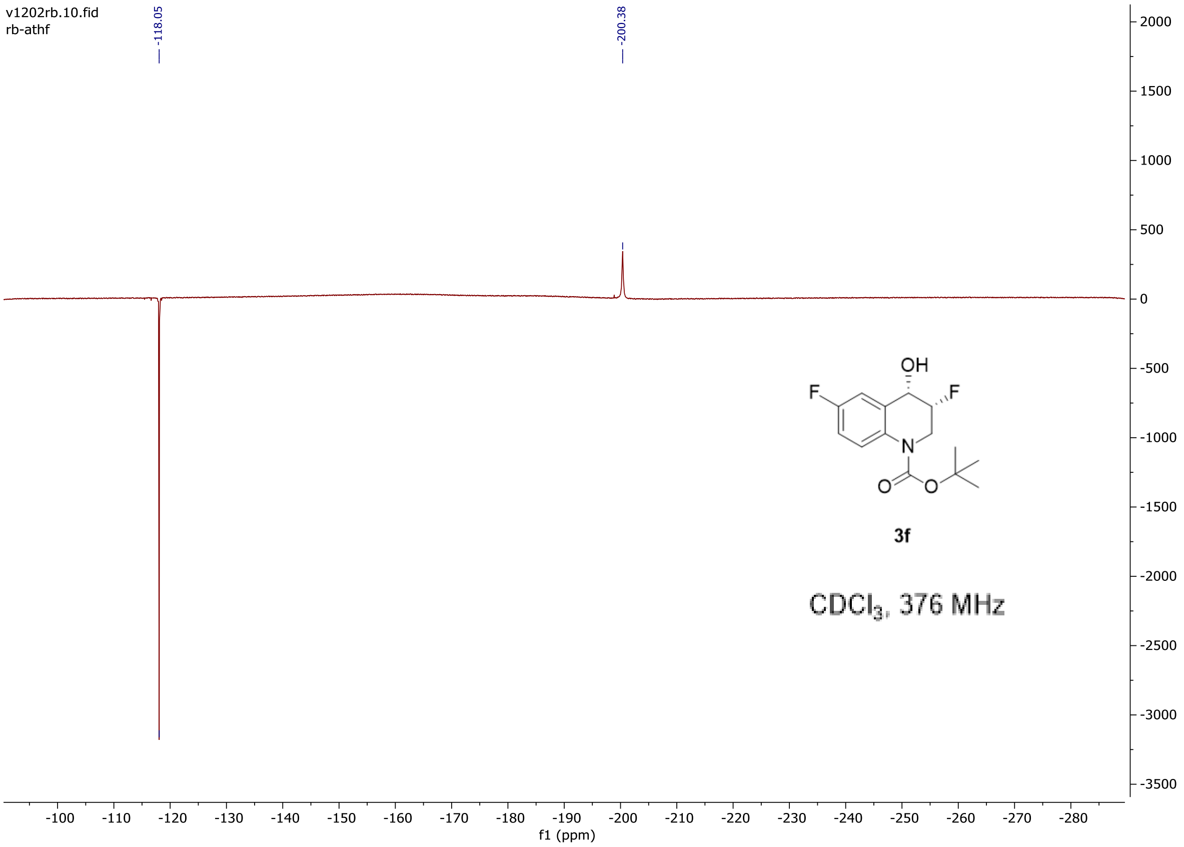
v1202rb.10.fid
rb-athf
-118.05
-200.38
3f
CDCl3, 376 MHz
f1 (ppm)

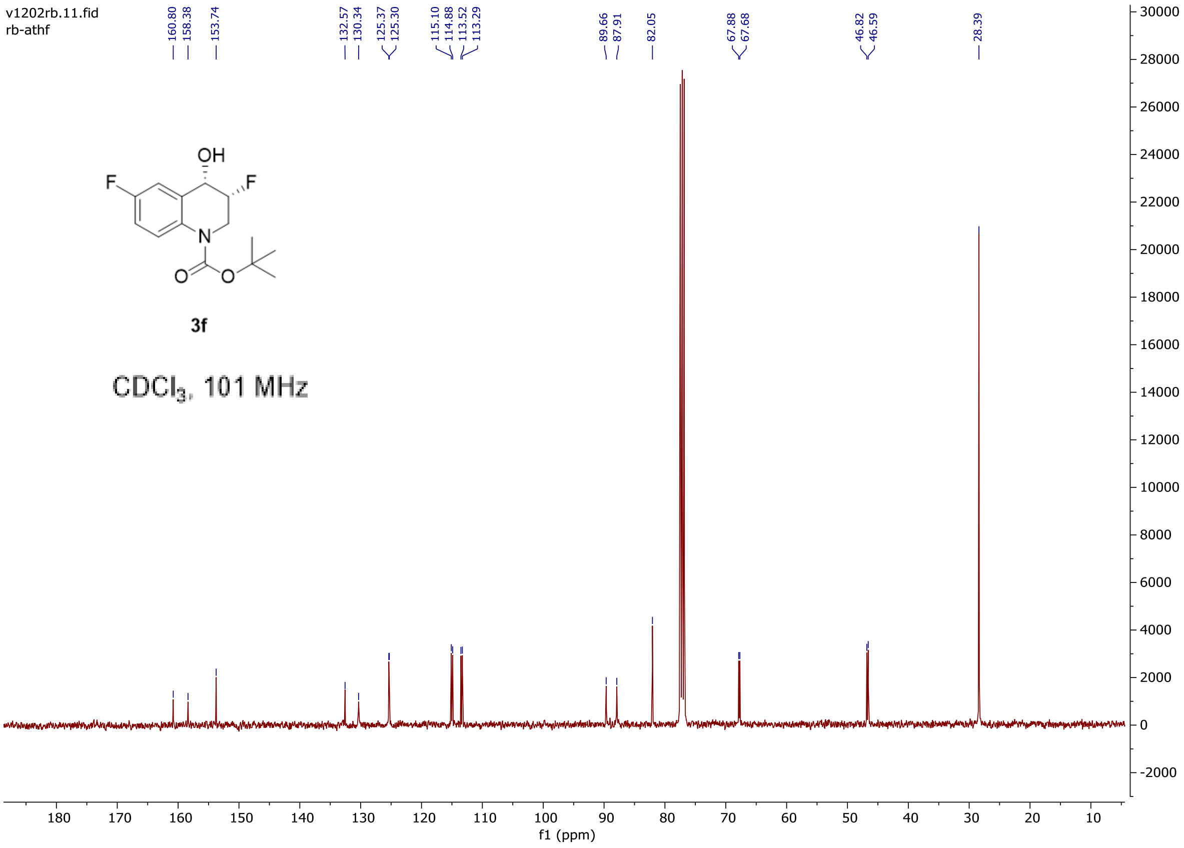

v1202rb.11.fid
rb-athf
160.80
158.38
153.74
132.57
130.34
125.37
125.30
115.10
114.88
113.52
113.29
89.66
87.91
82.05
67.88
67.68
46.82
46.59
28.39
3f
CDCl3, 101 MHz
f1 (ppm)

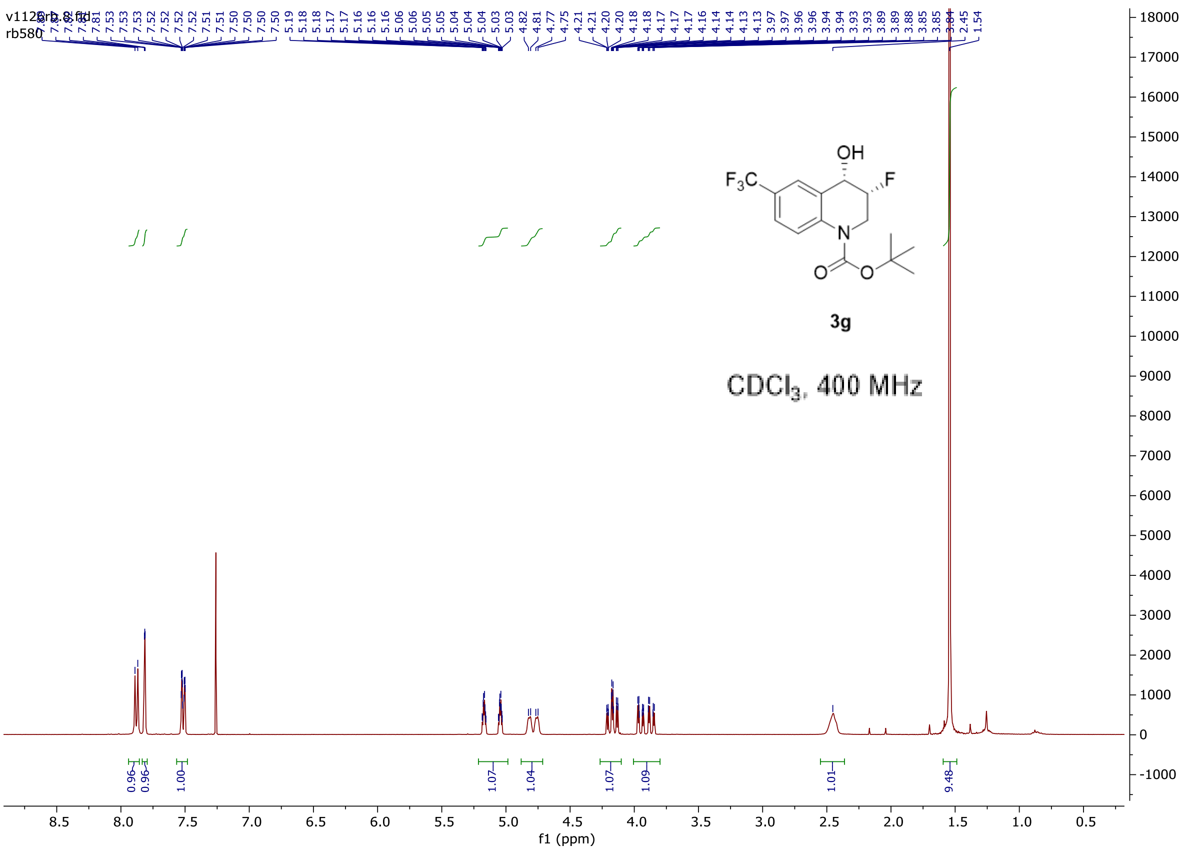
rb580
3g
CDCl3, 400 MHz
0.96
0.96
1.00
1.07
1.04
1.07
1.09
1.01
9.48
f1 (ppm)

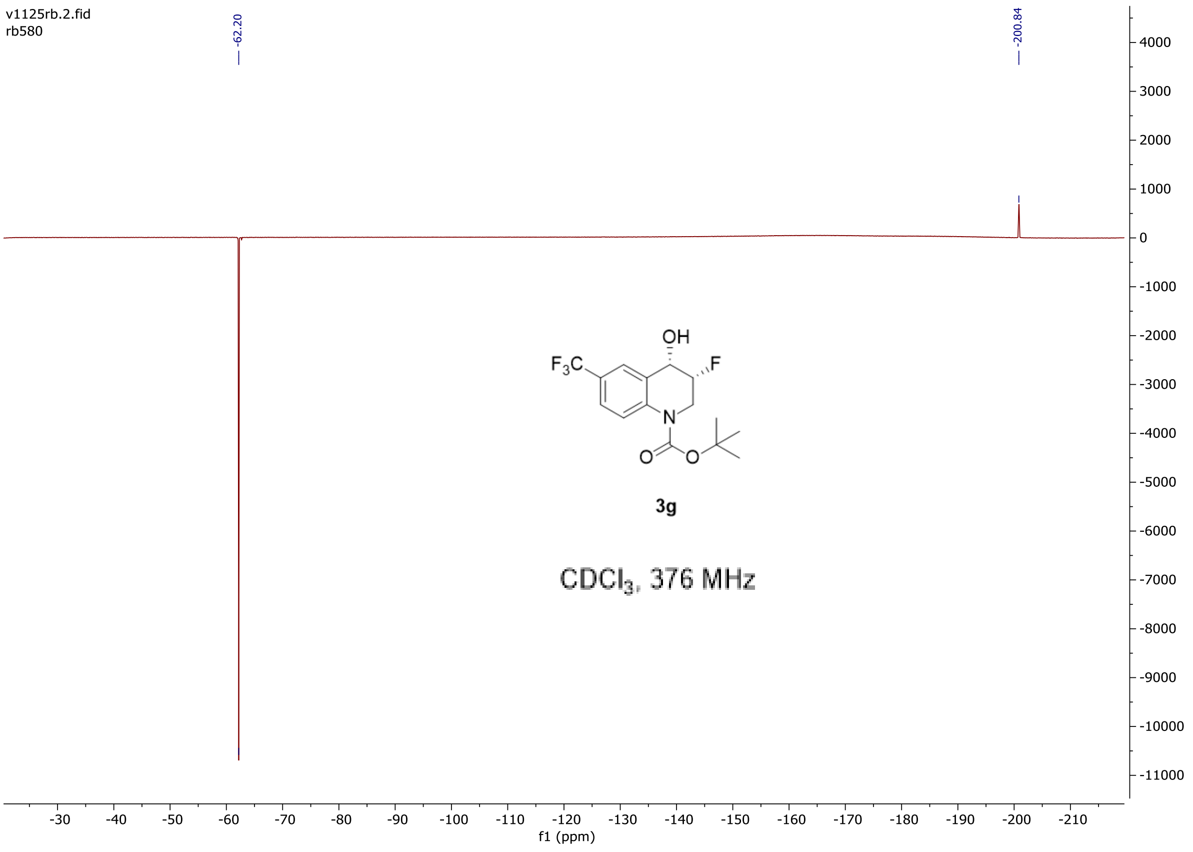
v1125rb.2.fid
rb580
-62.20
-200.84
3g
CDCl3, 376 MHz
f1 (ppm)

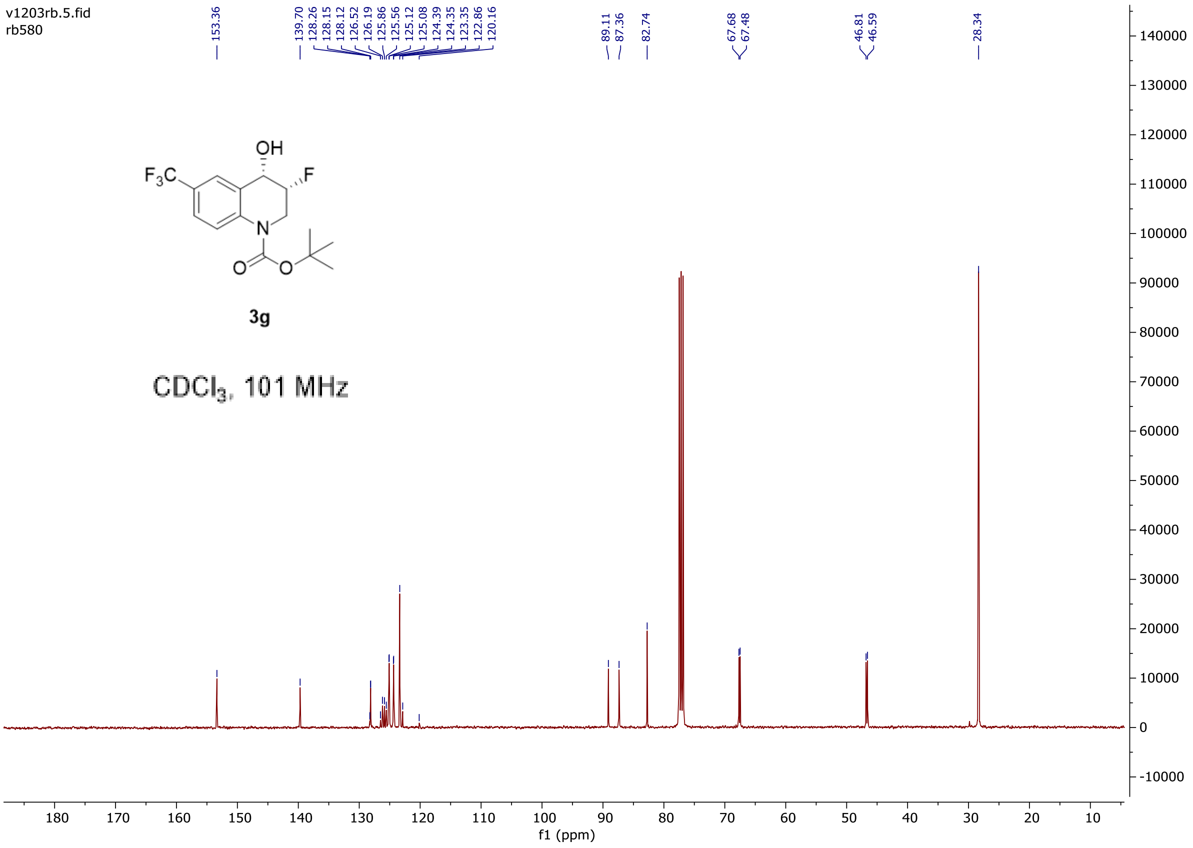
v1203rb.5.fid
rb580
153.36
139.70
128.26
128.15
128.12
126.52
126.19
125.86
125.56
125.12
125.08
124.39
124.35
123.35
122.86
120.16
89.11
87.36
82.74
67.68
67.48
46.81
46.59
28.34
3g
CDCl3, 101 MHz
f1 (ppm)

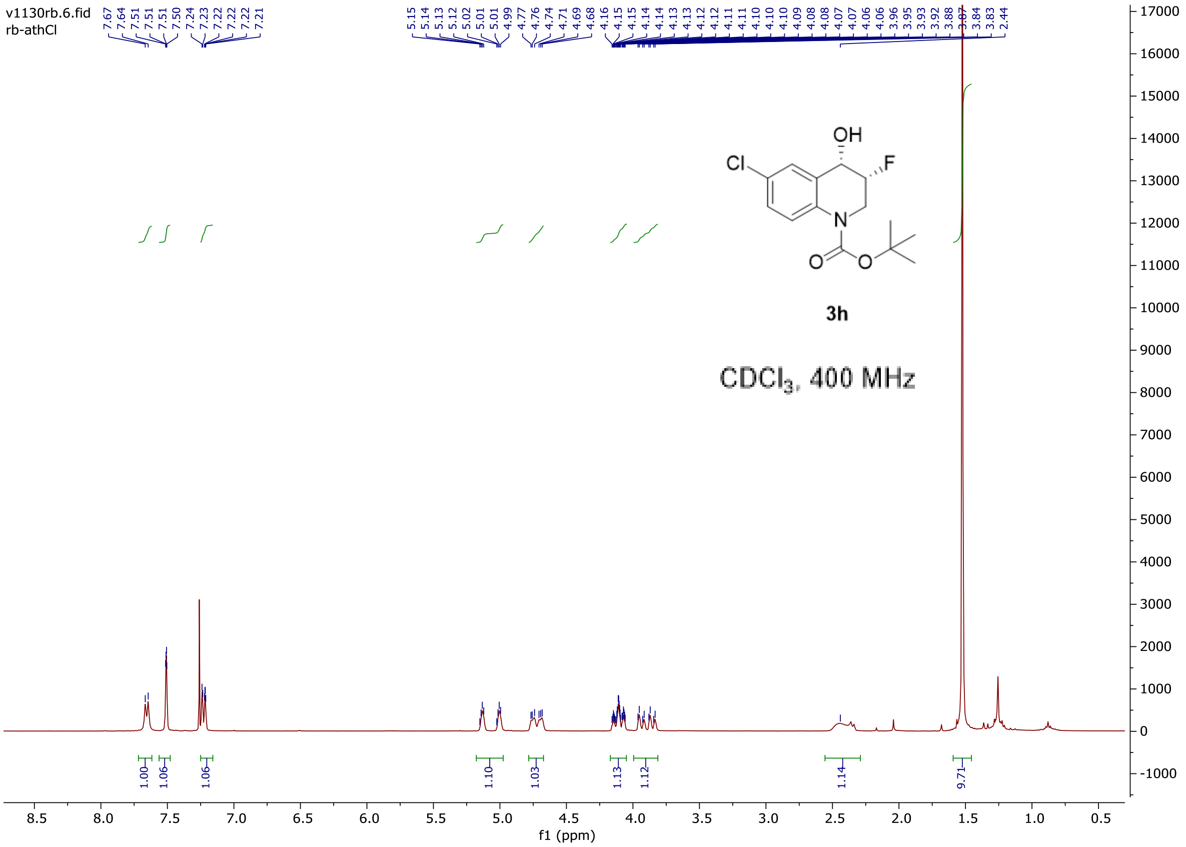
v1130rb.6.fid
rb-athCl
7.67 7.64 7.51 7.51 7.51 7.50 7.24 7.23 7.22 7.22 7.22 7.21
5.15 5.14 5.13 5.12 5.02 5.01 5.01 4.99 4.77 4.76 4.74 4.71 4.69 4.68 4.16 4.15 4.15 4.14 4.14 4.13 4.13 4.12 4.11 4.11 4.10 4.10 4.09 4.08 4.08 4.07 4.07 4.06 4.06 3.96 3.95 3.93 3.92 3.88 3.87 3.84 3.83 2.44
OH
Cl
F
N
O
O
3h
CDCl3, 400 MHz
1.00 1.06 1.06 1.10 1.03 1.13 1.12 1.14 9.71
f1 (ppm)

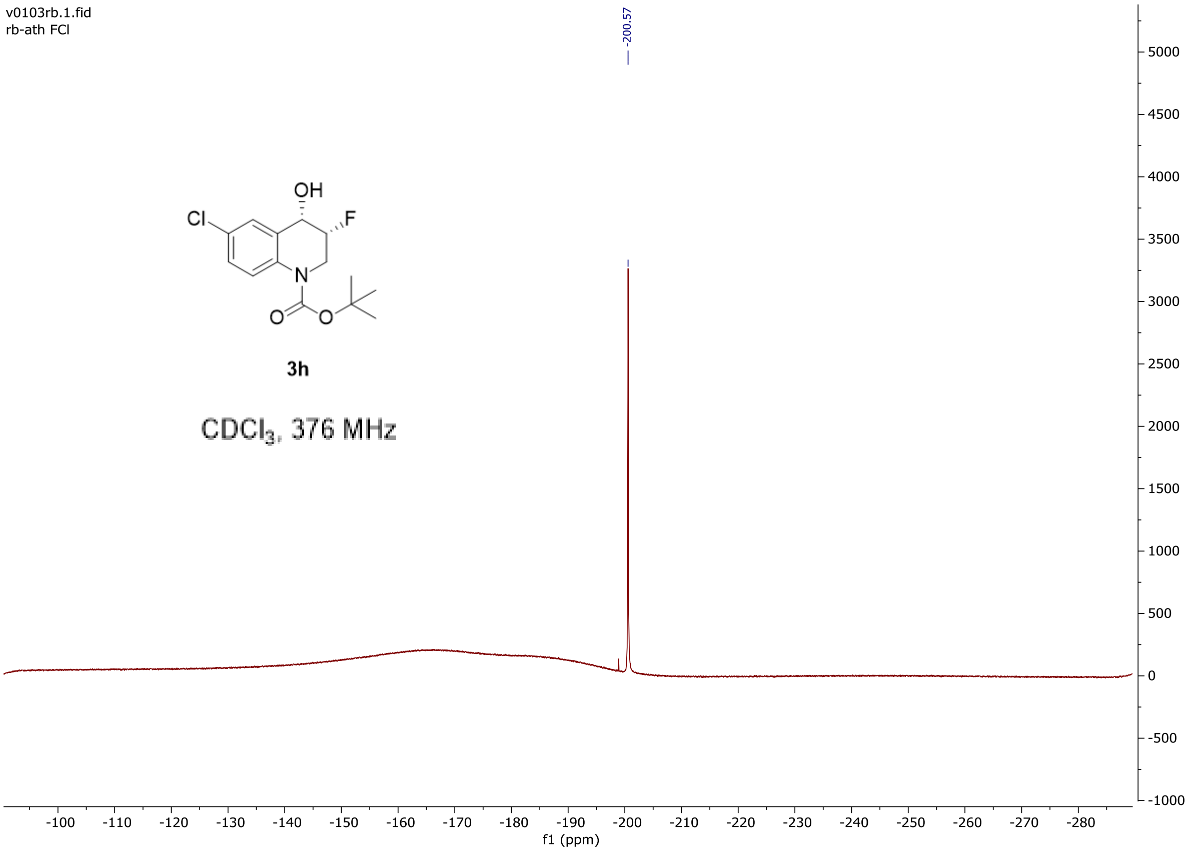
v0103rb.1.fid
rb-ath FCl
-200.57
OH
Cl
F
N
O
O
3h
CDCl3, 376 MHz
f1 (ppm)

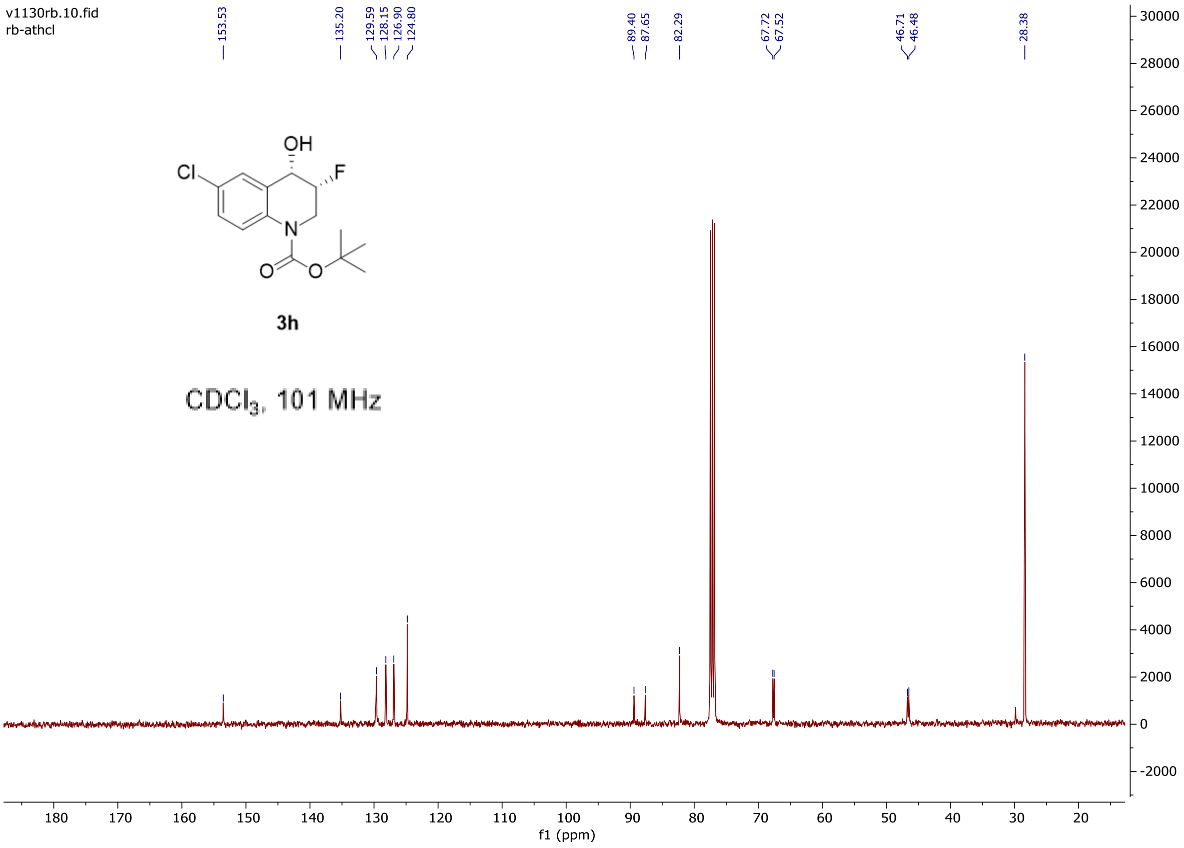
v1130rb.10.fid
rb-athcl
153.53
135.20
129.59
128.15
126.90
124.80
89.40
87.65
82.29
67.72
67.52
46.71
46.48
28.38
OH
F
Cl
N
O
O
3h
CDCl3, 101 MHz
f1 (ppm)

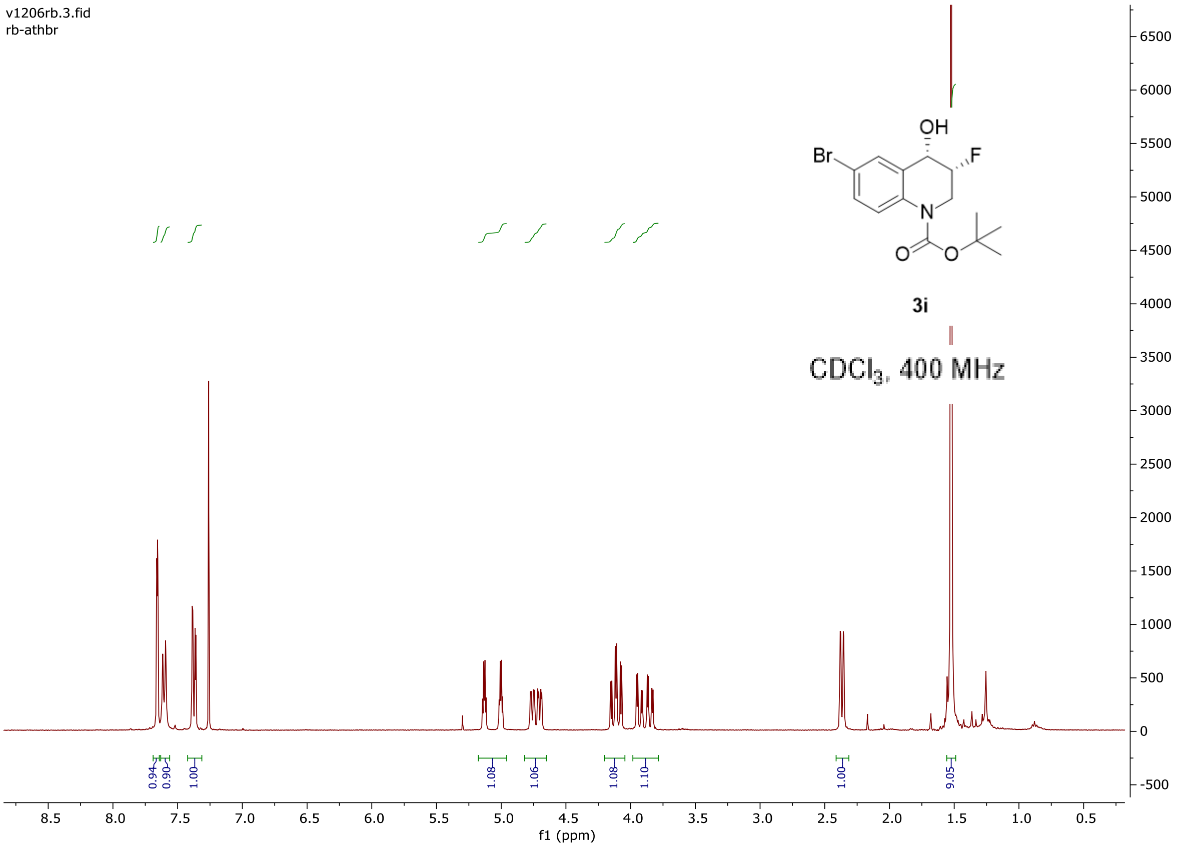
v1206rb.3.fid
rb-athbr
3i
CDCl$_3$, 400 MHz
f1 (ppm)

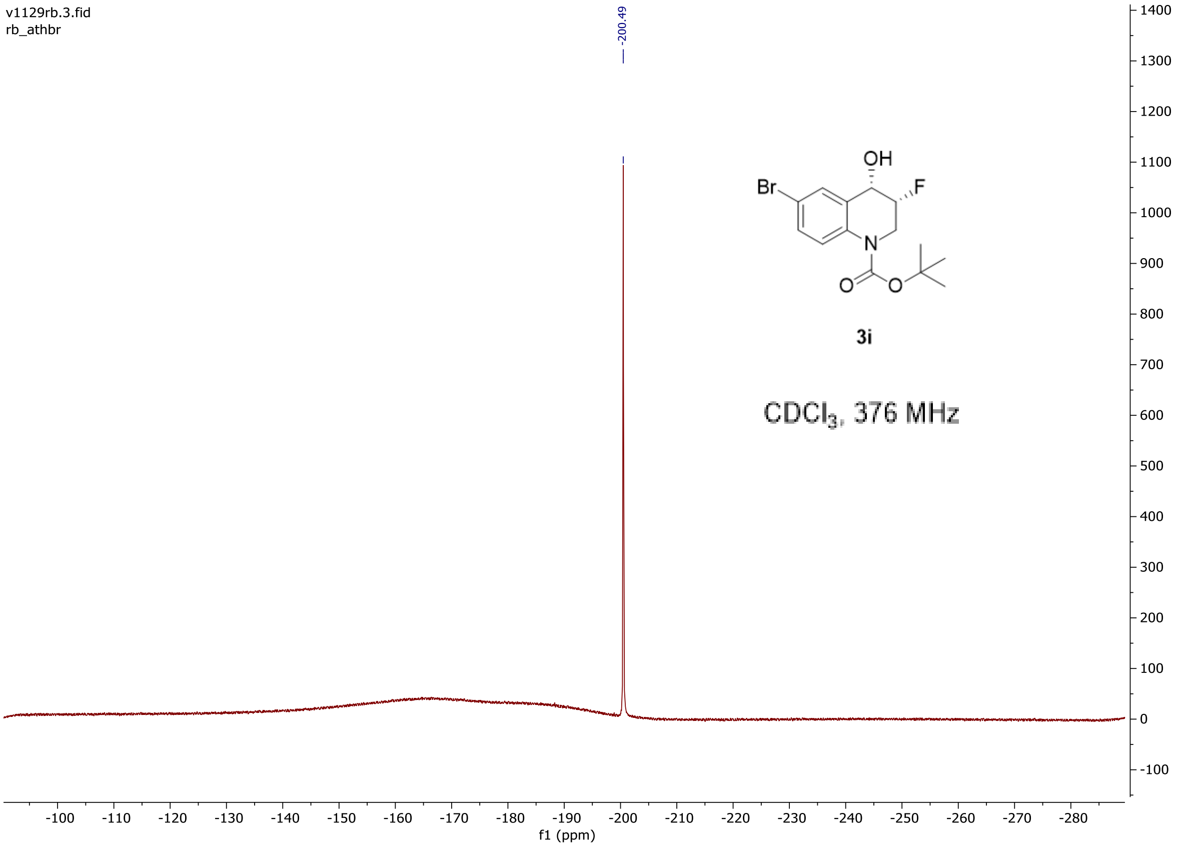
v1129rb.3.fid
rb_athbr
-200.49
3i
CDCl$_3$, 376 MHz
f1 (ppm)

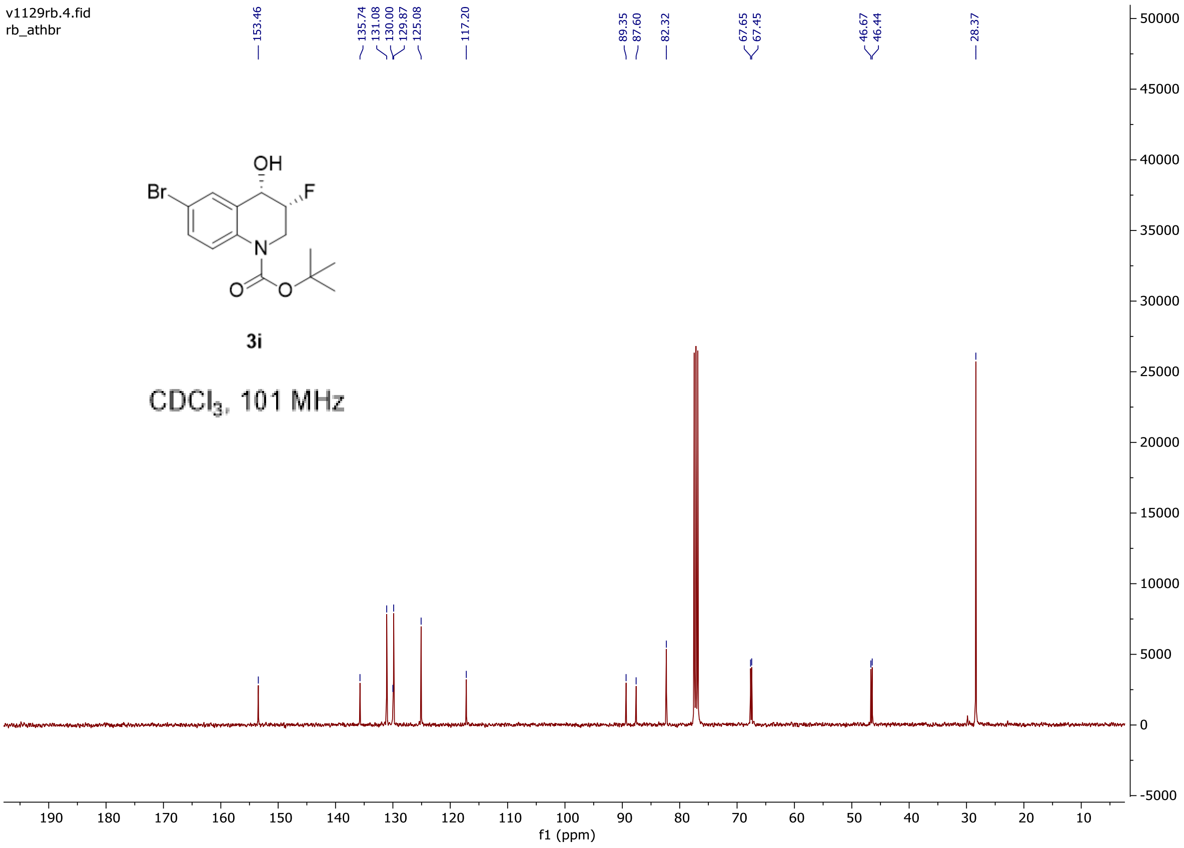
v1129rb.4.fid
rb_athbr
153.46
135.74
131.08
130.00
129.87
125.08
117.20
89.35
87.60
82.32
67.65
67.45
46.67
46.44
28.37
OH
Br
F
N
O
O
3i
CDCl3, 101 MHz
50000
45000
40000
35000
30000
25000
20000
15000
10000
5000
0
-5000
190
180
170
160
150
140
130
120
110
100
90
80
70
60
50
40
30
20
10
f1 (ppm)

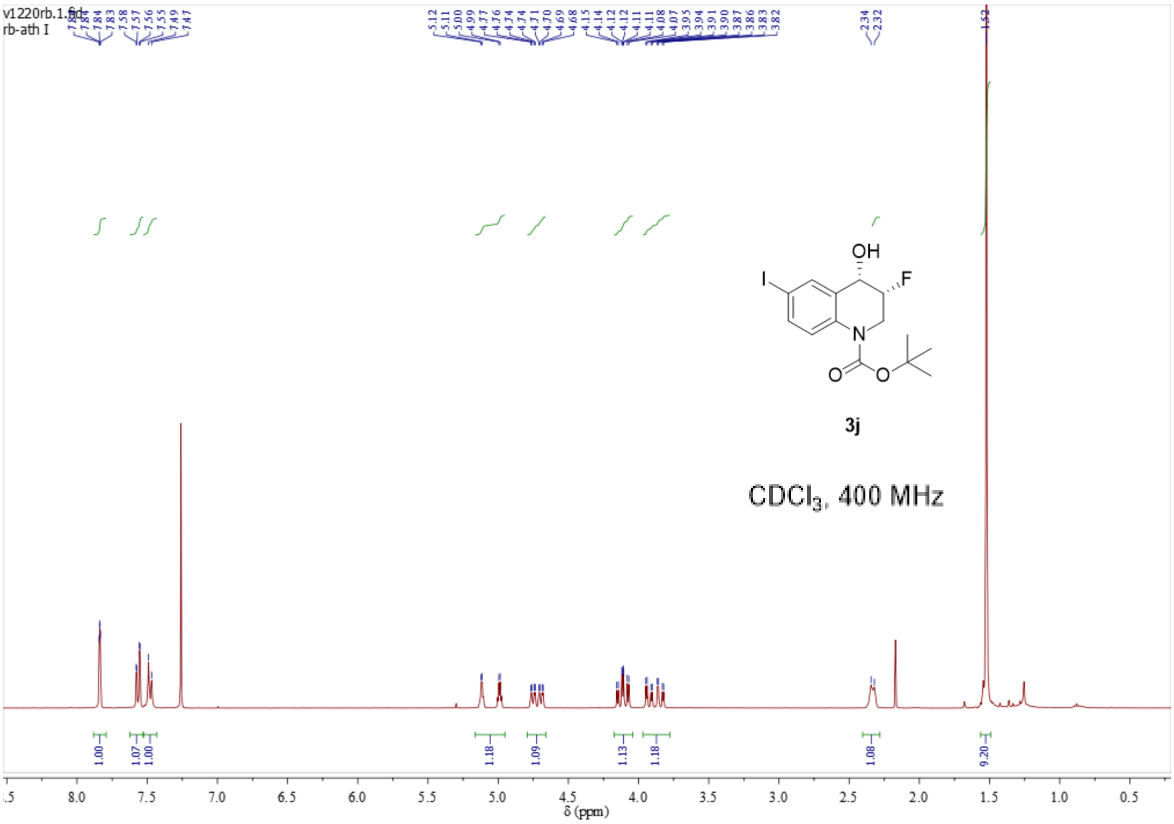
v1220rb.1.fid
rb-ath I
3j
CDCl3, 400 MHz
δ (ppm)

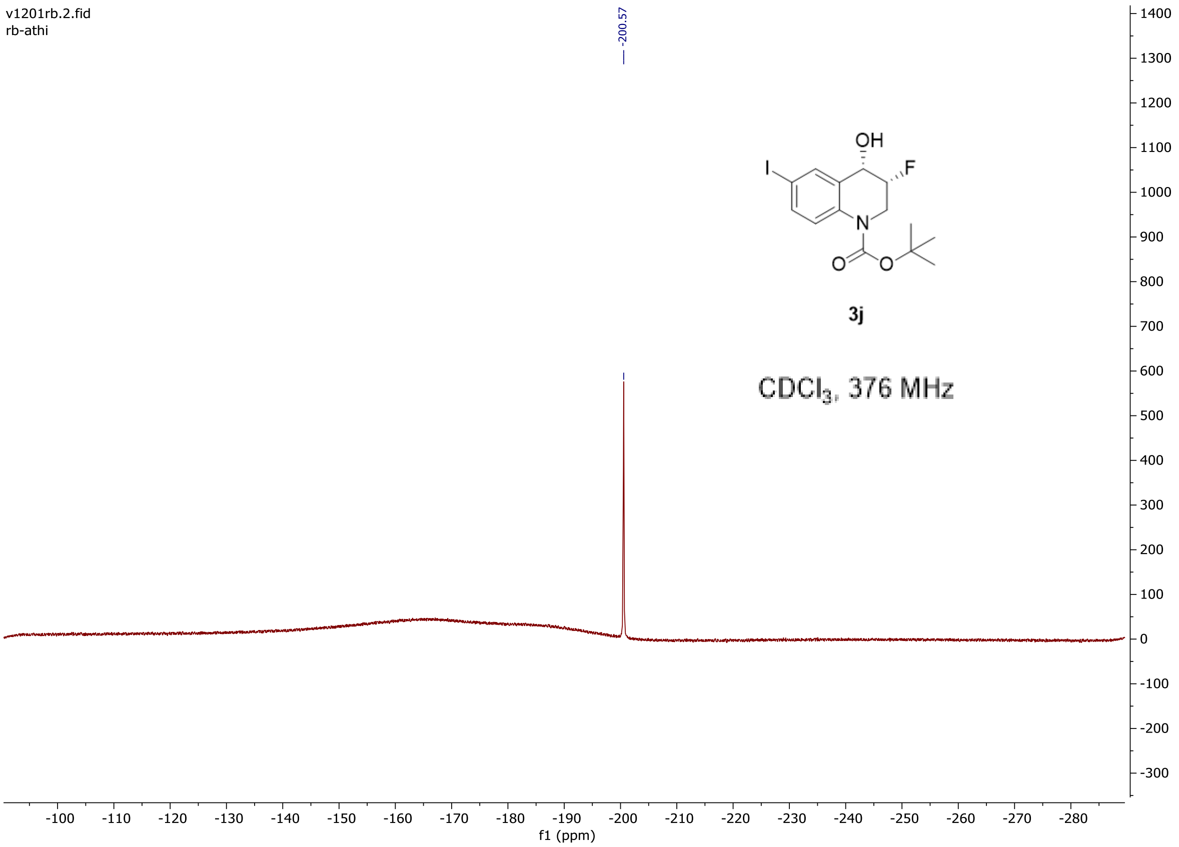
v1201rb.2.fid
rb-athi
-200.57
3j
CDCl3, 376 MHz
f1 (ppm)

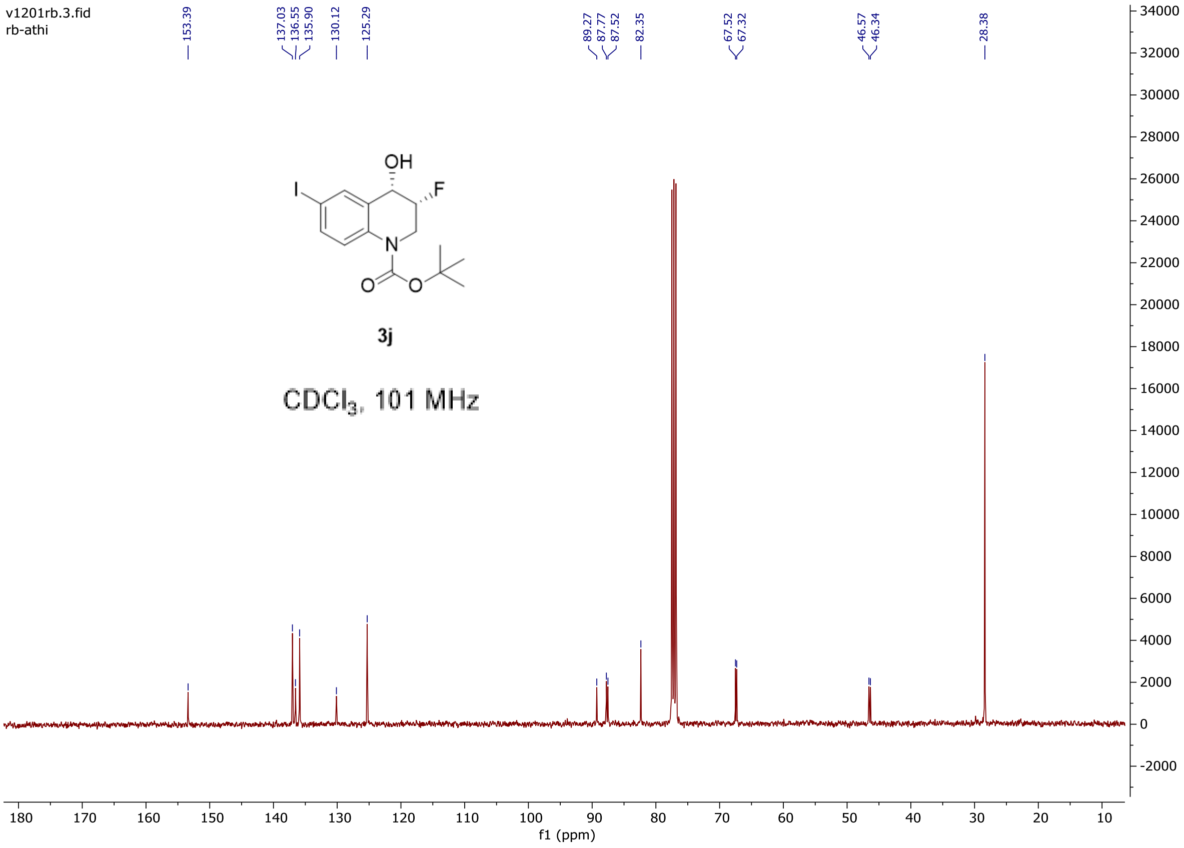
v1201rb.3.fid
rb-athi
153.39
137.03
136.55
135.90
130.12
125.29
89.27
87.77
87.52
82.35
67.52
67.32
46.57
46.34
28.38
OH
F
I
N
O
O
3j
CDCl3, 101 MHz
f1 (ppm)

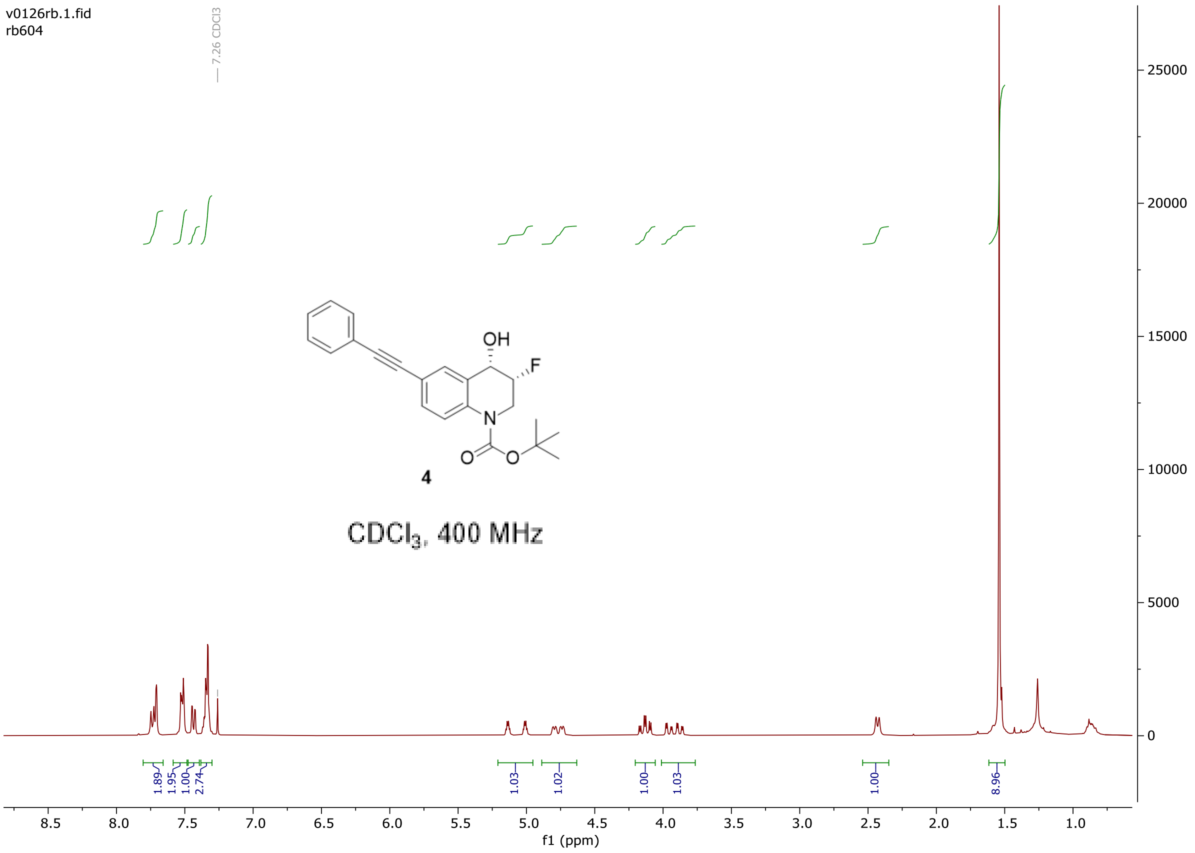
v0126rb.1.fid
rb604
7.26 CDCl3
OH
F
N
O
O
4
CDCl3, 400 MHz
1.89
1.95
1.00
2.74
1.03
1.02
1.00
1.03
1.00
8.96
25000
20000
15000
10000
5000
0
8.5
8.0
7.5
7.0
6.5
6.0
5.5
5.0
4.5
4.0
3.5
3.0
2.5
2.0
1.5
1.0
f1 (ppm)

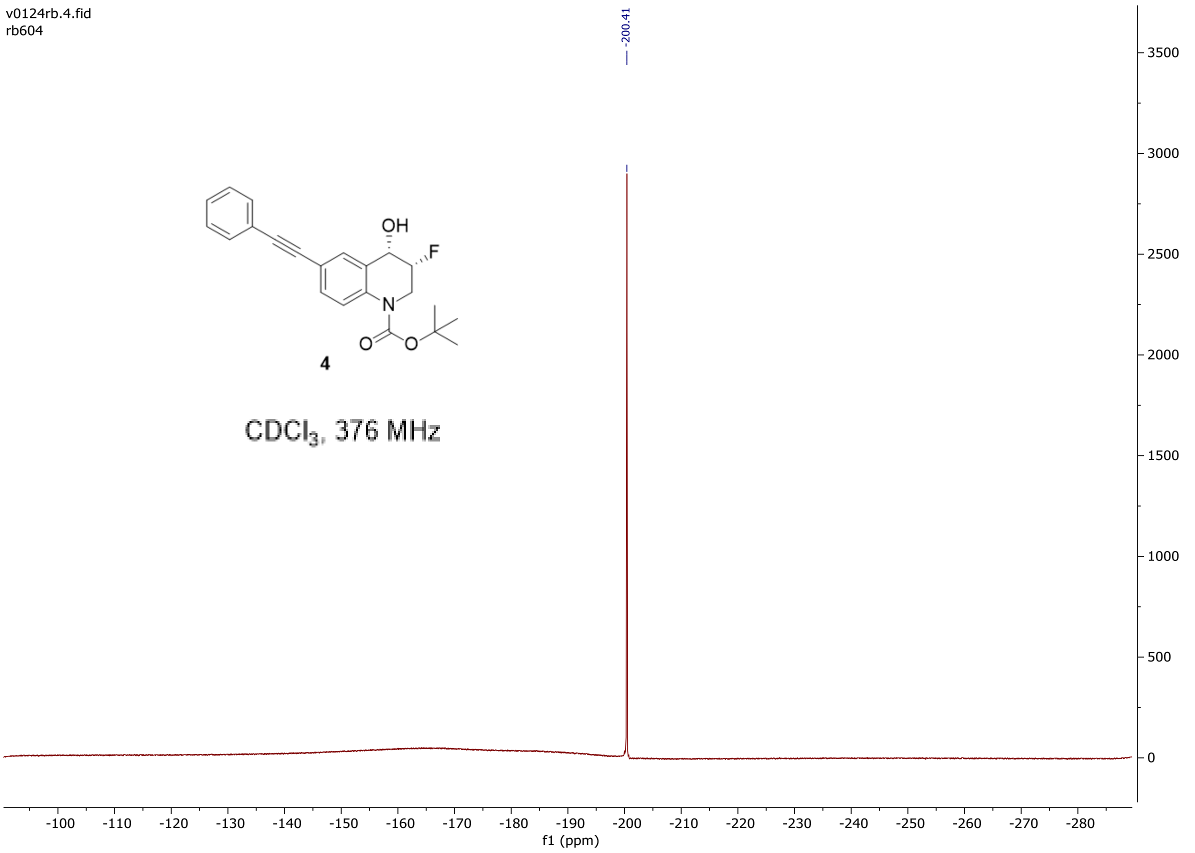
v0124rb.4.fid
rb604
-200.41
OH
F
N
O
O
4
CDCl3, 376 MHz
3500
3000
2500
2000
1500
1000
500
0
-100
-110
-120
-130
-140
-150
-160
-170
-180
-190
-200
-210
-220
-230
-240
-250
-260
-270
-280
f1 (ppm)

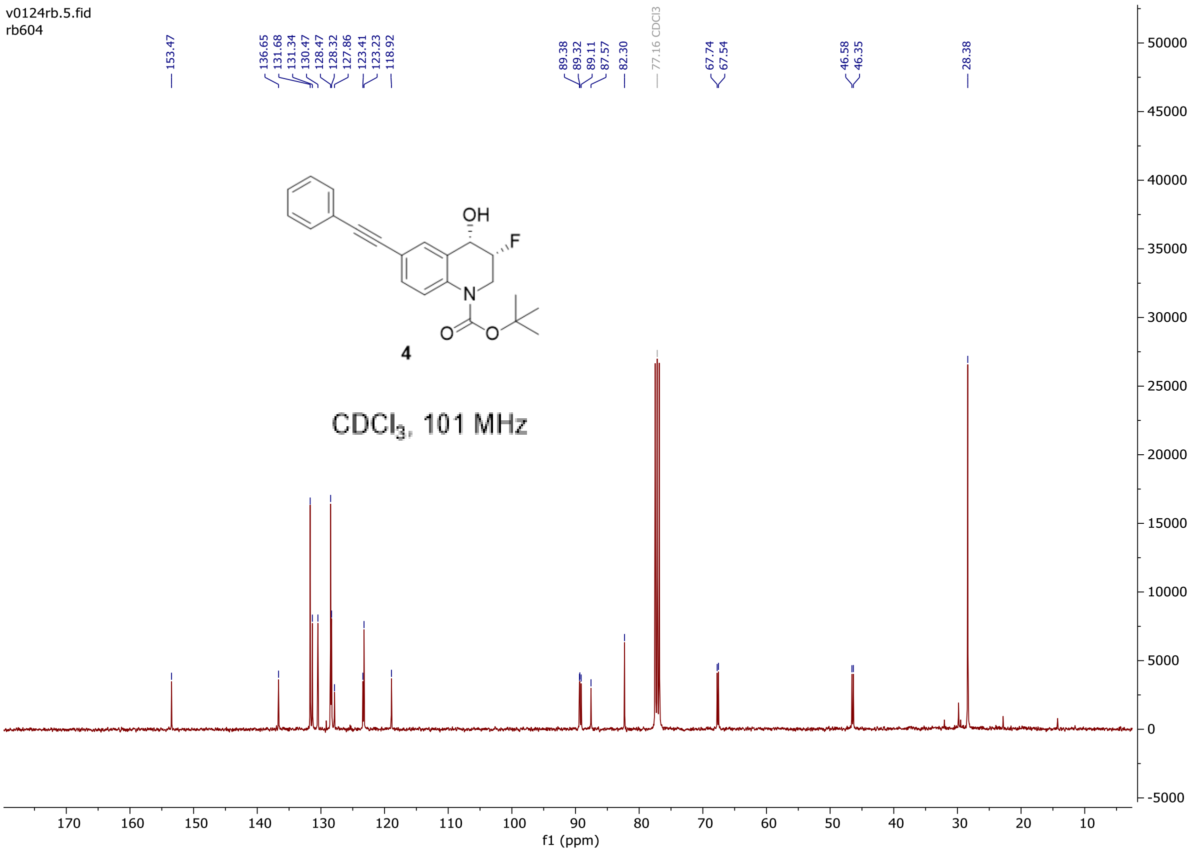

v0124rb.5.fid
rb604
153.47
136.65
131.68
131.34
130.47
128.47
128.32
127.86
123.41
123.23
118.92
89.38
89.32
89.11
87.57
82.30
77.16 CDCl3
67.74
67.54
46.58
46.35
28.38
OH
F
N
O
O
4
CDCl3, 101 MHz
f1 (ppm)

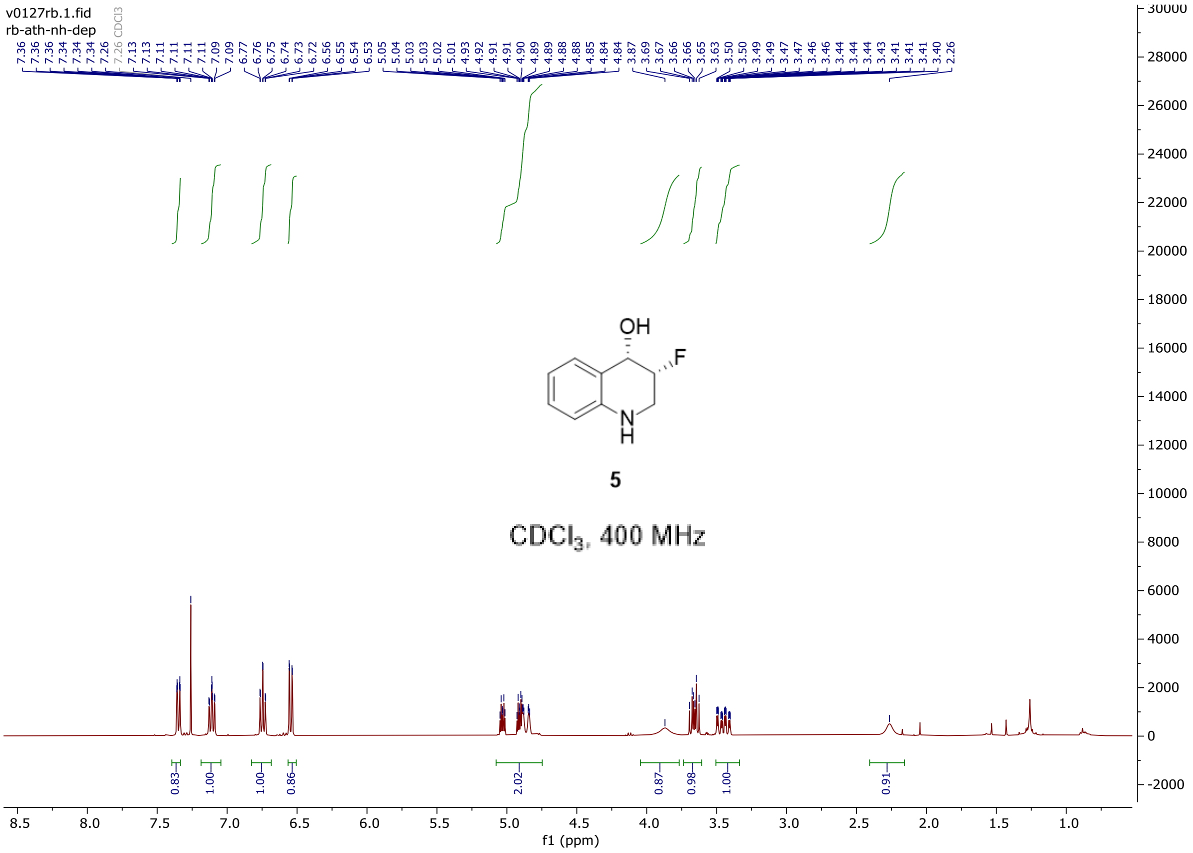
v0127rb.1.fid
rb-ath-nh-dep
7.36 7.36 7.36 7.34 7.34 7.34 7.26 7.26 CDCl3 7.13 7.13 7.11 7.11 7.11 7.11 7.09 7.09 6.77 6.76 6.75 6.74 6.73 6.72 6.56 6.55 6.54 6.53 5.05 5.04 5.03 5.03 5.02 5.01 4.93 4.92 4.91 4.91 4.90 4.89 4.89 4.88 4.88 4.85 4.84 4.84 3.87 3.69 3.67 3.66 3.66 3.65 3.63 3.50 3.50 3.49 3.49 3.47 3.47 3.46 3.46 3.44 3.44 3.44 3.43 3.41 3.41 3.41 3.40 2.26
OH
F
N
H
5
CDCl3, 400 MHz
0.83 1.00 1.00 0.86 2.02 0.87 0.98 1.00 0.91
f1 (ppm)

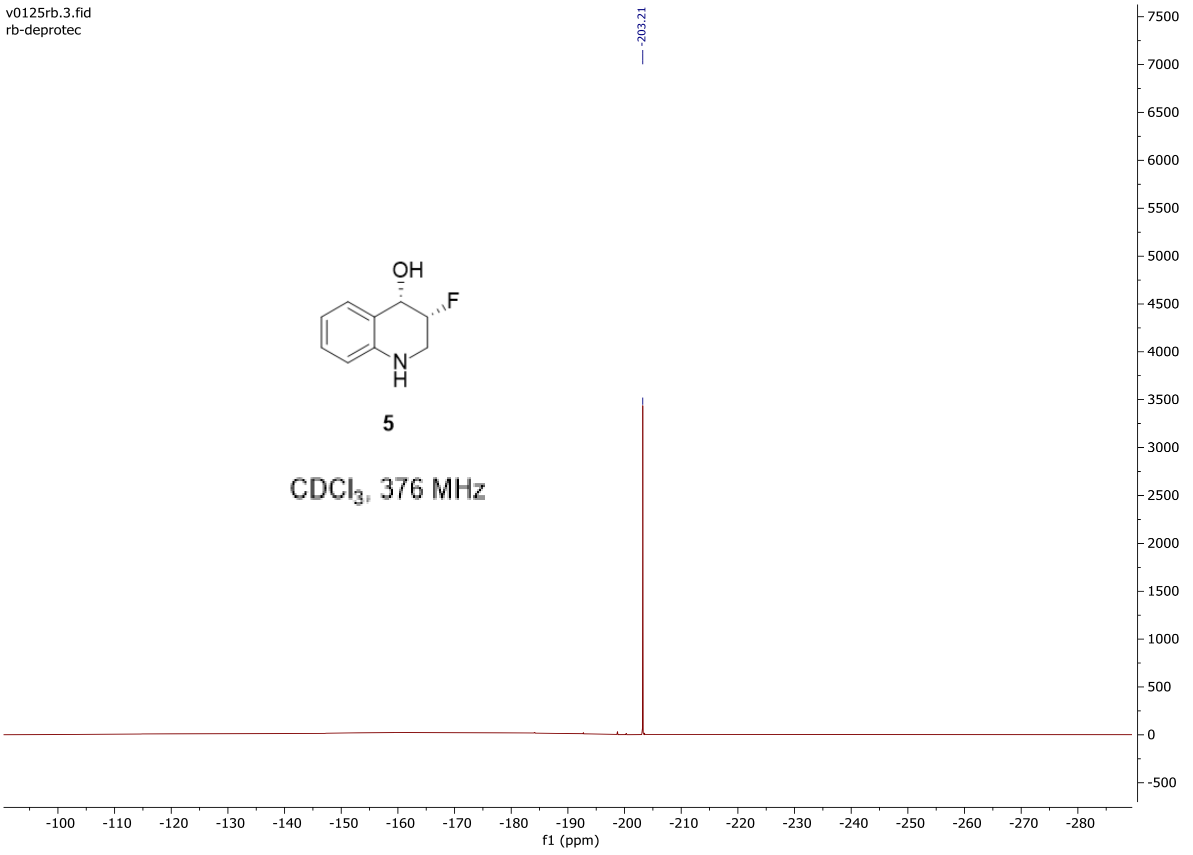
v0125rb.3.fid
rb-deprotec
-203.21
OH
F
N
H
5
CDCl3, 376 MHz
f1 (ppm)

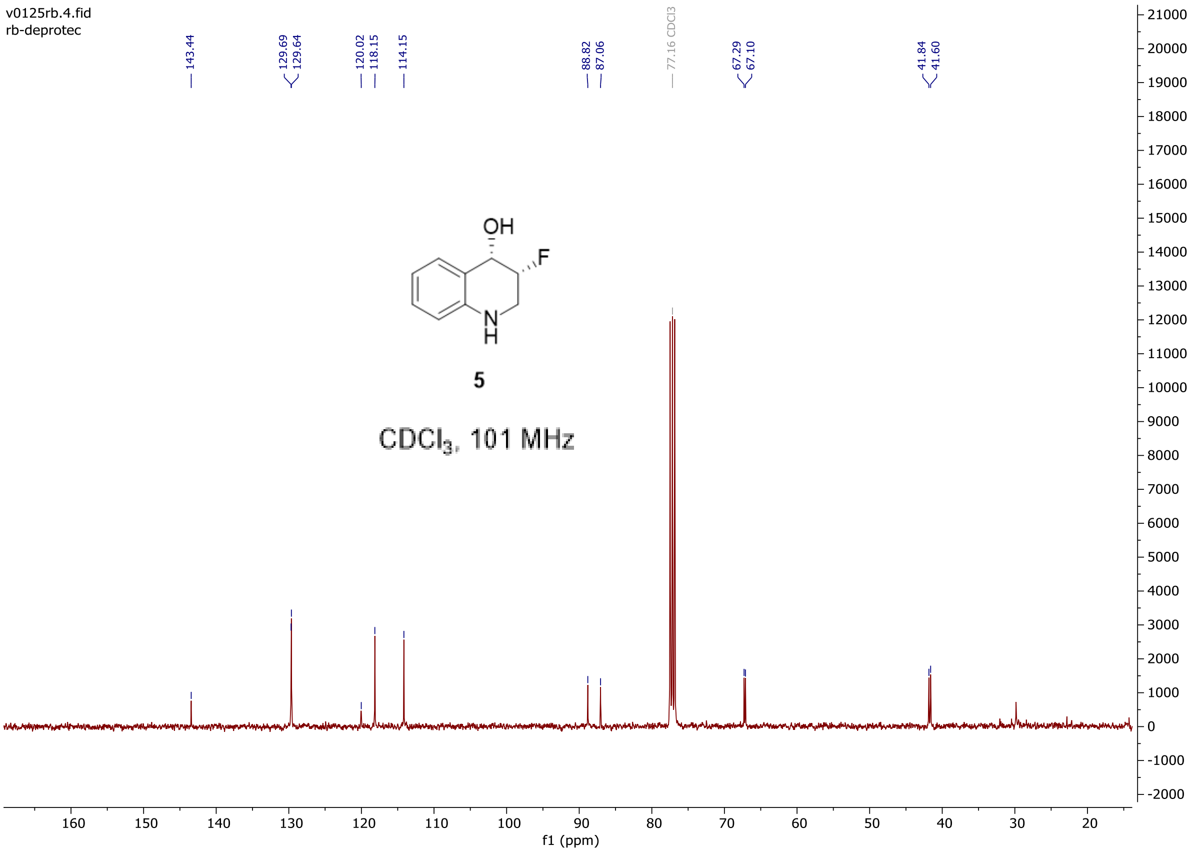
v0125rb.4.fid
rb-deprotec
143.44
129.69
129.64
120.02
118.15
114.15
88.82
87.06
77.16 CDCl3
67.29
67.10
41.84
41.60
OH
F
N
H
5
CDCl3, 101 MHz
f1 (ppm)

## 3) SFC Spectra

Sample Name **RB rac NBocF Ph bis**

Injection Acq Method Name IE - 100 bars - 2 mL.min-1 - 4% EtOH.amx

Injection Acquired Date 2021-12-08 15:21:40+01:00

| *RT* | *Peak Area %* |
|---|---|
| 11.036 | 18.63 |
| 12.848 | 18.59 |
| 14.535 | 31.45 |
| 15.432 | 31.33 |

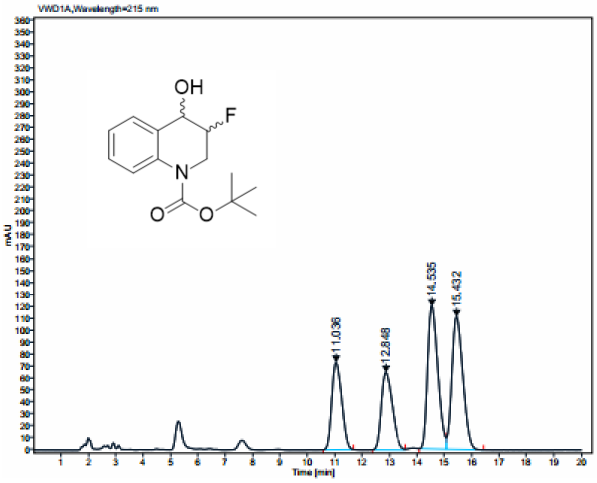


Sample Name **RB ATH NBocF Ph**

Injection Acq Method Name IE - 100 bars - 2 mL.min-1 - 4% EtOH.amx

Injection Acquired Date 2021-12-08 15:50:09+01:00

| *RT* | *Peak Area %* |
|---|---|
| 11.071 | 0.45 |
| 12.480 | 0.00 |
| 14.438 | 99.28 |
| 15.184 | 0.27 |

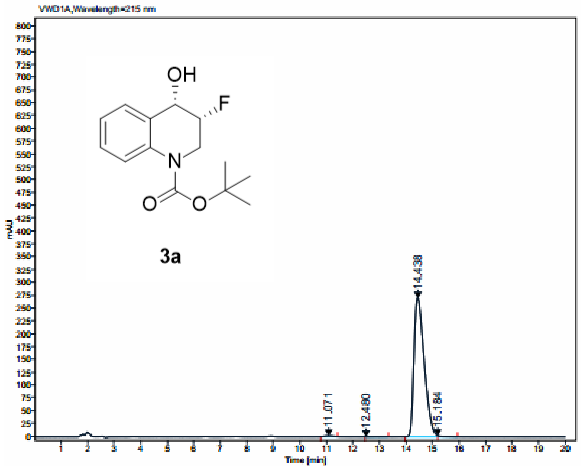

Sample Name **RB rac NBoc F Me bis**

Injection Acq Method Name IE - 100 bars - 2 mL.min-1 - 2.5% EtOH.amx

Injection Acquired Date 2021-12-09 14:48:04+01:00

| RT | Peak Area % |
|---|---|
| 27.713 | 9.08 |
| 32.433 | 40.92 |
| 37.477 | 40.98 |
| 42.406 | 9.03 |

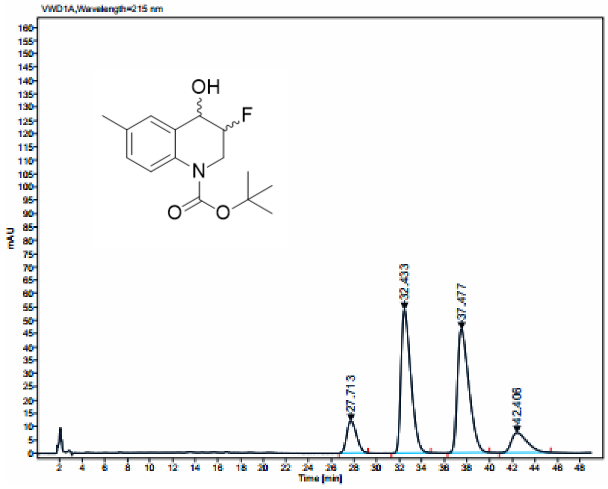


Sample Name **RB ATH NBoc F Me**

Injection Acq Method Name IE - 100 bars - 2 mL.min-1 - 2.5% EtOH.amx

Injection Acquired Date 2021-12-09 16:00:08+01:00

| RT | Peak Area % |
|---|---|
| 27.799 | 1.40 |
| 32.000 | 98.40 |
| 37.932 | 0.18 |
| 42.626 | 0.01 |

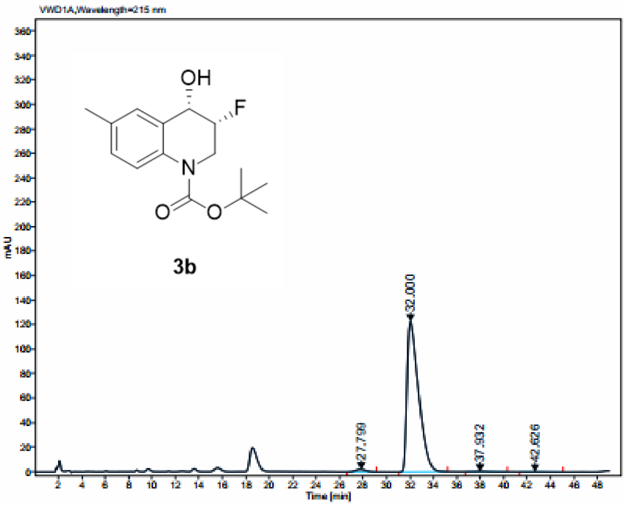

Sample Name **RB rac NBoc F OMe haut**

Injection Acq Method Name IF - 100 bars - 2 mL.min-1 - 4.5% EtOH.amx

Injection Acquired Date 2021-12-10 15:44:50+01:00

| *RT* | *Peak Area %* |
|---|---|
| 15.708 | 3.91 |
| 17.111 | 46.09 |
| 18.688 | 3.92 |
| 22.785 | 46.08 |

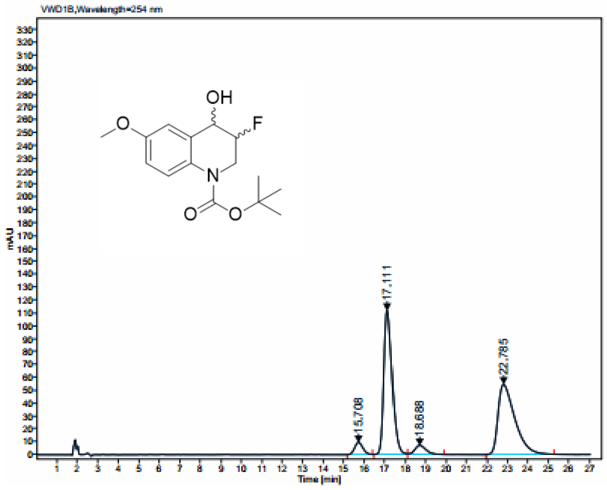


Sample Name **RB ATH NBoc F OMe haut**

Injection Acq Method Name IF - 100 bars - 2 mL.min-1 - 4.5% EtOH.amx

Injection Acquired Date 2021-12-10 16:21:59+01:00

| *RT* | *Peak Area %* |
|---|---|
| 15.641 | 0.57 |
| 17.188 | 0.18 |
| 18.549 | 0.00 |
| 22.652 | 99.25 |

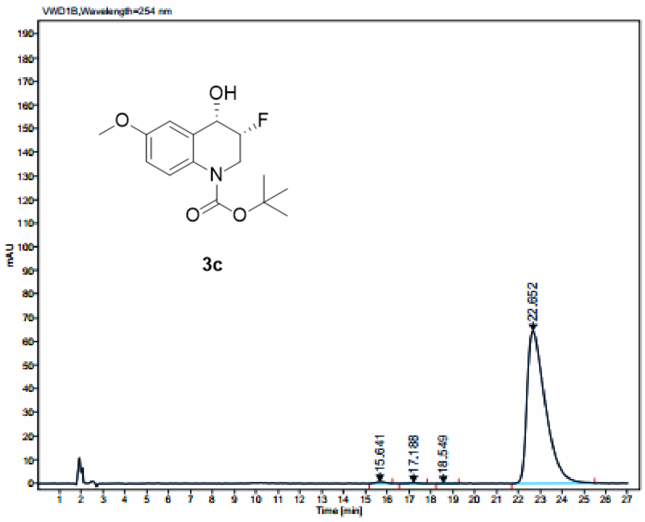

Sample Name **RB rac NBoc F OMe bas**

Injection Acq Method Name IE - 100 bars - 2 mL.min-1 - 5% EtOH.amx

Injection Acquired Date 2021-12-13 15:53:19+01:00

| *RT* | *Peak Area %* |
|---|---|
| 11.865 | 3.60 |
| 13.190 | 3.59 |
| 16.628 | 46.44 |
| 18.621 | 46.37 |

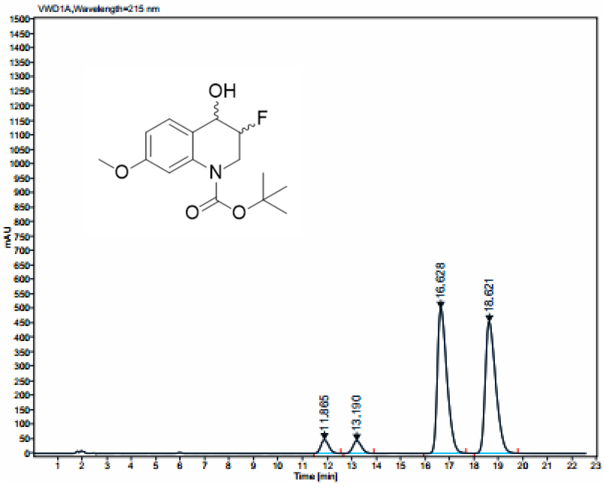


Sample Name **RB ATH NBoc F OMe bas**

Injection Acq Method Name IE - 100 bars - 2 mL.min-1 - 5% EtOH.amx

Injection Acquired Date 2021-12-13 18:46:24+01:00

| *RT* | *Peak Area %* |
|---|---|
| 11.792 | 4.92 |
| 13.079 | 0.01 |
| 16.442 | 94.88 |
| 18.740 | 0.20 |

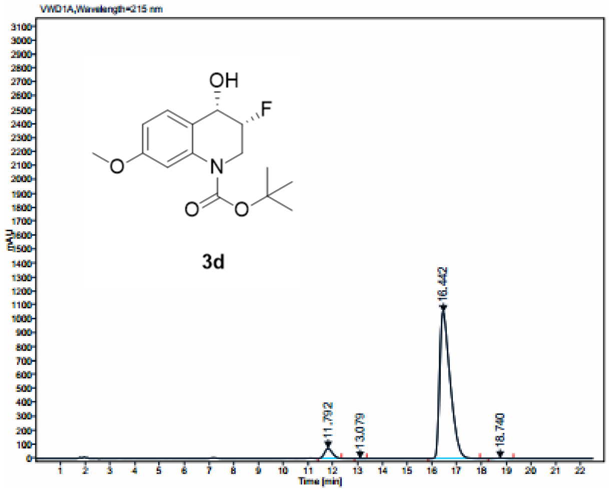

Sample Name **RB rac NBoc F OMe2 bis**

Injection Acq Method Name IF - 100 bars - 2 mL.min-1 - 5% EtOH.amx

Injection Acquired Date 2021-12-13 13:32:06+01:00

| *RT* | *Peak Area %* |
|---|---|
| 14.751 | 5.02 |
| 15.894 | 5.01 |
| 17.454 | 44.97 |
| 19.068 | 44.99 |

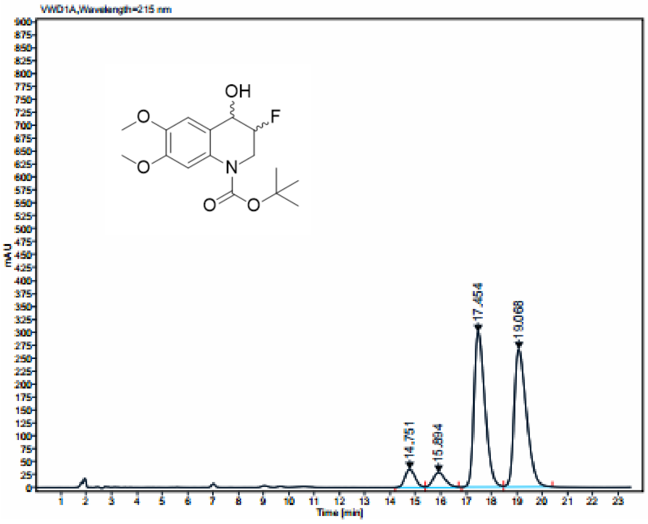


Sample Name **RB ATH NBoc F OMe2**

Injection Acq Method Name IF - 100 bars - 2 mL.min-1 - 5% EtOH.amx

Injection Acquired Date 2021-12-13 14:15:27+01:00

| *RT* | *Peak Area %* |
|---|---|
| 14.742 | 4.50 |
| 15.864 | 0.16 |
| 17.321 | 94.90 |
| 19.238 | 0.45 |

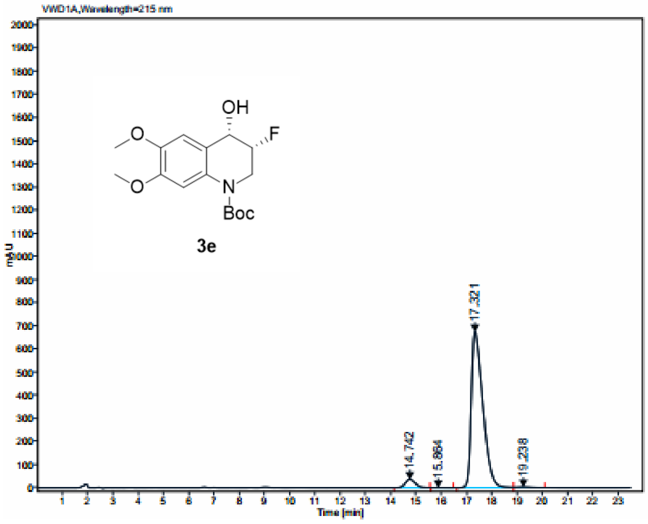

Sample Name **RB rac NBocF F**

Injection Acq Method Name IE - 100 bars - 2 mL.min-1 - 6% MeOH.amx

Injection Acquired Date 2021-12-07 13:07:33+01:00

| *RT* | *Peak Area %* |
|---|---|
| 4.499 | 5.69 |
| 4.959 | 5.58 |
| 6.438 | 44.30 |
| 6.999 | 44.43 |

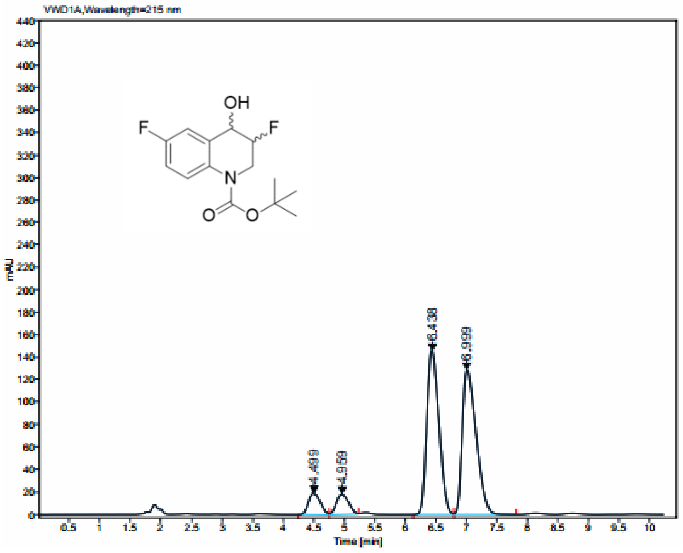


Sample Name **RB ATH NBocF F**

Injection Acq Method Name IE - 100 bars - 2 mL.min-1 - 6% MeOH.amx

Injection Acquired Date 2021-12-07 13:42:07+01:00

| *RT* | *Peak Area %* |
|---|---|
| 4.468 | 0.04 |
| 5.024 | 0.00 |
| 6.451 | 0.25 |
| 6.921 | 99.70 |

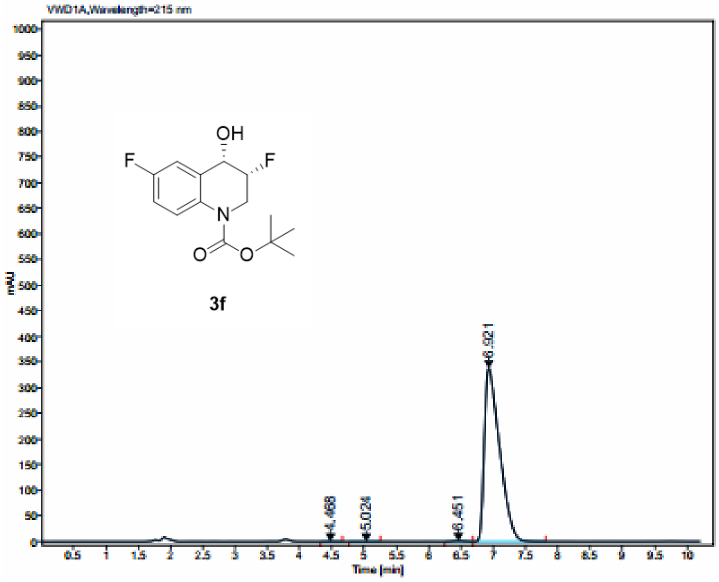

Sample Name **RB rac NBoc F CF3 2**

Injection Acq Method Name IF - 100 bars - 2 mL.min-1 - 4% EtOH.amx

Injection Acquired Date 2021-12-10 17:44:38+01:00

| RT | Peak Area % |
|---|---|
| 5.677 | 49.32 |
| 6.839 | 49.36 |
| 8.525 | 1.33 |

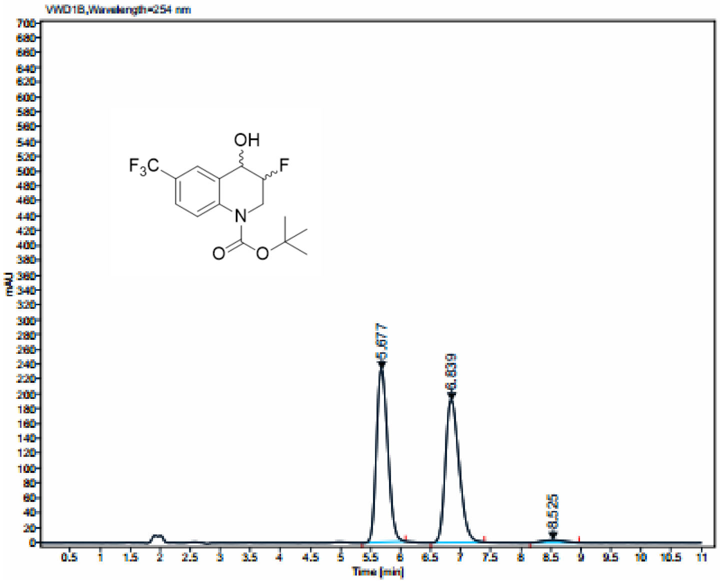


Sample Name **RB ATH NBoc F CF3**

Injection Acq Method Name IF - 100 bars - 2 mL.min-1 - 4% EtOH.amx

Injection Acquired Date 2021-12-10 18:17:50+01:00

| RT | Peak Area % |
|---|---|
| 5.643 | 99.75 |
| 6.847 | 0.14 |
| 8.411 | 0.10 |

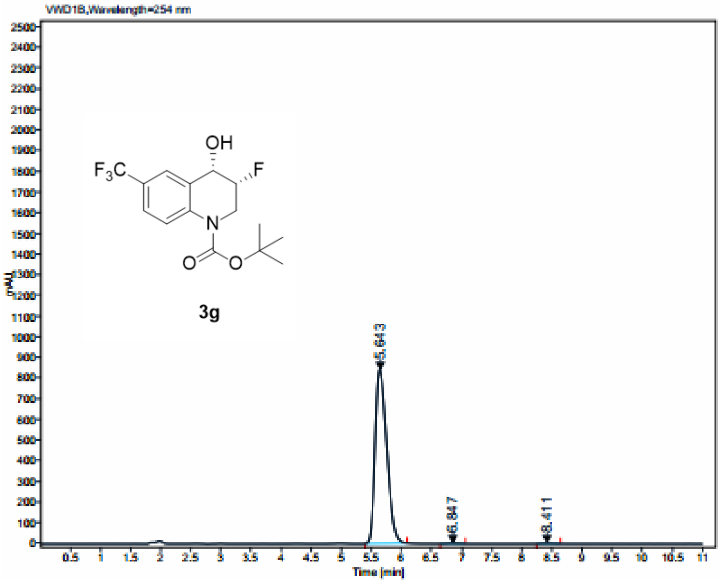

Sample Name **RB rac NBocF Cl**

Injection Acq Method Name IE - 100 bars - 2 mL.min-1 - 6% MeOH.amx

Injection Acquired Date 2021-12-07 10:39:45+01:00

| *RT* | *Peak Area %* |
|---|---|
| 6.952 | 5.27 |
| 7.489 | 5.32 |
| 10.308 | 44.65 |
| 11.028 | 44.76 |

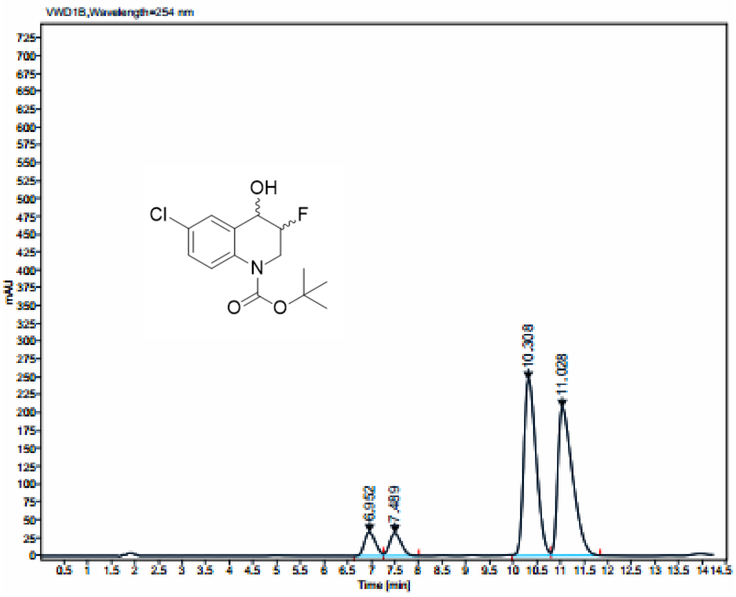


Sample Name **RB ATH NBocF Cl**

Injection Acq Method Name IE - 100 bars - 2 mL.min-1 - 6% MeOH.amx

Injection Acquired Date 2021-12-07 11:10:45+01:00

| *RT* | *Peak Area %* |
|---|---|
| 6.928 | 0.23 |
| 7.501 | 0.00 |
| 10.347 | 0.16 |
| 10.891 | 99.60 |

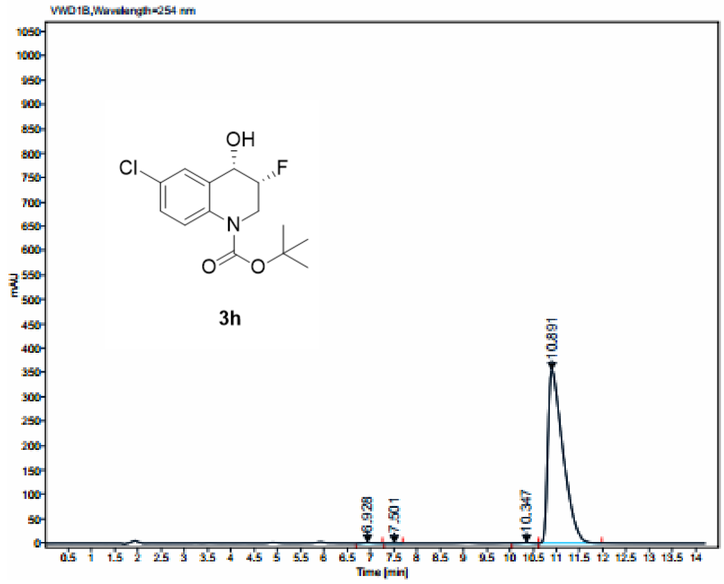

Sample Name **RB rac NBocF Br tris**

Injection Acq Method Name IE - 100 bars - 2 mL.min-1 - 7% MeOH.amx

Injection Acquired Date 2021-12-07 17:47:22+01:00

| *RT* | *Peak Area %* |
|---|---|
| 7.718 | 10.82 |
| 11.074 | 44.61 |
| 11.958 | 44.57 |

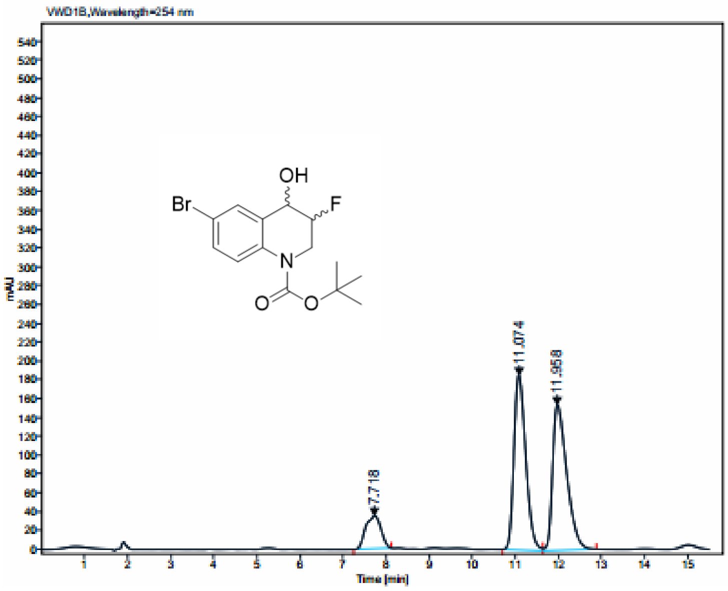


Sample Name **RB ATH NBocF Br**

Injection Acq Method Name IE - 100 bars - 2 mL.min-1 - 7% MeOH.amx

Injection Acquired Date 2021-12-07 17:09:53+01:00

| *RT* | *Peak Area %* |
|---|---|
| 7.107 | 0.05 |
| 7.712 | 0.26 |
| 11.292 | 0.22 |
| 11.878 | 99.47 |

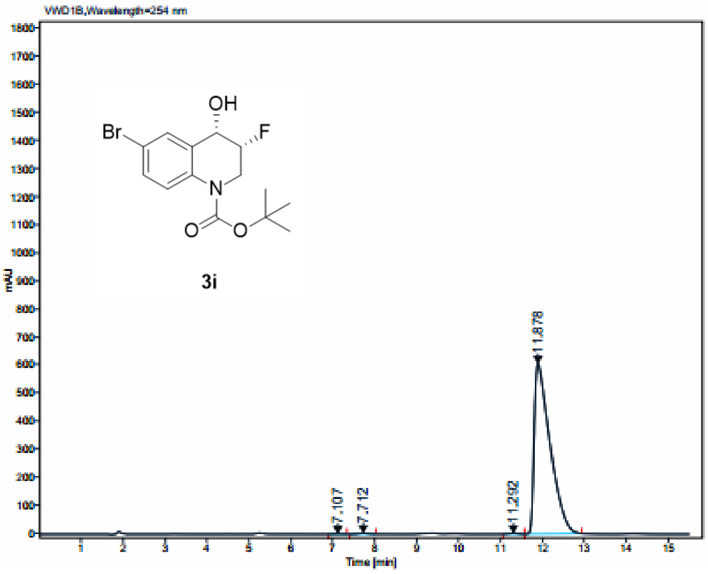

Sample Name **RB rac NBocF I bis**

Injection Acq Method Name IE - 100 bars - 2 mL.min-1 - 7% MeOH.amx

Injection Acquired Date 2021-12-08 09:14:27+01:00

| *RT* | *Peak Area %* |
|---|---|
| 10.923 | 4.42 |
| 11.406 | 4.59 |
| 15.729 | 45.49 |
| 17.254 | 45.49 |

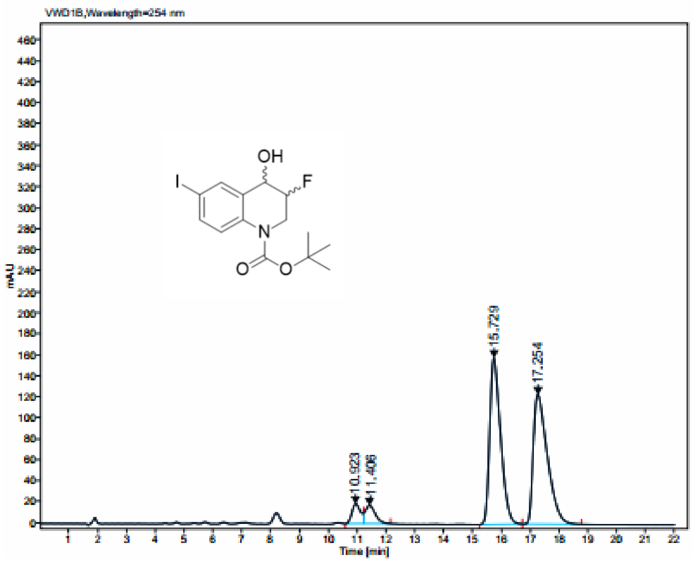


Sample Name **RB ATH NBocF I**

Injection Acq Method Name IE - 100 bars - 2 mL.min-1 - 7% MeOH.amx

Injection Acquired Date 2021-12-08 09:42:09+01:00

| *RT* | *Peak Area %* |
|---|---|
| 10.488 | 0.02 |
| 11.285 | 0.40 |
| 15.814 | 0.22 |
| 16.690 | 99.35 |

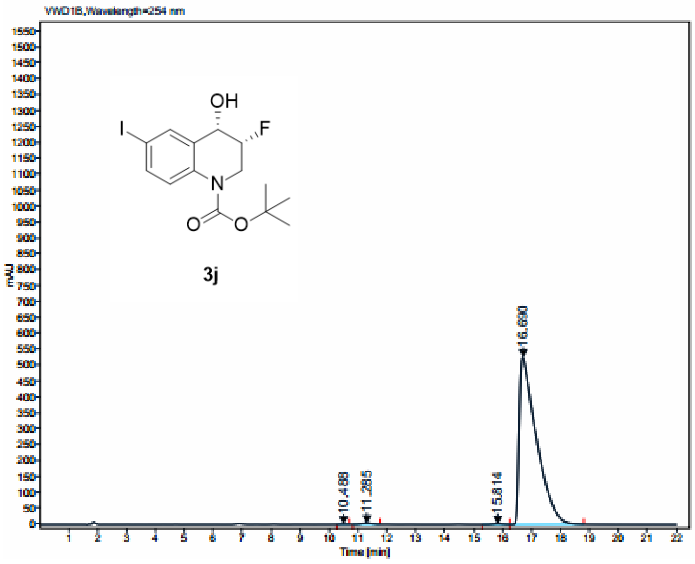

Supplement: Supplementary file 1 [file molecules-27-00995-s001.zip › molecules-1571603-supplementary.pdf]
